# Design, Synthesis, Molecular Docking Study and Biological Evaluation of Novel γ-Carboline Derivatives of Latrepirdine (Dimebon) as Potent Anticancer Agents

**DOI:** 10.3390/molecules28134965

**Published:** 2023-06-24

**Authors:** Ramakrishna Voggu, Arundhati Karmakar, Venkat Swamy Puli, V. Surendra Babu Damerla, Padma Mogili, P. Amaladass, Sridhar Chidara, Kalyan Kumar Pasunooti, Sarika Gupta

**Affiliations:** 1 Department of Medicinal Chemistry, Aragen Life Sciences Pvt. Ltd. (Formerly Known as GVK Biosciences Pvt. Ltd.), IDA, Nacharam, Hyderabad 500076, Telangana, India; ramakrishna.voggu@aragen.com (R.V.); venkatswamypuli@gmail.com (V.S.P.); venkata.damerla@aragen.com (V.S.B.D.); 2 Department of Engineering Chemistry, Andhra University, Visakhapatnam 530003, Andhra Pradesh, India; mpadma.aueng@gmail.com; 3 Molecular Science Laboratory, National Institute of Immunology, New Delhi 110067, India; arundhatik@nii.ac.in; 4 Department of Chemistry, Madanapalle Institute of Technology & Science, Madanapalle 517325, Andhra Pradesh, India; amaladass@mits.ac.in; 5 ProSAM Bioscience Pvt. Ltd., Hyderabad 500049, Telangana, India; 6 Department of Pharmacology and Molecular Sciences, Johns Hopkins School of Medicine, Baltimore, MD 21205, USA

**Keywords:** Latrepirdine (Dimebon), γ-Carboline, cancer cell line, molecular docking, structure-activity relationship, anticancer activity

## Abstract

A series of novel γ-Carboline derivatives were designed and synthesized using the Suzuki coupling reaction to identify the leads for the activity against cancer. Interestingly, these compounds were tested for their anticancer activity against the cell lines, particularly human cancer cell lines MCF7 (breast), A549 (lung), SiHa (cervix), and Colo-205 (colon). Most of the γ-Carboline derivatives showed potent inhibitory activity in four cancer cell lines, according to in vitro anticancer activity screening. Two compounds, specifically **LP-14** and **LP-15**, showed superior activity in cancer cell lines among the γ-Carboline derivatives from **LP-1** to **LP-16**. Additionally, the compound **LP-14**, **LP-15** and Etoposide carried out molecular docking studies on human topoisomerase II beta in complex with DNA and Etoposide (PDB ID: 3QX3). The docking studies’ results showed that the derivative **LP-15** was strongly bound with the receptor amino acid residues, including Glu477 and DC8 compared with the marked drug Etoposide.

## 1. Introduction

γ-Carboline derivatives comprise an important class of bioactive scaffolds that have received great attention in recent years for the treatment of various diseases, particularly neurodegenerative diseases (CNS) [1,2,3]. In addition, these pharmacophores possess various properties, such as anti-histamine, anti-depressant, and anti-inflammatory [4]. Latrepirdine (Dimebon) is a close analog of γ-Carboline and is utilized as an anti-histamine medication in clinical settings [3,5,6,7,8]. However, Pfizer recently suffered a severe failure with Latrepirdine in phase III clinical trials for treating Alzheimer’s disease [9]. Latrepirdine is an anti-histamine and a powerful 5-HT6 inhibitor, with a wide range of pharmacological properties at numerous receptors. Latrepirdine is known to block the function of neurotoxic beta-amyloid proteins [10]. It also inhibits L-type calcium channels and modifies the activity of AMPA and NMDA glutamate receptors [11]. Previously, a few research groups reported that γ-Carboline derivatives were identified as potent histone deacetylase (HDAC) inhibitors with neuroprotective properties [12]. HDAC inhibitors have been identified as promising drug candidates for several diseases, mainly in the treatment of cancer, neurodegeneration, and inflammation [13]. Cancer is one of the most prevalent diseases affecting people, a multi-step illness influenced by physical, chemical, environmental, metabolic, and hereditary elements [14,15]. Currently, the risk of cancer is continuously increasing due to changes in environmental and lifestyle factors, but at the same time, cancer mortality is decreasing, which is attributable to the outstanding progress gained in recent years. Cancer treatment has traditionally been regarded as one of the most important and crucial clinical challenges. Several treatments, including surgical resection, radiotherapy, chemotherapy, and immunotherapy, have been developed, with chemotherapy being the most popular method. Unfortunately, due to compromised immune escape systems or malfunctioning cell death mechanisms, cancer cells frequently develop resistance to therapy, and anticancer medications typically have negative side effects that are severe, debilitating, and unwelcome. Therefore, developing new, more potent, and less harmful anticancer therapies is imperative.

**Figure 1:**
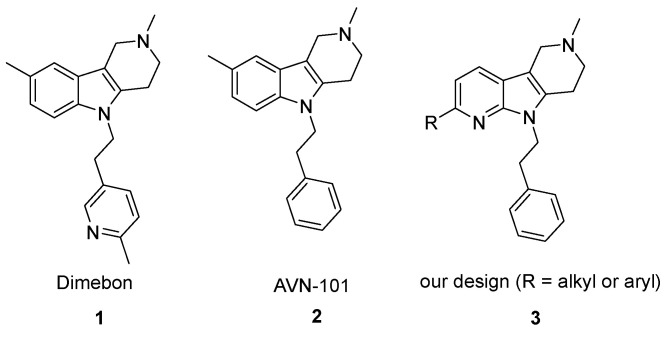
Chemical structures of the bioactive γ-Carbolines analogs.

γ-Carboline is a favored structure that has been extensively employed in medicinal chemistry for drug discovery as an efficient template [16]. The potential biological applications of γ-Carboline derivatives are CNS drug candidates, which are illustrated by Dimebon (**1**) and AVN-101 (**2**) in Figure 1. The exploration of γ-Carboline-based anticancer medicines have been very limited with respect to usage in anticancer chemotherapy [17]. In this context, we describe the synthesis, molecular docking studies, biological evaluation, and structure-activity relationships of a series of novel Latrepirdine derivatives as anticancer agents. The exciting medicinal activity and anticancer properties of γ-carboline derivatives prompted us to further explore the synthesis of various γ-Carboline derivatives, especially Latrepirdine derivatives. Different γ-Carboline derivatives (**3** in Figure 1) were designed and synthesized as anticancer medicines in this work using Suzuki coupling methods. In addition, the effectiveness of these substances against the human cancer cell lines MCF7 (breast), A549 (lung), SiHa (cervix), and Colo-205 (colon) was examined. The results of the docking studies demonstrated that the Latrepirdine derivative (**2** in Figure 1) had a stronger affinity for the receptors than Latrepirdine.

## 2. Results and Discussion

### 2.1. Synthesis

**Scheme 1:**
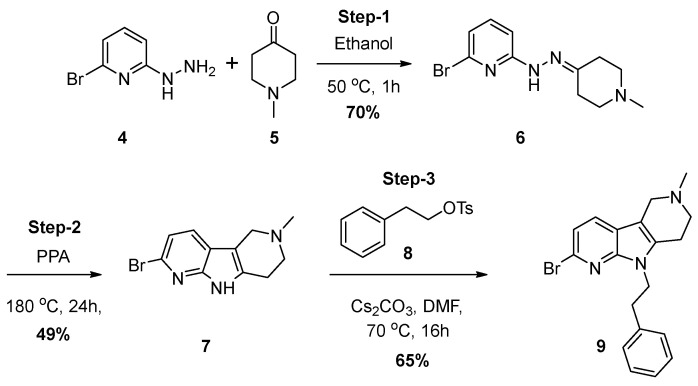
Synthetic route for the key intermediate **9** for the preparation of compounds **LP-1** to **LP-16**.

**Scheme 2:**
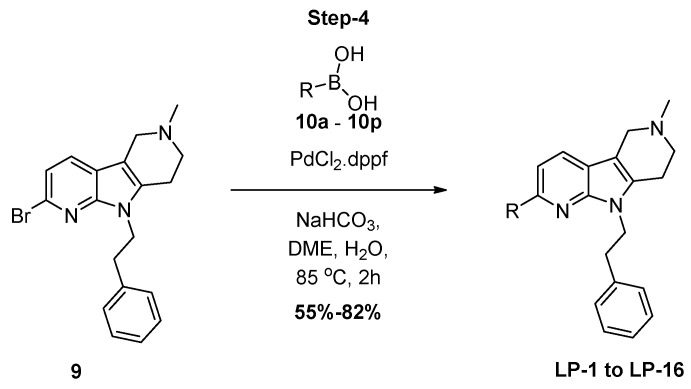
Synthesis of compounds **LP-1** to **LP-16**.

Our initial focus was on the synthesis of γ-Carboline derivative **9**, which is the significant derivative to synthesize all other γ-Carboline derivatives (Scheme 1). To obtain compound 9, the synthesis of imine 6 as an intermediate was synthesized using a condensation reaction between 2-bromo-6-hydrazinylpyridine (**4**) and 1-methylpiperidin-4-one (**5**). Imine 6, reacted with hot PPA at 180 °C for 24 h which produced γ-Carboline **7** [18,19]. *N*-alkylation of compound **7** was achieved using a cesium carbonate base. The complete synthetic route of compound **9** was shown in Scheme 1. The γ-Carboline derivatives LP-1 to LP-16 were synthesized using the Suzuki coupling reaction. Compound 9 treated with various alkyl (or) aryl-boronic acids with the Palladium catalyst using DME as solvent yielded corresponding γ-Carboline derivatives. The yield of the products was approximately 50% to 75%. Scheme 2 illustrates the synthetic route for several γ-Carboline derivatives [20]. Table 1 shows the various derivatives of synthesized γ-Carboline derivatives. All the compounds were fully characterized by ^1^H and ^13^C NMR, ESI-MS, and IR spectral data which are provided in the Supporting Information. The analytical data consist of the target molecular structures. 

### 2.2. In Vitro Cytotoxic Activity

The activities of all the synthesized γ-Carboline derivatives (**LP-1** to **LP-16**) were tested against a series of anticancer cell lines. The structure-activity relationships (SARs) were established from the anticancer data reported in Table 1. Initially, phenyl substituent on γ-Carboline (**LP-1**) exhibited decent activity against three different anticancer cell lines ranging from 3 to 5 µM. Further, we introduced fluorine at different positions of the phenyl group, but the activity could not be retained (entries **2**, **3**, **6**, **7**, and **11**). Together, the cyano substitution of the phenyl group at positions 3 and 4 did not show any impact on the anticancer activity (entries 4 and 8). Surprisingly, among all substitutions (Cl, F, CN, naphthyl, methoxy, furyl and pyridyl) that conducted systematic SAR, the most active compounds **LP-14** and **LP-15** of pyridine and chloro substitutions showed the most potent activity. In addition, the thiophene-substituted compound **LP-13** also showed better activity with a series of anticancer cell lines. Chloro substitution analog at the phenyl part was found to be the most active of the series. In summary, the information on SAR provided us with a guideline to improve anticancer activity in future structural modification.

### 2.3. Molecular Docking Studies

**Figure 2:**
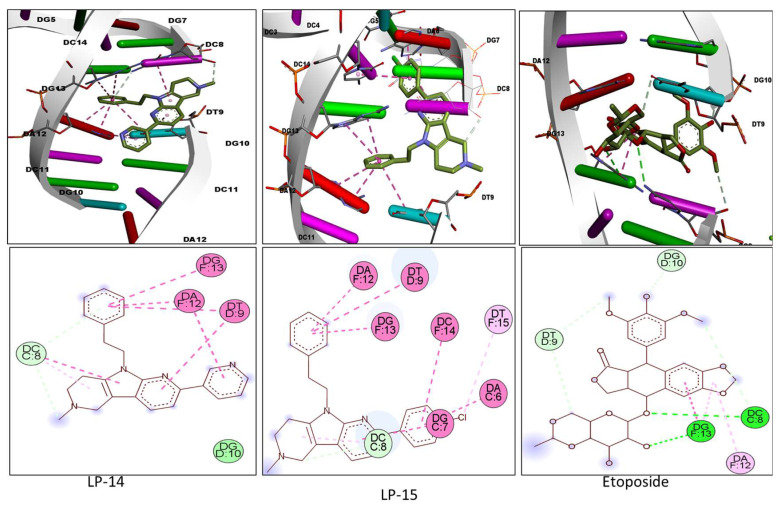
Molecular docking studies of compounds **LP-14**, **LP-15** and etoposide with human topoisomerase II beta in complex with DNA (PDB ID: 3QX3).

Compound **LP-14** showed similar binding interactions inside the binding pocket of topo II as etoposide, with a docking score of −8.89 kcal/mol. As illustrated in Figure 2, the compound showed an H-bond interaction DC8. The tricyclic ring was intercalated with DT9, DA12, DG13, and DC8. It also formed a hydrophobic interaction with Arg503 residue. Similarly, **LP15** exhibited the same interactions, with an H-bond with Glu477 and DC8. The tricyclic ring was intercalated with DT9, DA12, DG13, DA6, DG7, and DT15 with a docking score of −9.65 kcal/mol (Table 2). The ring was pi–pi stacked with DT9, DA12, and DG13 and pi–pi T-shaped with DG7 and DC14. Pi–alkyl interactions were observed with DC8 and DT15. 

In addition, both **LP-14** and **LP-15** showed similar binding patterns to etoposide, with interactions involving H bonds, intercalation, and hydrophobic interactions. **LP-14** showed an H-bond interaction with DC8 and intercalation with several nucleotides, while **LP-15** formed an H bond with Glu477 and intercalated with more nucleotides than **LP-14**. The pi–pi stacking and pi–alkyl interactions observed in both compounds also contributed to their binding affinity to the topo II enzyme.

### 2.4. Biological Perspective

Topoisomerases are one of the most important enzymes required for maintaining the integrity and topology of the DNA double helix structure. During the processes of unwinding and winding in DNA replication, repair, and recombination in actively dividing cells, the long DNA is prone to entanglements, which can lead to chromosome breakage and cell death if not repaired. Therefore, topoisomerases have become highly attractive targets in cancer treatment as their levels are found to be elevated in cancer cells; in fact, their levels determine the effectiveness of chemotherapy [21].

**LP-14** has been observed to have cytotoxic effects on the four different cell lines at low concentrations (low IC_50_ values) and its effectiveness may be due to the pyridine substitution at the phenyl ring. Pyridine is a bio-isostere of benzene with one C replaced with N. The heterocyclic pyridine nucleus had been studied for a long time and shown to display diverse biological activities. It has been observed that pyridine has excellent inhibitory activity against carbonic anhydrase IX, a hypoxia-inducible protein, which can regulate pH in cells in the hypoxic state, stimulating cell migration, adhesion, and invasion, and thus, encouraging cancer cell survival. Its levels remain elevated in tumor tissues, making them radiotherapy and chemotherapy-resistant [22]. Hypoxia was also shown to prevent the etoposide-induced DNA damage through an HIF-1 mediated mechanism, wherein hypoxia pre-exposure was observed to cause a decrease in levels of topo-II α in a subpopulation of cells [23]. All this might make **LP-14** a better candidate for the etoposide. On the other hand, pyridine scaffolds can inhibit a variety of kinases, which are the main pivotal control points whose mutations lead to uncontrolled proliferation, thus acting as proto-oncogenes. For example, pyridines have also been shown to have an inhibitory effect on ALK/ROS1 kinases, type-II c-met (a receptor tyrosine kinase) which are pivotal enzymes in cancer progression. They have also been observed to act as potential inhibitors of mutant EGFR and HER-2 kinases [24,25,26]. The other kinases that are inhibited are PIM-1 kinase, VEGFR, CDK9, and PI3K [27,28,29]. There have also been reports of compounds containing pyridines acting as topoisomerase inhibitors.

In the case of **LP-15**, chlorine para-substitution seems to be playing an important role. Its electron donating capacity is highest among all halogens and its para position makes it a highly resonating structure, hence contributing to its stability and its ability to interact with functional groups. Halogenated compounds, especially Cl, are one of the major drug types in pharmaceutical companies, because they show diverse biological effects. Chloro-containing compounds have been found to be anti-microbial and anti-fungal; in fact, highly potent ones. There have also been studies that confirm the importance of Cl increasing the anticancer activity when substituted into the existing potent anticancer agents [30]. In a study using piperlongumine analogs, chlorinated analogs (Cl atom attached to a dihydropyridine ring) possessed more potent anticancer activity [31]. In a study involving chlorophenyl-substituted benzofuro pyridines (chlorine substituted at meta and para positions), excellent inhibitory activity against topoisomerase-II was exhibited by these compounds in many cell lines, and hence, anti-proliferative.

## 3. Materials and Methods

### 3.1. General Methods

All chemicals were obtained from Fischer Scientific Ltd, Hyderabad, India. Reactions were continuously monitored using TLC aluminum sheets with silica gel 60 F254 from Sigma-Aldrich. Melting points were determined using a Büchi B545 digital capillary melting point apparatus (BUCHI Labortechnik AG, Flawil, CH, Switzerland) and used without correction. The functional group of the carbazole derivatives was obtained with a Perkin-Elmer VERTEX 70 FT-IR spectrometer-covering field. ^1^H and ^13^C-NMR spectra were obtained in CDCl_3_ solution on a Bruker spectrometer (500 MHz). Mass spectra were collected using API 3200 LC/MS/MS system, equipped with an ESI source.

### 3.2. Chemistry: Synthesis Procedures

**Synthesis of 2-Bromo-6-(2-(1-methylpiperidin-4-ylidene)hydrazinyl)pyridine Imine (6)**

A mixture of 2-bromo-6-hydrazinylpyridine (10 g, 53.48 mmol), 1-methyl piperidine-4-one (6.04 g, 53.48 mmol, 1 eq.) in ethanol (30 mL) was heated to 50 °C for 1 h. The progress of the reaction was monitored using TLC. After completion of the reaction, the solvent was removed under reduced pressure. The obtained residue was purified by flash column chromatography on silica gel (MeOH/DCM, 0–4%) afforded desired product 2-bromo-6-(2-(1-methylpiperidin-4-ylidene) hydrazinyl) pyridine **3** (10.5 g, 70%) as dark brown color solid.

**Synthesis of 2-bromo-6-methyl-6,7,8,9-tetrahydro-5H-pyrrolo [2,3-b:4,5-c′]dipyridine (7)**

2-Bromo-6-(2-(1-methylpiperidin-4-ylidene) hydrazinyl) pyridine (5 g, 17.7 mmol) was added to 100 g of preheated (110 °C) PPA, and the reaction continued at 180 °C for 24 h. The progress of the reaction was monitored using TLC. After completion of the reaction, the mixture was cooled to room temperature and dissolved in ~600 mL water. It was basified using 4N NaOH up to pH 8 and the precipitated pale brown color solid was filtered, washed with water, and vacuum-dried. Afforded crude product 2-bromo-6-methyl-6,7,8,9-tetrahydro-5H-pyrrolo [2,3-b:4,5-c’]. Dipyridine **4** (2.3 g) as pale brown color solid was directly used in the next step without further purification. Its chemical formula is: C_11_H_12_ BrN_3_, Exact Mass: 265.02; MP: 218–222 °C; FTIR (KBr): 3167, 2936, 1877, 1543, 1406, 1352, 1092, 910, 802, 727, 689 cm^−1^. ^1^H NMR (500 MHz, DMSO-d_6_) δ 11.60 (s, 1H, NH), 7.72 (d, 1H, *J* = 8.0 Hz), 7.15 (d, 1H, *J* = 8.0 Hz), 3.54 (s, 2H), 2.79–2.77 (m, 4H), 2.44 (s, 3H). ^13^C NMR (CDCl3, 125 MHz): 148.4, 133.5, 132.8, 127.6, 118.7, 117.7, 107.3, 52.1, 51.1, 45.7, 24.2.

**Synthesis of 2-bromo-6-methyl-9-phenethyl-6,7,8,9-tetrahydro-5H-pyrrolo [2,3-b:4,5-c’]dipyridine (9)**

Cesium carbonate (7.74 g, 23.77 mmol, 3 eq.) was added to a stirred solution of 2-bromo-6-methyl-6,7,8,9-tetrahydro-5H-pyrrolo [2,3-b:4,5-c’]dipyridine (2.1 g, 7.92 mmol) in DMF (10 vol.) and stirred for 15 min at room temperature. Then, phenethyl 4-methylbenzenesulfonate **5** (4.37 g, 15.84 mmol, 2 eq.) was added at the same temperature and further stirred at 80 °C for 16 h. The progress of the reaction was monitored using TLC. After completion of the reaction, the mixture was cooled to room temperature, quenched with 50 mL water and extracted with ethyl acetate (3 × 50 mL). The combined organic layers were washed with water (100 mL), and the brine (50 mL) was dried over sodium sulfate and concentrated under reduced pressure. The obtained crude material was purified by flash column chromatography on silica using 0–4% MeOH/DCM as eluent afforded 2-bromo-6-methyl-9-phenethyl-6,7,8,9-tetrahydro-5H-pyrrolo [2,3-b:4,5-c’]dipyridine (1.9 g, 65%) as a brown solid. Its chemical formula is: C_19_H_20_BrN_3_, Exact Mass: 369.08; mp: 132–136 °C; FTIR (KBr): 3402, 2936, 2772, 2305, 1603, 1419, 1356, 1132, 1092, 966, 802, 754 cm^−1^. FTIR (KBr): 3402, 2936, 2772, 2305, 1603, 1419, 1356, 1132, 1092, 966, 802, 754 cm^−1^. ^1^H NMR (500 MHz, DMSO-d_6_) δ 7.74 (d, 1H, *J* = 10.0 Hz), 7.26–7.18 (m, 4H), 7.05 (d, 2H, *J* = 8.5 Hz), 4.31 (t, 2H, *J* = 8.5 Hz), 3.46 (s, 2H), 3.99 (t, 2H, *J* = 8.5 Hz), 2.58 (t, 2H, *J* = 7.0 Hz), 2.46 (t, 2H, *J* = 6.5 Hz), 2.56 (s, 3H). ^13^C NMR (CDCl3, 125 MHz): 147.3, 138.7, 134.4, 133.2, 129.2, 128.9, 128.5, 128.1, 127.2, 126.5, 118.6, 118.5, 117.0, 106.252.1, 51.1, 45.7, 45.6, 43.6, 36.4, 36.4, 22.6.

**General procedure for the synthesis of 6-methyl-9-phenethyl-6,7,8,9-tetrahydro-5H-pyrrolo [2,3-b:4,5-c’]dipyridine derivatives (LP-1- LP-16)**

In an oven-dried RB flask, compound **9** (1 equiv), boronic acid (1.5 equiv), NaHCO_3_ (3 equiv), Pd(dppf)Cl_2_ (0.05 equiv) in degassed dimethoxy ethane (10 vol.), and water (3 vol.) were mixed. The reaction mixture was refluxed with stirring for 2 h. The progress of the reaction was monitored using TLC. Upon completion of the reaction, the mixture was allowed to cool to room temperature and concentrated under reduced pressure. The obtained residue was diluted with ethyl acetate washed with water and brine, dried over sodium sulfate, and concentrated under reduced pressure to produce the crude.

**6-Methyl-9-phenethyl-2-phenyl-6,7,8,9-tetrahydro-5H-pyrrolo [2,3-b:4,5-c’]dipyridine (LP-1)**

Chemical formula: C_25_H_25_N_3_, exact mass: 367.20; brown solid, mp: 118–122 °C, FTIR (KBr): 3429, 2922, 2853, 1771, 1622, 104, 1333, 1177, 914, 745 cm^−1^. ^1^H NMR (500 MHz, CDCl_3_) δ 8.12 (dd, 2H, *J* = 8.0, 1.0 Hz), 7.73 (d, 1H, *J* = 8.0 Hz), 7.52 (d, 1H, *J* = 8.0 Hz), 7.48 (td, 2H, *J* = 7.5, 1.5 Hz), 7.48 (td, 1H, *J* = 7.0, 1.0 Hz), 7.28–7.25 (m, 3H), 7.22–7.19 (m, 1H), 7.13 (dd, 2H, *J* = 8.0, 1.5 Hz), 4.45 (t, 2H, *J* = 7.5 Hz), 3.64 (s, 2H), 3.15 (t, 2H, *J* = 7.5 Hz), 2.73 (t, 2H, *J* = 6.0 Hz), 2.59 (t, 2H, *J* = 6.0 Hz), 2.53 (s, 3H). ^13^C NMR (CDCl3, 125 MHz): 164.4, 162.4, 148.0, 147.7, 147.7, 143.1, 143.1, 139.1, 135.1, 129.9, 129.8, 129.0, 128.5, 126.5, 125.6, 122.1, 122.1, 117.6, 114.6, 114.4, 113.6, 112.3, 106.0,52.2, 51.4, 45.7, 43.5, 36.7, 22.9.

**2-(3-Fluorophenyl)-6-methyl-9-phenethyl-6,7,8,9-tetrahydro-5H-pyrrolo [2,3-b:4,5-c’]dipyridine (LP-2)**

Chemical formula: C_25_H_24_FN_3_, exact mass: 385.20; pale yellow solid, mp: 112–116 °C, FTIR (KBr): 3426, 2922, 2854, 1620, 1454, 1406, 1362, 912, 740 cm^−1^. ^1^H NMR (500 MHz, CDCl_3_) δ 7.88–7.86 (m, 2H), 7.73 (d, 1H, *J* = 8.0 Hz), 7.50 (d, 1H, *J* = 8.0 Hz), 7.47–7.40 (m, 1H), 7.28–7.25 (m, 2H), 7.22–7.19 (m, 1H), 7.12–7.10 (m, 2H), 7.07–7.05 (m, 1H), 4.45 (t, 2H, *J* = 7.0 Hz), 3.64 (s, 2H), 3.14 (t, 2H, *J* = 7.5 Hz), 2.73 (t, 2H, *J* = 6.0 Hz), 2.59 (t, 2H, *J* = 5.5 Hz), 2.53 (s, 3H). ^13^C NMR (CDCl3, 125 MHz): 149.3, 148.1, 140.7, 139.2, 134.5, 130.0, 128.5 (d, *J* = 10.0 Hz), 127.8, 126.8, 126.4, 125.5, 117.2, 112.4, 105.9, 52.3, 51.5, 45.7, 43.5, 36.6, 22.8. ^19^F NMR (500 MHz, CDCl_3_) δ: −113.6.

**2-(4-Fluorophenyl)-6-methyl-9-phenethyl-6,7,8,9-tetrahydro-5H-pyrrolo [2,3-b:4,5-c’]dipyridine (LP-3)**

Chemical formula: C_25_H_24_FN_3_, exact mass: 385.20; pale yellow solid, mp: 118–122 °C, FTIR (KBr): 2924, 1755, 1557, 1400, 1342, 1225, 1138, 912, 745, 648 cm^−1^. ^1^H NMR (500 MHz, CDCl_3_) δ 8.10–8.07 (m, 2H), 7.72 (d, 1H, *J* = 8.0 Hz), 7.46 (d, 1H, *J* = 8.0 Hz), 7.28–7.22 (m, 2H), 7.27–7.19 (m, 3H), 7.21–7.10 (m, 5H), 4.44 (t, 2H *J* = 7.0 Hz), 3.64 (s, 2H), 2.73 (t, 2H, *J* = 5.5 Hz), 2.59 (t, 2H, *J* = 6.0 Hz), 2.53 (s, 3H). ^13^C NMR (CDCl3, 125 MHz): 162.9 (d, *J* = 250 Hz), 148.3, 148.1, 139.1, 136.8, 136.8, 134.6, 128.9, 128.5, 128.4, 128.3, 126.5, 125.6, 117.1, 115.3 (d, *J* = 21.5 Hz), 112.0, 105.9, 52.3, 51.4, 45.7, 43.5, 36.6, 22.8. ^19^F NMR (500 MHz, CDCl_3_) δ: −115.2.

**4-(6-Methyl-9-phenethyl-6,7,8,9-tetrahydro-5H-pyrrolo [2,3-b:4,5-c’]dipyridin-2-yl)benzonitrile (LP-4)**

Chemical formula: C_26_H_24_N_4_, exact mass: 392.20; pale yellow solid, mp: 124–128 °C, FTIR (KBr): 2924, 2852, 2223, 1778, 1377, 1330, 1180, 913, 746 cm^−1^. ^1^H NMR (500 MHz, CDCl_3_) δ 8.23 (d, 2H, *J* = 8.5 Hz), 7.76–7.74 (m, 3H), 7.55 (d, 1H, *J* = 8.0 Hz), 7.27–7.19 (m, 3H), 7.09 (dd, 2H, *J* = 7.8, 1.5 Hz), 4.45 (t, 2H *J* = 7.5 Hz), 3.64 (s, 2H), 3.14 (t, 2H, *J* = 7.5 Hz), 2.73 (t, 2H, *J* = 5.5 Hz), 2.60 (t, 2H, *J* = 5.5 Hz), 2.53 (s, 3H). ^13^C NMR (CDCl3, 125 MHz): 148.1, 146.6, 144.9, 138.9, 136.0, 132.4, 128.9, 128.5, 127.0, 126.5, 125.6, 119.3, 118.2, 112.7, 110.9, 106.2, 52.2, 51.3, 45.7, 43.6, 36.6, 22.9.

**6-Methyl-2-(naphthalen-1-yl)-9-phenethyl-6,7,8,9-tetrahydro-5H-pyrrolo [2,3-b:4,5-c’]dipyridine (LP-5)**

Chemical formula: C_29_H_27_N_3_, exact mass: 417.22; brown solid, mp: 130–134 °C, FTIR (KBr): 3028, 2922, 2851, 1942, 1004, 1373, 912, 742, 694 cm^−1^. ^1^H NMR (500 MHz, CDCl_3_) δ 8.34 (d, 1H, *J* = 8.5 Hz), 7.91 (td, 2H, *J* = 10.0, 1.0 Hz), 7.81 (d, 1H, *J* = 8.0 Hz), 7.68 (dd, 1H, *J* = 7.0, 1.0 Hz), 7.57 (td, 1H, *J* = 8.0, 1.0 Hz), 7.49 (td, 1H, *J* = 8.5, 1.5 Hz), 7.44 (td, 1H, *J* = 8.5, 1.5 Hz), 7.34 (d, 1H, *J* = 8.0 Hz), 7.24–7.18 (m, 3H), 7.08 (dd, 2H, *J* = 7.5, 1.0 Hz), 4.43 (t, 2H, *J* = 7.5 Hz), 3.70 (s, 2H), 3.14 (t, 2H, *J* = 7.5 Hz), 2.76 (t, 2H, *J* = 6.0 Hz), 2.61 (t, 2H, *J* = 6.0 Hz), 2.61 (s, 3H). ^13^C NMR (CDCl3, 125 MHz): 151.1, 147.8, 139.7, 139.1, 134.5, 134.1, 131.7, 129.0, 128.5, 128.2, 128.1, 127.8, 126.5, 126.4, 125.9, 125.6, 125.4, 125.3, 117.1, 116.9, 105.9, 52.3, 51.5, 45.7, 43.5, 36.9, 22.8.

**2-(2,6-Difluorophenyl)-6-methyl-9-phenethyl-6,7,8,9-tetrahydro-5H-pyrrolo [2,3-b:4,5-c’]dipyridine (LP-6)**

Chemical formula: C_25_H_23_F_2_N_3_, exact mass: 403.19; colorless liquid (thick mass)**,** FTIR (KBr): 2925, 2784, 1624, 1461, 1379, 1229, 1136, 999, 913, 744, 699 cm^−1^. ^1^H NMR (500 MHz, CDCl_3_) δ 7.75 (d, 1H, *J* = 8.0 Hz), 7.35–7.29 (m, 1H), 7.26–7.17 (m, 4H), 7.08 (d, 2H, *J* = 7.0 Hz), 7.04 (t, 2H, *J* = 5.0 Hz), 4.40 (t, 2H, *J* = 7.5 Hz), 3.64 (s, 2H), 3.12 (t, 2H, *J* = 7.5 Hz), 2.70 (t, 2H, *J* = 6.0 Hz), 2.54 (t, 2H, *J* = 6.0 Hz), 2.51 (s, 3H). ^13^C NMR (CDCl3, 125 MHz): 160.8 (d, *J* = 249.0, 7.0 Hz), 147.7, 140.5, 139.1, 135.1, 129.1, 129.0, 129.0, 128.4, 126.4, 124.9, 119.4 (d, *J* = 18 Hz), 117.5 (d, *J* = 15.0 Hz), 111.6 (d, *J* = 20.0, 6.0 Hz), 105.8, 52.2, 51.3, 45.6, 43.6, 36.3, 22.7. ^19^F NMR (500 MHz, CDCl_3_) δ: −114.3, −134.3.

**2-(2-Fluoro-6-methoxyphenyl)-6-methyl-9-phenethyl-6,7,8,9-tetrahydro-5H-pyrrolo [2,3-b:4,5-c’]dipyridine (LP-7)**

Chemical formula: C_26_H_26_FN_3_O, exact mass: 415.21; pale yellow solid, mp: 144–148 °C, FTIR (KBr): 2924, 2184, 1746, 1666, 1581, 1447, 1378, 1197, 1080, 913, 744, 654 cm^−1^. ^1^H NMR (500 MHz, CDCl_3_) δ 7.73 (d, 1H, *J* = 8.0 Hz), 7.33–7.28 (m, 1H), 7.26–7.14 (m, 4H), 7.06 (d, 2H, *J* = 7.0 Hz), 6.84–6.80 (m, 2H), 4.39 (t, 2H, *J* = 7.5 Hz), 3.79 (s, 3H, OCH_3_), 3.36 (s, 2H), 3.12 (t, 2H, *J* = 7.5 Hz), 3.68 (t, 2H, *J* = 6.0 Hz), 2.50 (s, 5H). ^13^C NMR (CDCl3, 125 MHz): 161.2 (d, *J* = 245.0 Hz), 158.7, 158.6, 147.8, 143.1, 139.3, 134.4, 129.2, 129.1, 129.0, 128.4, 126.3, 124.7, 119.8 (d, *J* = 17.0 Hz), 117.7, 117.1, 108.5(d, *J* = 23.0 Hz), 107.0, 105.7, 56.2, 52.3, 51.4, 45.7, 43.6, 36.3, 22.6. ^19^F NMR (500 MHz, CDCl_3_) δ: −115.2

**3-(6-Methyl-9-phenethyl-6,7,8,9-tetrahydro-5H-pyrrolo [2,3-b:4,5-c’]dipyridin-2-yl)benzonitrile (LP-8)**

Chemical formula: C_26_H_24_N_4_, exact mass: 392.20; pale yellow solid, mp: 104–108 °C, FTIR (KBr): 2852, 2227, 1742, 1665, 1453, 1379, 1356, 1254, 1136, 912, 744, 700 cm^−1^. ^1^H NMR (500 MHz, CDCl_3_) δ 8.42 (t, 1H, *J* = 1.5 Hz), 8.30 (dt, 1H, *J* = 8.0, 1.5 Hz), 7.75 (d, 1H, *J* = 8.0 Hz), 7.63 (dt, 1H, *J* = 8.0, 1.5 Hz), 7.55 (t, 1H, *J* = 7.5 Hz), 7.49 (d, 1H, *J* = 8.0 Hz), 7.27–7.25 (m, 2H), 7.20 (td, 1H, *J* = 8.25, 1.0 Hz), 7.08 (dd, 2H, *J* = 8.25, 1.0 Hz), 4.46 (t, 2H *J* = 7.5 Hz), 3.64 (s, 2H), 3.13 (t, 2H, *J* = 7.5 Hz), 2.73 (t, 2H, *J* = 5.5 Hz), 2.59 (t, 2H, *J* = 5.5 Hz), 2.53 (s, 3H). ^13^C NMR (CDCl3, 125 MHz): 148.1, 146.4, 141.8, 139.0, 135.7, 130.9, 130.7, 130.5, 129.2, 128.9, 128.5, 126.5, 125.7, 119.3, 118.0, 112.7, 112.1, 106.2, 52.2, 51.4, 45.7, 43.6, 36.7, 22.9.

**2-(3-Chloro-2-methylphenyl)-6-methyl-9-phenethyl-6,7,8,9-tetrahydro-5H-pyrrolo [2,3-b:4,5-c’]dipyridine (LP-9)**

Chemical formula: C_26_H_26_ClN_3_, exact Mass: 415.18; brown solid, mp: 89–91 °C, FTIR (KBr): 2923, 2852, 2184, 1745, 1669, 1584, 1378, 1255, 1132, 1006, 969, 913, 745, 694 cm^−1^. ^1^H NMR (500 MHz, CDCl_3_) δ 7.73 (d, 1H, *J* = 8.0 Hz), 7.40 (dd, 1H, *J* = 7.5, 1.0 Hz), 7.36 (dd, 1H, *J* = 7.5, 1.0 Hz), 7.26–7.19 (m, 5H), 7.10 (d, 1H, *J* = 7.5 Hz), 7.06 (d, 2H, *J* = 7.0 Hz), 4.40 (t, 2H, *J* = 7.5 Hz), 3.65 (s, 2H), 3.09 (t, 2H, *J* = 7.5 Hz), 2.73 (t, 2H, *J* = 6.0 Hz), 2.58 (t, 2H, *J* = 5.5 Hz), 2.53 (s, 3H), 2.44 (s, 3H). ^13^C NMR (CDCl3, 125 MHz): 151.3, 147.4, 143.7, 139.0, 135.5, 134.6, 134.5, 128.9, 128.8, 128.5, 126.5, 126.3, 125.2, 116.8, 116.1, 105.8, 52.2, 51.4, 45.7, 43.5, 36.8, 22.7, 17.8.

**6-Methyl-9-phenethyl-2-(2-(trifluoromethyl)phenyl)-6,7,8,9-tetrahydro-5H-pyrrolo [2,3-b:4,5-c’]dipyridine (LP-10)**

Chemical formula: C_26_H_24_F_3_N_3_, exact mass: 435.19; brown solid, mp: 72–76 °C, FTIR (KBr): 2925, 2853, 2147, 1743, 1593, 1452, 1380, 1098, 1032, 913, 746, 654 cm^−1^. ^1^H NMR (500 MHz, CDCl_3_) δ 7.80 (d, 1H, *J* = 7.5 Hz), 7.74 (d, 1H, *J* = 8.0 Hz), 7.63–7.57 (m, 2H), 7.50 (t, 1H, *J* = 7.0 Hz), 7.26–7.22 (m, 2H), 7.21–7.16 (m, 4H), 7.06 (dd, 2H, *J* = 8.0, 1.0 Hz), 4.38 (t, 2H, *J* = 7.0 Hz), 3.65 (s, 2H), 3.09 (t, 2H, *J* = 7.0 Hz), 2.71 (t, 2H, *J* = 6.0 Hz), 2.55 (t, 2H, *J* = 6.0 Hz), 2.52 (s, 3H). ^13^C NMR (CDCl3, 125 MHz): 149.7, 147.3, 141.5, 139.1, 134.9, 132.1, 131.7, 131.3, 129.0, 128.8, 128.7, 128.5, 128.4, 128.2, 127.7, 127.5, 126.7, 126.6, 126.3, 125.5, 125.0, 123.3, 117.2, 115.5, 105.7, 52.2, 51.4, 45.6, 43.6, 36.4, 22.7. ^19^F NMR (500 MHz, CDCl_3_) δ: −56.6.

**2-(2-Fluorophenyl)-6-methyl-9-phenethyl-6,7,8,9-tetrahydro-5H-pyrrolo [2,3-b:4,5-c’]dipyridine: (LP-11)**

Chemical formula: C_25_H_24_FN_3_, exact mass: 385.20; brown solid, mp: 75–79 °C, FTIR (KBr): 2922, 2853, 2260, 1587, 1452, 1377, 1315, 914, 744, 659 cm^−1^. ^1^H NMR (500 MHz, CDCl_3_) δ 8.10 (td, 1H, *J* = 7.5, 1.5 Hz), 7.73 (d, 1H, *J* = 8.0 Hz), 7.58 (dd, 1H, *J* = 8.0, 2.0 Hz), 7.34–7.11 (m, 1H), 7.29–7.24 (m, 2H), 7.23–7.14 (m, 2H), 7.10 (dd, 2H, *J* = 7.5, 1.5 Hz), 4.44 (t, 2H, *J* = 7.5 Hz), 3.65 (s, 2H), 3.14 (t, 2H, *J* = 7.0 Hz), 2.73 (t, 2H, *J* = 5.5 Hz), 2.59 (t, 2H, *J* = 5.5 Hz), 2.53 (s, 3H). ^13^C NMR (CDCl3, 125 MHz): 160.6 (d, *J* = 248.0 Hz), 159.6, 148.0, 145.0, 139.1, 134.8, 131.2, 131.1, 129.2, 129.1, 129.0, 128.8, 128.7, 128.4, 126.4, 125.1, 124.2 (d, *J* = 3.4 Hz), 117.3, 116.3 (d, *J* = 8.0 Hz), 116.1, 105.9, 52.2, 51.4, 45.7, 43.5, 36.6, 22.8. ^19^F NMR (500 MHz, CDCl_3_) δ: −116.4.

**2-(3-Fluoro-4-methoxyphenyl)-6-methyl-9-phenethyl-6,7,8,9-tetrahydro-5H-pyrrolo [2,3-b:4,5-c’]dipyridine (LP-12)**

Chemical formula: C_26_H_26_FN_3_O, exact mass: 411.21; pale yellow solid, mp: 72–77 °C, FTIR (KBr): 2922, 2365, 1449, 1381, 1283, 1182, 910, 775, 740, 659 cm^−1^. ^1^H NMR (500 MHz, CDCl_3_) δ 7.93 (dd, 1H, *J* = 13.0, 2.0 Hz), 7.82 (dd, 1H, *J* = 8.5, 2.0 Hz), 7.70 (d, 1H, *J* = 8.0 Hz), 7.28–7.25 (m, 2H), 7.23 (t, 1H, *J* = 7.5 Hz), 7.11 (dd, 2H, *J* = 7.5, 1.5 Hz), 7.05 (t, 1H, *J* = 7.75 Hz), 4.44 (t, 2H, *J* = 7.5 Hz), 3.96 (s, 3H), 3.63 (s, 2H), 3.13 (t, 2H, *J* = 7.5 Hz), 2.73 (t, 2H, *J* = 5.5 Hz), 2.59 (t, 2H, *J* = 5.5 Hz), 2.52 (s, 3H). ^13^C NMR (CDCl3, 125 MHz): 152.7 (d, *J* = 242.6 Hz), 147.8 (d, *J* = 29.6 Hz), 147.5 (d, *J* = 10.9 Hz), 139.1, 134.5, 134.3, 134.2, 129.0, 128.5, 126.4, 125.6, 122.2, 122.1, 117.1, 114.4 (d, *J* = 19.5 Hz), 113.3, 111.7, 105.9, 56.4, 52.3, 51.4, 45.7, 43.5, 36.6, 22.8. ^19^F NMR (500 MHz, CDCl_3_) δ: −135.6.

**6-Methyl-9-phenethyl-2-(thiophen-3-yl)-6,7,8,9-tetrahydro-5H-pyrrolo [2,3-b:4,5-c’]dipyridine (LP-13)**

Chemical formula: C_23_H_23_N_3_S, exact mass: 373.16; pale yellow solid, mp: 112–116 °C, FTIR (KBr): 2886, 2360, 1726, 1575, 1494, 1379, 1319, 912, 773, 742, 682, 630 cm^−1^. ^1^H NMR (500 MHz, CDCl_3_) δ 7.88 (dd, 1H, *J* = 3.5, 1.5 Hz), 7.79 (dd, 1H, *J* = 6.5, 1.5 Hz), 7.68 (d, 1H, *J* = 10.5 Hz), 7.52–7.38 (m, 2H), 7.29–7.18 (m, 1H), 7.12 (d, 2H, *J* = 9.5 Hz), 4.42 (t, 2H, *J* = 9.0 Hz), 3.64 (s, 2H), 3.13 (t, 2H, *J* = 9.0 Hz), 2.74 (s, 2H), 2.59 (s, 2H), 2.53 (s, 3H). ^13^C NMR (CDCl3, 125 MHz): 147.8, 145.8, 143.7, 139.2, 134.2, 128.9, 128.5, 126.6, 126.4, 125.7, 125.8, 121.5, 117.0, 112.4, 105.8, 52.2, 51.4, 45.6, 43.5, 36.6, 22.7.

**6-Methyl-9-phenethyl-2-(pyridin-3-yl)-6,7,8,9-tetrahydro-5H-pyrrolo [2,3-b:4,5-c’]dipyridine (LP-14)**

Chemical formula: C_24_H_24_N_4_, exact mass: 368.20; white solid, mp: 98–102 °C, FTIR (KBr): 2929, 2355, 1458, 1378, 1286, 1172, 914, 778, 747, 650 cm^−1^. ^1^H NMR (500 MHz, CDCl_3_) δ 9.35 (d, 1H, *J* = 2.0 Hz), 8.59 (dd, 1H, *J* = 5.0, 1.5 Hz), 8.39 (dt, 1H, *J* = 8.5, 1.5 Hz), 7.70 (d, 1H, *J* = 8.5 Hz), 7.52 (d, 1H, *J* = 8.0 Hz), 7.41–7.38 (m, 1H), 7.27–7.20 (m, 3H), 7.11 (dd, 2H, *J* = 8.0, 1.0 Hz), 4.45 (t, 2H, *J* = 7.0 Hz), 3.65 (s, 2H), 3.14 (t, 2H, *J* = 7.0 Hz), 2.74 (t, 2H, *J* = 5.5 Hz), 2.61 (t, 2H, *J* = 5.5 Hz), 2.53 (s, 3H). ^13^C NMR (CDCl3, 125 MHz): 148.7, 148.4, 148.2, 146.3, 139.0, 136.0, 135.2, 133.9, 128.9, 128.5, 126.5, 125.7, 123.4, 117.7, 112.2, 106.1, 52.2, 51.4, 45.8, 43.6, 36.6, 22.9.

**2-(4-Chlorophenyl)-6-methyl-9-phenethyl-6,7,8,9-tetrahydro-5H-pyrrolo [2,3-b:4,5-c’]dipyridine (LP-15)**

Chemical formula: C_25_H_24_ClN_3_, exact mass: 401.17; brown solid, mp: 128–132 °C, FTIR (KBr): 2922, 2853, 2360, 1732, 150, 1363, 1327, 1089, 914, 808, 742, 657 cm^−1^. ^1^H NMR (500 MHz, CDCl_3_) δ 8.06 (dd, 2H, *J* = 7.5, 2.0 Hz), 7.72 (d, 1H, *J* = 8.0 Hz), 7.48 (d, 1H, *J* = 8.5 Hz), 7.44 (dd, 2H, *J* = 7.5, 2.0 Hz), 7.28–7.20 (m, 3H), 7.10 (d, 2H, *J* = 7.0 Hz), 4.44 (t, 2H, *J* = 7.5 Hz), 3.64 (s, 2H), 3.14 (t, 2H, *J* = 7.5 Hz), 2.73 (t, 2H, *J* = 6.0 Hz), 2.59 (t, 2H, *J* = 5.5 Hz), 2.53 (s, 3H). ^13^C NMR (CDCl3, 125 MHz): 148.0, 148.0, 139.1, 134.8, 133.7, 129.0, 128.6, 128.5, 128.0, 126.5, 125.6, 117.4, 112.1, 105.9, 52.2, 51.4, 45.7, 43.5, 36.6, 22.8.

**2-(3-Chlorophenyl)-6-methyl-9-phenethyl-6,7,8,9-tetrahydro-5H-pyrrolo [2,3-b:4,5-c’]dipyridine (LP-16)**

Chemical formula: C_25_H_24_ClN_3_, exact mass: 401.17; pale yellow solid, mp: 126–130 °C, FTIR (KBr): 2853, 2783, 1739, 1600, 1558, 1456, 1355, 1182, 1134, 968, 756, 698 cm^−1^. ^1^H NMR (500 MHz, CDCl_3_) δ 8.13 (t, 1H, *J* = 2.0 Hz), 7.97 (td, 1H, *J* = 8.0, 1.0 Hz), 7.73 (d, 1H, *J* = 8.0 Hz), 7.49 (d, 1H, *J* = 8.0 Hz), 7.39 (t, 1H, *J* = 7.5 Hz), 7.34–7.20 (m, 3H), 7.10 (d, 2H, *J* = 7.0 Hz), 4.45 (t, 2H, *J* = 7.5 Hz), 3.64 (s, 2H), 3.14 (t, 2H, *J* = 7.5 Hz), 2.74 (t, 2H, *J* = 5.5 Hz), 2.59 (t, 2H, *J* = 5.5 Hz), 2.53 (s, 3H). ^13^C NMR (CDCl3, 125 MHz): 148.0, 147.6, 142.5, 139.1, 135.0, 134.6, 129.7, 129.0, 128.5, 127.7, 126.9, 126.5, 125.6, 124.7, 117.6, 112.3, 105.9.

### 3.3. MTT Assay

Individual wells of a 96-well tissue culture microtiter plate were inoculated with 100 µL of complete medium containing 1 × 10^4^ cells. The plates were incubated at 37 °C in a humidified 5% CO_2_ incubator for 18 h prior to the experiment. After medium removal, 100 µL of fresh medium containing the test compounds and etoposide at different concentrations, such as 0.5, 1, and 2 µM, were added to each well and incubated at 37 °C for 24 h. Then, the medium was discarded and replaced with 10 µL MTT dye. Plates were incubated at 37 °C for 2 h. The resulting formazan crystals were solubilized in 100 µL extraction buffer. The optical density (O.D) was read at 570 nm with a microplate reader (Multi-mode Varioskan Instrument-Thermo Scientific, Hyderabad, India). The percentage of DMSO in the medium never exceeded 0.25%.

### 3.4. Molecular Docking Studies with Human Topoisomerase-II beta in Complex with DNA and Etoposide (PDB ID: 3QX3)

Molecular docking studies were performed on the target compounds with Human topoisomerase-II beta in complex with DNA and etoposide (PDB ID: 3QX3)^14^. The docking was performed using AutoDock v4.0 with default settings. The macromolecule preparation was carried out by removing water molecules, ligands, ions, or other heteroatoms. Appropriate atomic charges and atom types were assigned to the protein structure. Missing hydrogen atoms were added to the protein structure. Energy minimization was performed on the protein structure to remove steric clashes or unfavorable interactions. The binding site or active site on the protein where the ligand is expected to bind was identified and defined. Etoposide is a type-II topoisomerase inhibitor, which traps the enzyme in a covalent complex with DNA. Specifically, etoposide forms a ternary complex with the enzyme and DNA by binding to a pocket in the DNA-binding site of topo II β, which is adjacent to the cleavage site on the DNA molecule. This stabilizes the enzyme–DNA covalent complex, preventing the enzyme from releasing the DNA and resulting in DNA damage and cell death. Redocking of etoposide has shown similar interactions with the co-crystallized ligand, with an RMSD value of 0.84 Å. The dock score for etoposide was found to be −10.68 kcal/mol. The binding of etoposide to topo II α is mediated by a number of interactions, including hydrogen bonds and van der Waals contacts. The oxygen atoms in the etoposide ring form hydrogen bonds with the side chains of Asp479, Gly478, and Gln778 in topo II β. Etoposide forms H-bond interactions with DNA nucleotides DC8, DG13, DG10, DT9, DC8, and DT9. It forms hydrophobic interactions with the Arg503 residue and with nucleotides DG13 and DA12, contributing to the stabilization of the drug–enzyme complex (Figure 2). Overall, the binding of etoposide to topo II β is highly specific and involves a complex network of interactions between the drug and the enzyme, as illustrated in Figure 2. The newly synthesized hybrids showed similar binding patterns to topo II β. The synthesized hybrids LP-1 and LP-16 exhibited the same interactions with the co-crystallized ligand topo II (Figure 2). 

## 4. Conclusions

In summary, we have described the concise synthesis and systematic structure–activity relationship of a novel small molecule library of nitrogen-incorporated **γ**-Carboline derivatives of latrepirdine. The designed novel **γ**-Carboline derivatives were synthesized using the Suzuki coupling reaction to identify leads for testing the anticancer activities. These compounds were tested for their anticancer activity against cell lines, particularly human cancer cell lines MCF7 (breast), A549 (lung), SiHa (cervix), and Colo-205 (colon). Most of the γ-Carboline derivatives showed potent inhibitory activity in four cancer cell lines, according to in vitro anticancer activity screening. Interestingly, the chloro-substituted compound **LP-15** showed decent activity against four anticancer cell lines ranging from 1.2 to 1.5 µM. In addition, the compounds furyl and pyridyl substitutions also showed potent anticancer activity against all four cell lines, whereas we found promising activity against the human cervix cancer cell lines (SiHa) up to 1 µM. Additionally, the active compounds (**LP-14**, **LP-15**) along with etoposide were examined through molecular docking studies on human topoisomerase II beta in complex with DNA and etoposide (PDB ID: 3QX3). The docking studies results showed that the latrepirdine-derivative chloro-substitution on phenyl (**LP-15**) was strongly bound with the receptor amino acid residues Glu477. Finally, all the results conclude that the anti-histamine **γ**-Carboline scaffold is an ideal choice for elaborate anticancer drug discovery.

## Figures and Tables

**Table 1:** In vitro cytotoxic activity of compounds (**LP-1** to **LP-16**) in (IC_50_ in µM) ^a^.

S NO	Sample Code	STRUCTURE	^bc^ SiHa	^d^ A549	^e^ MCF-7	^f^ Colo-205
1	**LP-1**	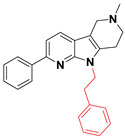	3.47 ± 2.17	5.16 ± 1.17	3.91 ± 1.49	8.13 ± 1.70
2	**LP-2**	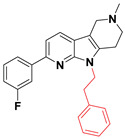	ND	4.28 ± 1.71	7.33 ± 4.52	3.62 ± 2.04
3	**LP-3**	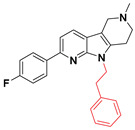	4.82 ± 0.94	6.33 ± 1.65	ND	ND
4	**LP-4**	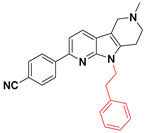	ND	10.6 ± 2.27	11.3 ± 2.93	3.98 ± 0.43
5	**LP-5**	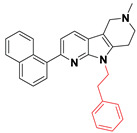	4.13 ± 3.21	5.71 ± 2.33	6.14 ± 1.75	ND
6	**LP-6**	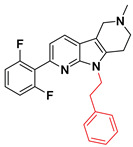	2.86 ± 1.67	2.52 ± 1.98	2.09 ± 1.83	2.49 ± 1.93
7	**LP-7**	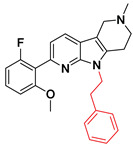	1.34 ± 0.095	2.11 ± 0.96	1.98 ± 0. 81	1.62 ± 0.85
8	**LP-8**	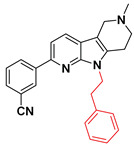	ND	ND	12.7 ± 2.38	ND
9	**LP-9**	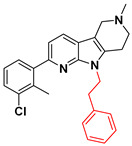	ND	10.8 ± 2.39	5.93 ± 1.61	ND
10	**LP-10**	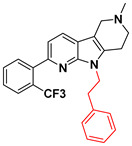	2.01 ± 1.58	1.77 ± 0.43	1.81 ± 0.56	2.13 ± 0.97
11	**LP-11**	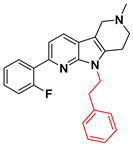	10.3 ± 1.37	6.19 ± 1.61	7.10 ± 2.43	ND
12	**LP-12**	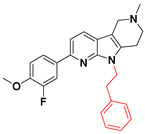	9.37 ± 2.26	17.4 ± 352	13.6 ± 4.14	8.11 ± 3.38
13	**LP-13**	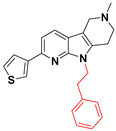	1.10 ± 0.36	2.03 ± 0.89	2.17 ± 0. 59	3.44 ± 0.96
14	**LP-14**	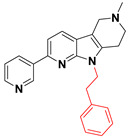	1.85 ± 0.92	1.68 ± 0.87	1.72 ± 0.88	1.95 ± 0.67
15	**LP-15**	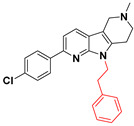	1.24 ± 0.58	1.38 ± 0.81	1.27 ± 0.64	1.53 ± 0.92
16	**LP-16**	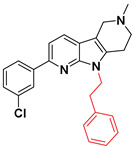	9.56 ± 2.19	4.64 ± 1.11	6.82 ± 2.05	ND
17	**Etoposide**		3.11 ± 0.11	3.08 ± 0.135	2.11 ± 0.024	0.14 ± 0.017

^a^ Each data represents mean ± SD values. From three different experiments performed in triplicates, ^bc^ SiHa: human Cervix cancer cell line, ^d^ A549: human lung cancer cell line, ^e^ MCF-7: human breast cancer cell line, ^f^ Colo-205: human colon cancer cell line. ND = Not determine.

**Table 2:** Docking score of synthesized hybrids against human topoisomerase II beta in complex with DNA and etoposide (PDB ID: 3QX3).

SNO	Compounds	Dock Score (Kcal/mol)	H-Bond Interactions	Hydrophobic Interactions
1	**LP-1**	−9.42	DC8, DT9	DG7, DT9DC11, DG13,
2	**LP-2**	−9.11	DC8, DT9	DC8, DT9, DG10, DC11
3	**LP-3**	−8.47	DC8, DA12	DG10, DC11, DG13, Ala521,
4	**LP-4**	−8.56	Arg503, DT9	DC8, DT9, DG10, DC11
5	**LP-5**	−8.78	DC8	DT9, DA12,
6	**LP-6**	−9.25	DC8, DG10, DC11,	DC8, DG10, DC11, Arg503
7	**LP-7**	−8.21	DA12, DC8, DT9, Arg503	DC8, DT9, DG13, Ala779, Arg503
8	**LP-8**	−8.69	DC8, DA12, DG13,	DC8, DA12, DG13, Ala521
9	**LP-9**	−8.99	DC8, DT9,	DC8, DT9, DA12, DG13
10	**LP-10**	−9.18	DG10, Asp479, Arg503,	DC8, DT9, DA12, DG13, Arg503,
11	**LP-11**	−9.04	DA12, DG13, Gly478, DT9, DG10	DG13, DA12, Arg503, Met782
12	**LP-12**	−7.69	DA12	DT9, DA12, DG13
13	**LP-13**	−8.92	DT9	DT9, DA12
14	**LP-14**	−8.89	DC8	DT9, DA12, DG13, DT9, DA12, DC8, Arg503
15	**LP-15**	−9.65	Glu477, DC8	DT9, DA12, DG13, DA6, DG7, DC14, DC8 and DT15
16	**LP-16**	−9.09	--	DG7, DT9, DA12, DG13, DA6
17	**Etoposide**	−10.68	Asp479, Gln778, DC8, Gly478, DG10, DT9,	DG13, Met782, DA12, DG13, Arg503

## Data Availability

Not applicable.

## Supplementary Materials

The following supporting information can be downloaded at: https://www.mdpi.com/article/10.3390/molecules28134965/s1.

## Sample Availability

Not available.
