# Supplementary material for: Design, Synthesis, Molecular Docking Study and Biological Evaluation of Novel γ-Carboline Derivatives of Latrepirdine (Dimebon) as Potent Anticancer Agents
Source: Molecules. 2023 Jun 24;28(13):4965. doi: 10.3390/molecules28134965 (PMC10343707; doi:10.3390/molecules28134965)
Supplement: Supplementary file 1 [file molecules-28-04965-s001.zip › molecules-2441663-supplementary.pdf]

Design, Synthesis, Molecular Docking Study and Biological Evaluation of Novel γ-Carboline Derivatives of Latrepirdine (Dimebon) as a Potent Anticancer Agents

Ramakrishna Voggu[a, b], Arundhati Karmakar,[c] Venkat Swamy Puli[a], V. Surendra Babu Damerla[a], Padma Mogili[b], P. Amaladass[d], Sridhar Chidara[a,] *, Kalyan Kumar Pasunooti[e,f,] *, Sarika Gupta[c,] *

[a] *Department of Medicinal Chemistry, Aragen Life Sciences Pvt. Ltd. (Formerly known as GVK Biosciences Pvt. Ltd.), IDA, Nacharam, Hyderabad, Telangana, 500076, India*
[b] *Department of Engineering Chemistry, Andhra University, Visakhapatnam, A.P., 530003, India*
[c] *Molecular Science Laboratory, National Institute of Immunology, New Delhi 110067, India*
[d] *Department of Chemistry, Madanapalle Institute of Technology & Science, Madanapalle, Andhra Pradesh, 517325, India.*
[e] *ProSAM Bioscience Pvt. Ltd., Hyderabad, Telangana, 500049, India.*
[f] *Department of Pharmacology and Molecular Sciences, Johns Hopkins School of Medicine, Baltimore, Maryland 21205, United States*

* Corresponding author.
E-mail address: sarika@nii.ac.in (S. Gupta) and *kpasuno1@jhmi.edu* (K K Pasunooti)

List of Contents

1) ^{1}H NMR, ^{13}C NMR, ^{19}FNMR, Mass spectra and Elemental composition analysis reports of obtained compounds (6, 7, 9, LP-1 to LP-16).

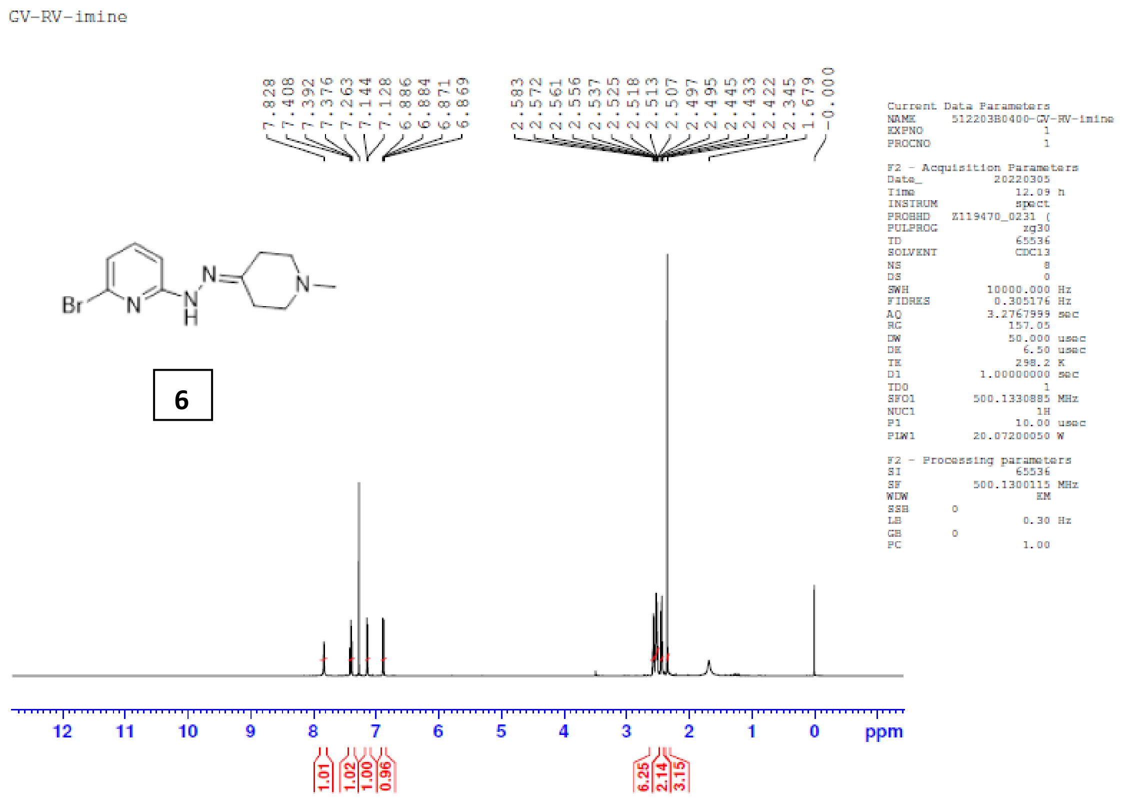

^{1}HNMR spectrum of 2-bromo-6-(2-(1-methylpiperidin-4-ylidene)hydrazinyl)pyridine (6) in $CDCl_3$ (500 MHz)

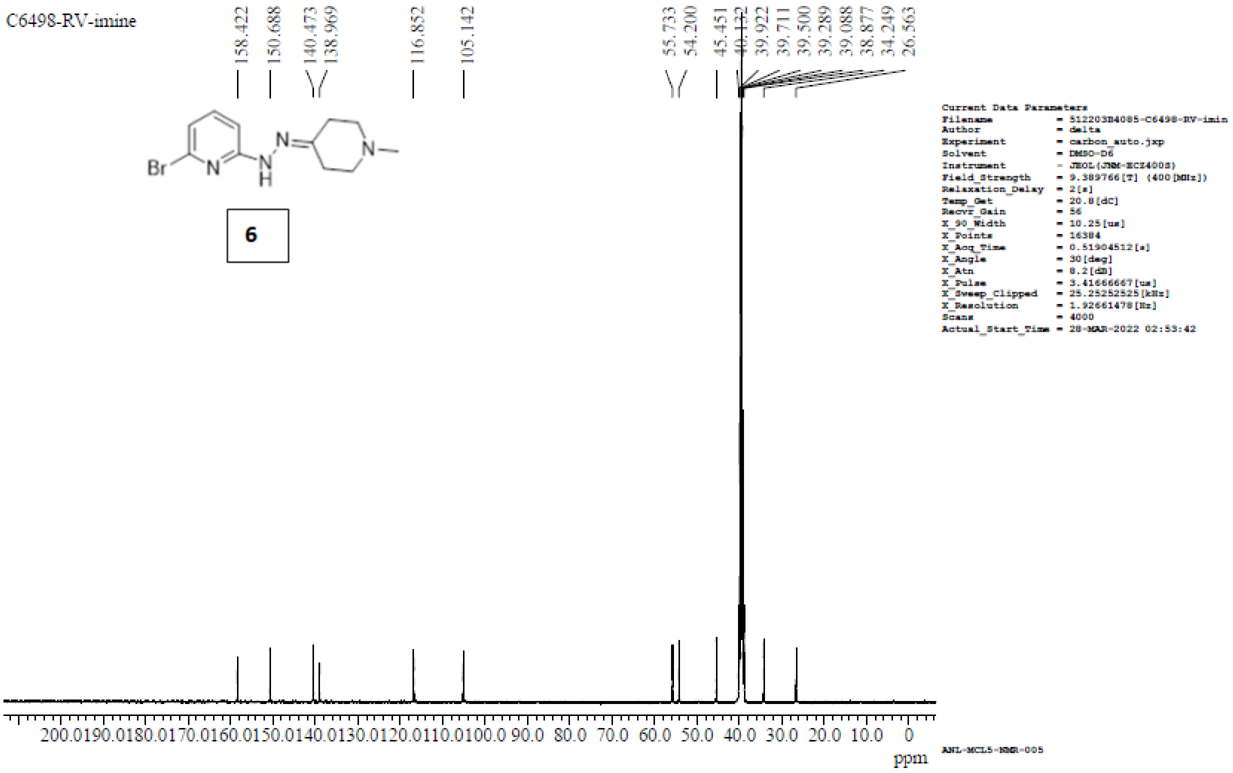

^{13}CNMR spectrum of 2-bromo-6-(2-(1-methylpiperidin-4-ylidene)hydrazinyl)pyridine (6) in DMSO (125 MHz)

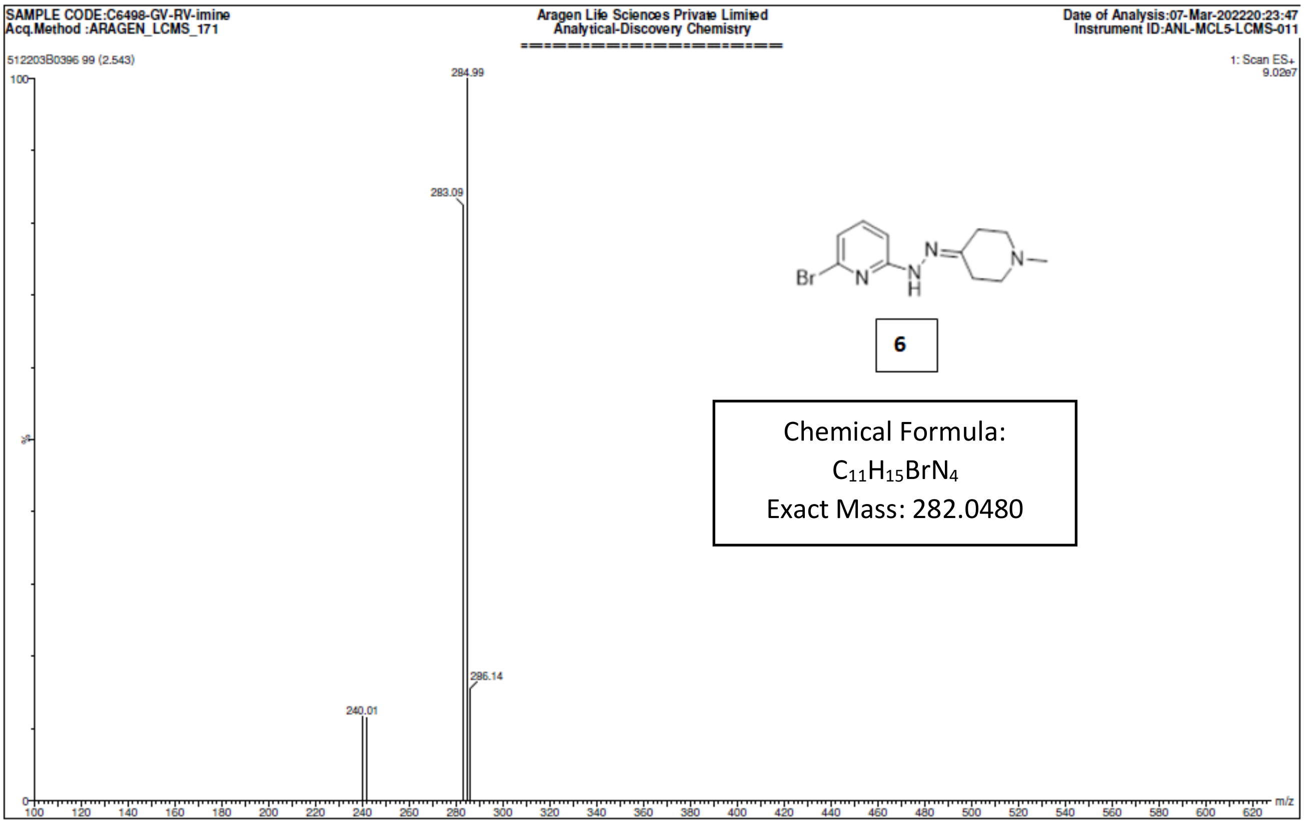

Mass spectrum of 2-bromo-6-(2-(1-methylpiperidin-4-ylidene)hydrazinyl)pyridine (6)

Elemental Composition Report **Page 1**

Single Mass Analysis
Tolerance = 820.0 PPM / DBE: min = -1.5, max = 50.0
Element prediction: Off
Number of isotope peaks used for i-FIT = 3

Monoisotopic Mass, Even Electron Ions
9 formula(e) evaluated with 1 results within limits (up to 50 best isotopic matches for each mass)
Elements Used:
C: 0-11 H: 0-16 N: 0-4 Br: 0-1

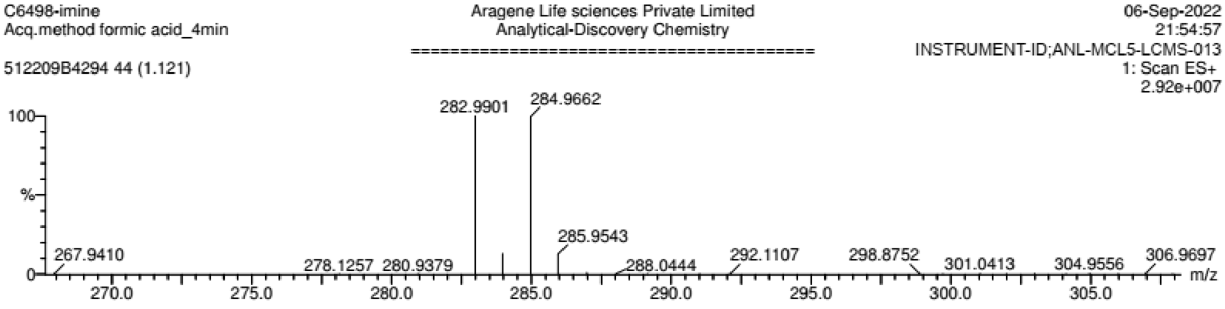

Minimum:				-1.5				
Maximum:		5.0	820.0	50.0				
Mass	Calc. Mass	mDa	PPM	DBE	i-FIT	Norm	Conf(%)	Formula
283.0528	283.0558	-3.0	-10.6	5.5	44.4	n/a	n/a	C11 H16 N4 Br

Elemental composition report of 2-bromo-6-(2-(1-methylpiperidin-4-ylidene)hydrazinyl)pyridine (6)

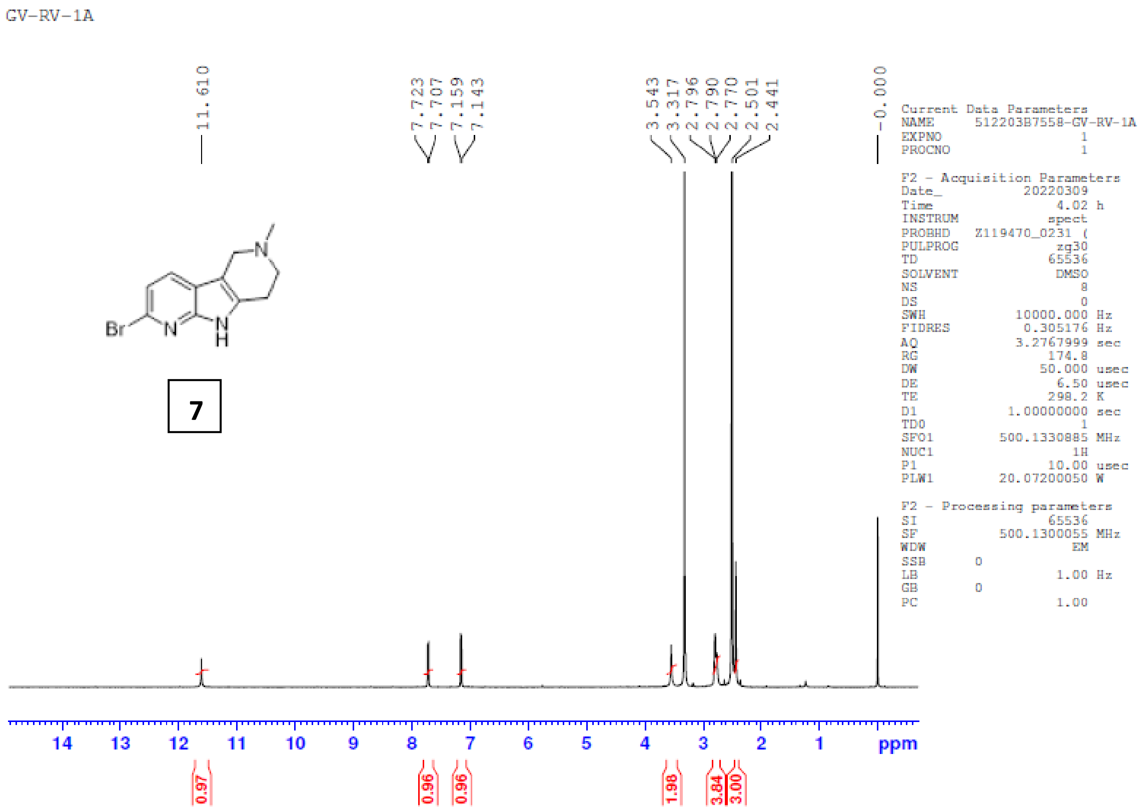

^{1}HNMR spectrum of 2-bromo-6-methyl-6,7,8,9-tetrahydro-5H-pyrrolo[2,3-b:4,5-c']dipyridine (7) in DMSO (500 MHz)

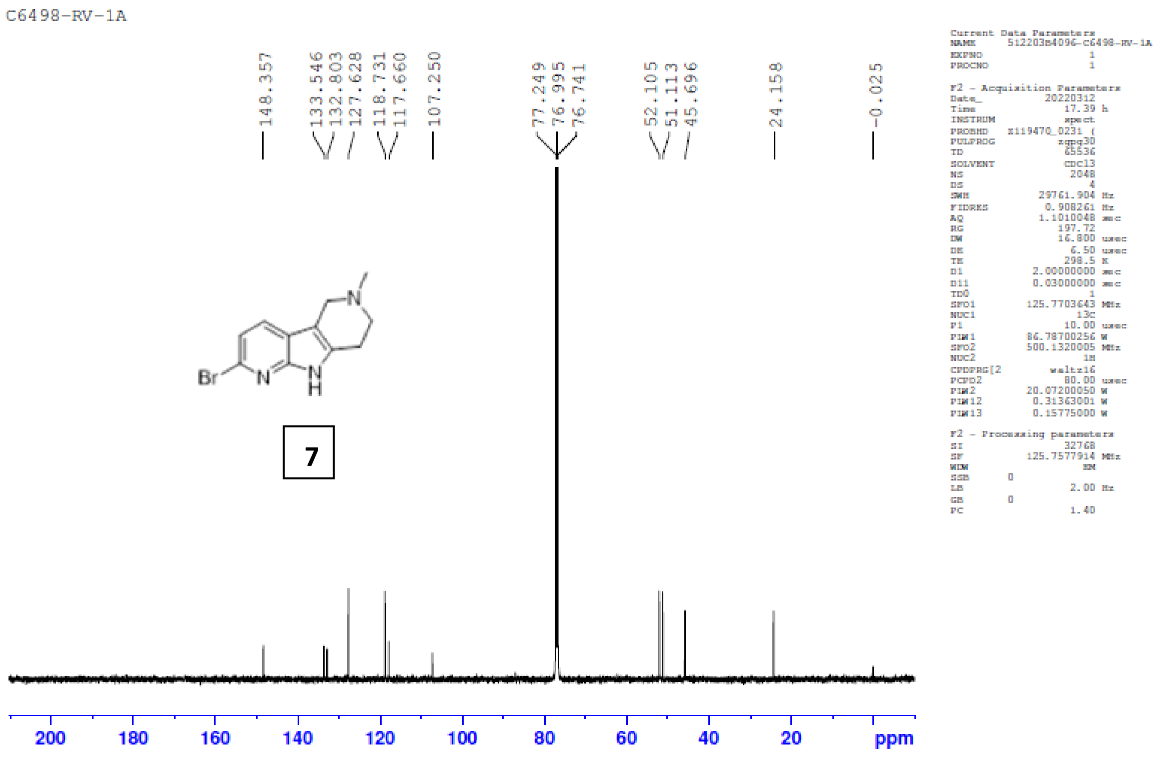

^{13}CNMR spectrum of 2-bromo-6-methyl-6,7,8,9-tetrahydro-5H-pyrrolo[2,3-b:4,5-c']dipyridine (7) in DMSO (125 MHz)

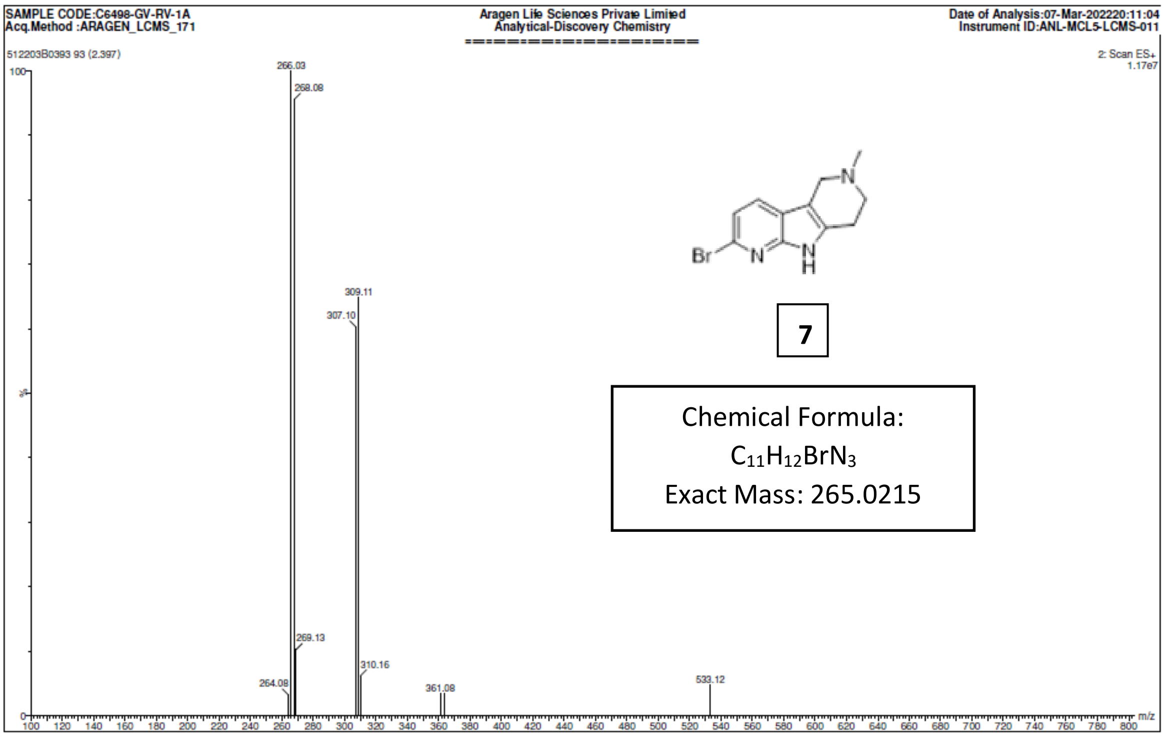

MASS spectrum of 2-bromo-6-methyl-6,7,8,9-tetrahydro-5H-pyrrolo[2,3-b:4,5-c']dipyridine(7)

Elemental Composition Report

Page 1

Single Mass Analysis

Tolerance = 820.0 PPM / DBE: min = -1.5, max = 50.0
Element prediction: Off
Number of isotope peaks used for i-FIT = 3

Monoisotopic Mass, Even Electron Ions
7 formula(e) evaluated with 1 results within limits (up to 50 best isotopic matches for each mass)
Elements Used:
C: 0-11 H: 0-13 N: 0-3 Br: 0-1

C6498-1A
Acq.method formic acid_4min
512209B4292 42 (1.069)

Aragene Life sciences Private Limited
Analytical-Discovery Chemistry

06-Sep-2022
21:59:51
INSTRUMENT-ID;ANL-MCL5-LCMS-013
1: Scan ES+
1.62e+007

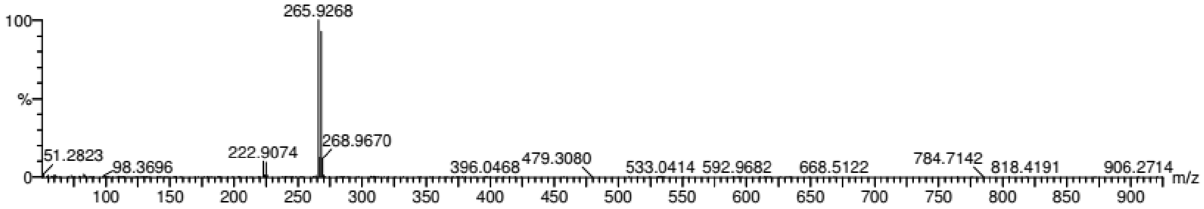

Minimum:				-1.5				
Maximum:		5.0	820.0	50.0				

Mass	Calc. Mass	mDa	PPM	DBE	i-FIT	Norm	Conf(%)	Formula
266.0285	266.0293	-0.8	-3.0	6.5	45.4	n/a	n/a	C11 H13 N3 Br

Elemental composition report of 2-bromo-6-methyl-6,7,8,9-tetrahydro-5H-pyrrolo[2,3-b:4,5-c']dipyridine(7)

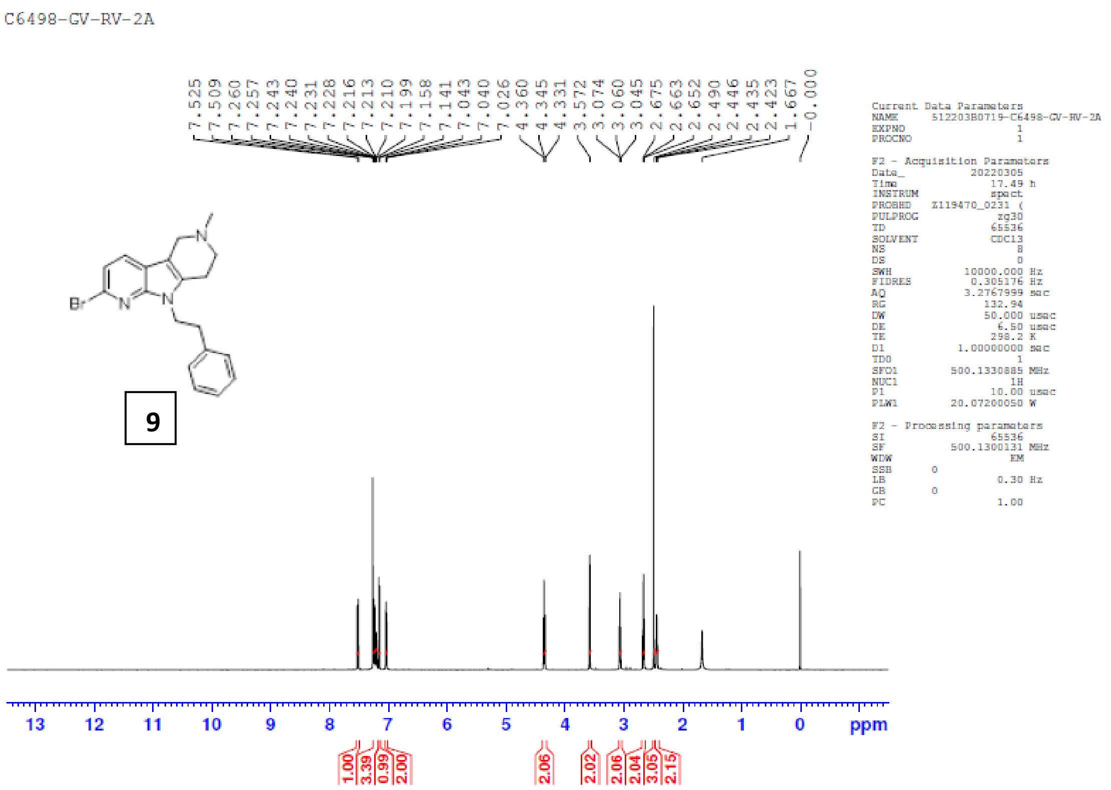

^{1}HNMR spectrum of 2-bromo-6-methyl-9-phenethyl-6,7,8,9-tetrahydro-5H-pyrrolo[2,3-b:4,5-c']dipyridine (9) in $CDCl_3$ (500 MHz)

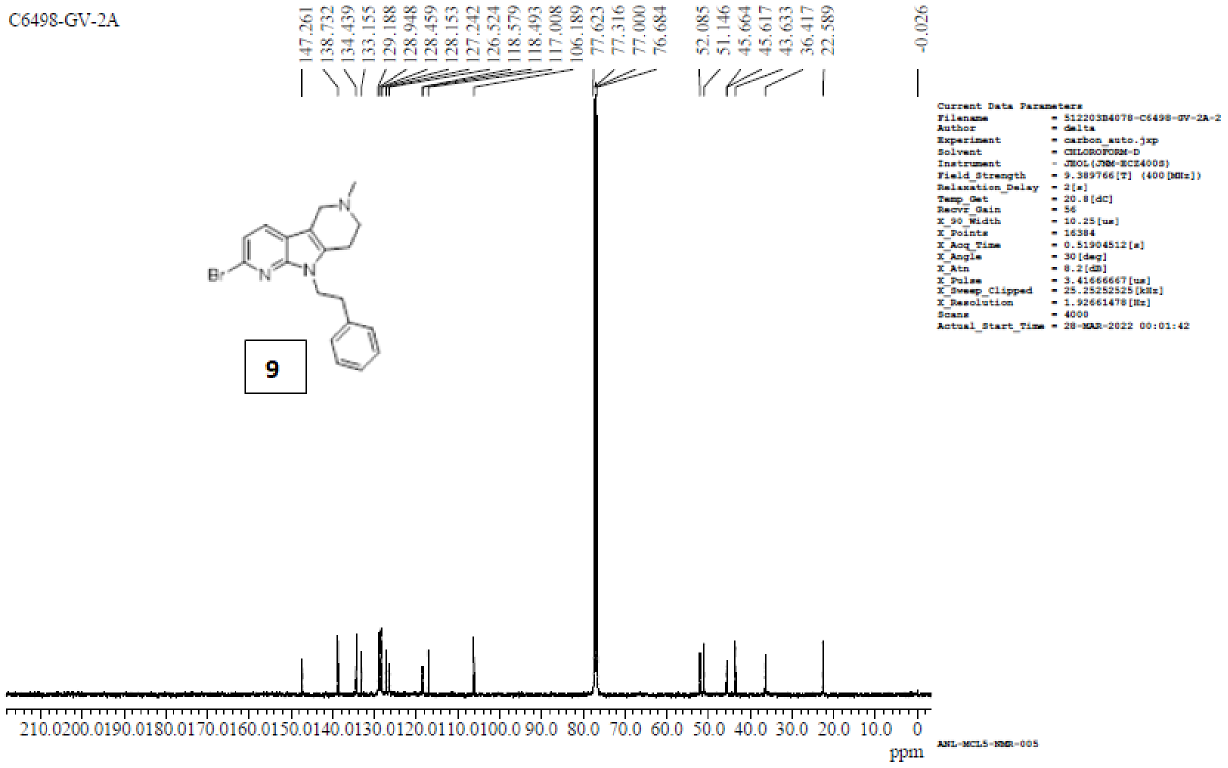

^{13}CNMR spectrum of 2-bromo-6-methyl-9-phenethyl-6,7,8,9-tetrahydro-5H-pyrrolo[2,3-b:4,5-c']dipyridine (9) in $CDCl_3$ (100 MHz)

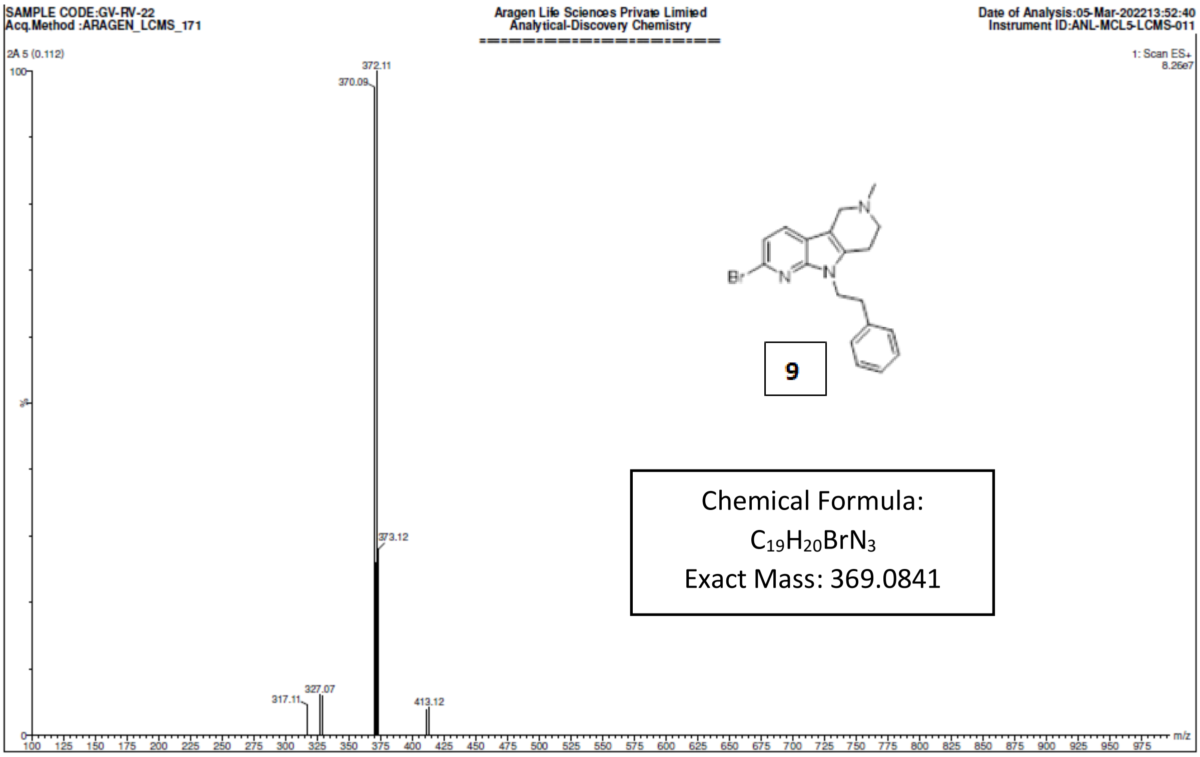

MASS spectrum of 2-bromo-6-methyl-9-phenethyl-6,7,8,9-tetrahydro-5H-pyrrolo[2,3-b:4,5-c']dipyridine (9)

Elemental Composition Report

Page 1

Single Mass Analysis

Tolerance = 820.0 PPM / DBE: min = -1.5, max = 50.0
Element prediction: Off
Number of isotope peaks used for i-FIT = 3

Monoisotopic Mass, Even Electron Ions
6 formula(e) evaluated with 1 results within limits (up to 50 best isotopic matches for each mass)
Elements Used:
C: 0-19 H: 0-21 N: 0-3 Br: 0-1

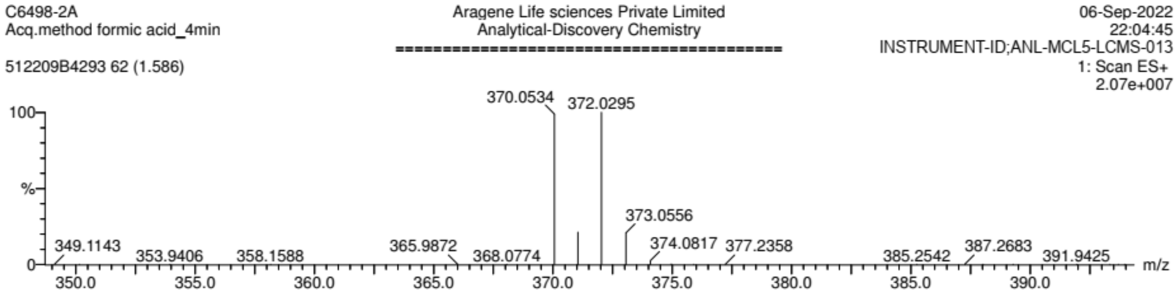

Minimum:				-1.5				
Maximum:		5.0	820.0	50.0				
Mass	Calc. Mass	mDa	PPM	DBE	i-FIT	Norm	Conf(%)	Formula
370.0901	370.0919	-1.8	-4.9	10.5	42.8	n/a	n/a	C19 H21 N3 Br

Elemental composition report of 2-bromo-6-methyl-9-phenethyl-6,7,8,9-tetrahydro-5H-pyrrolo[2,3-b:4,5-c']dipyridine (9)

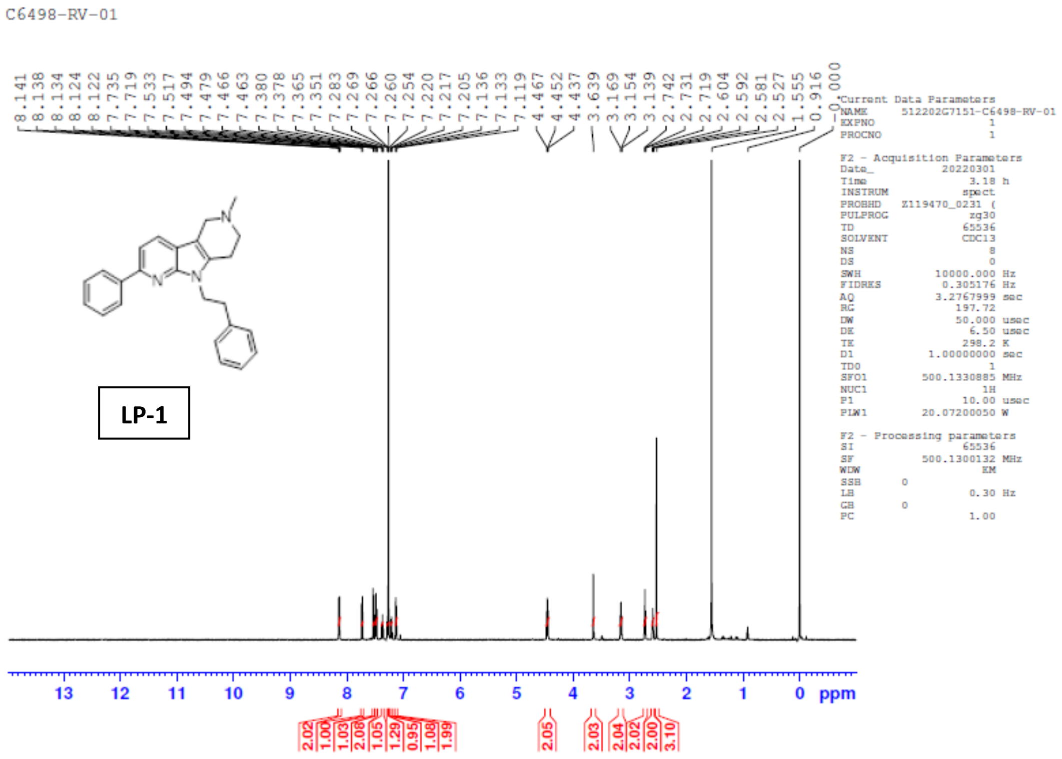

^{1}HNMR spectrum of 6-Methyl-9-phenethyl-2-phenyl-6,7,8,9-tetrahydro-5H-pyrrolo[2,3-b:4,5-c']dipyridine (LP-1) in $CDCl_3$ (500 MHz)

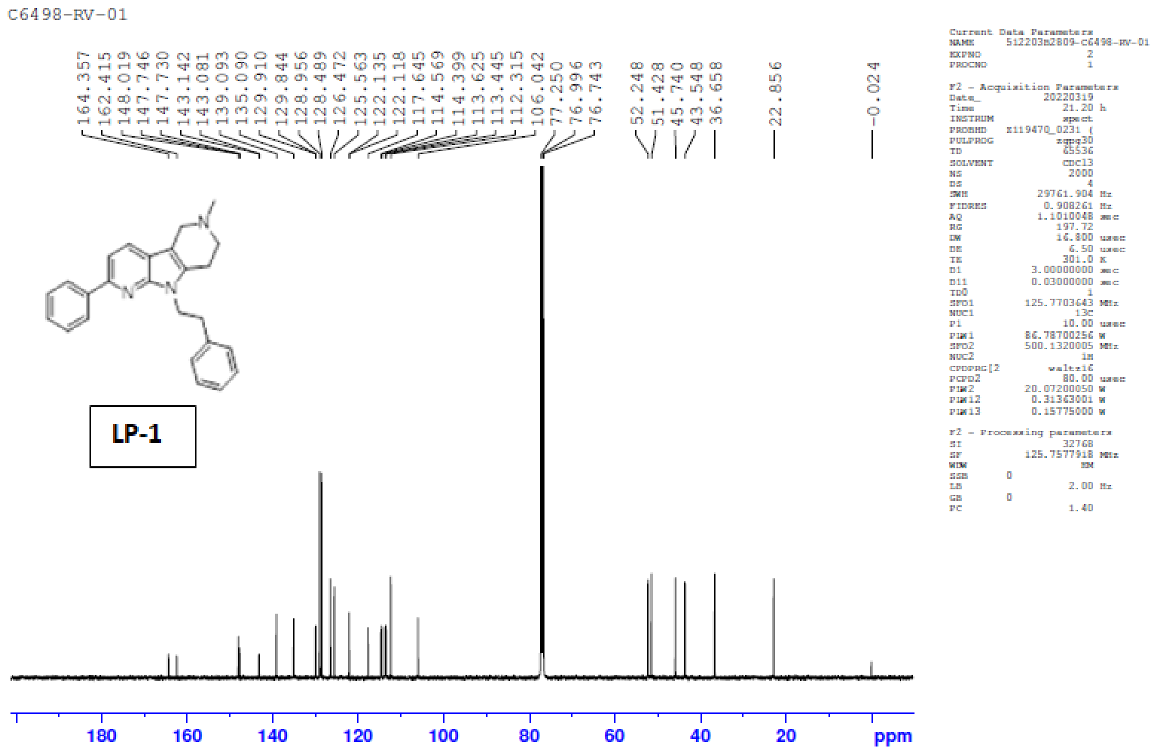

^{13}CNMR spectrum of 6-Methyl-9-phenethyl-2-phenyl-6,7,8,9-tetrahydro-5H-pyrrolo[2,3-b:4,5-c']dipyridine (LP-1) in $CDCl_3$ (500 MHz)

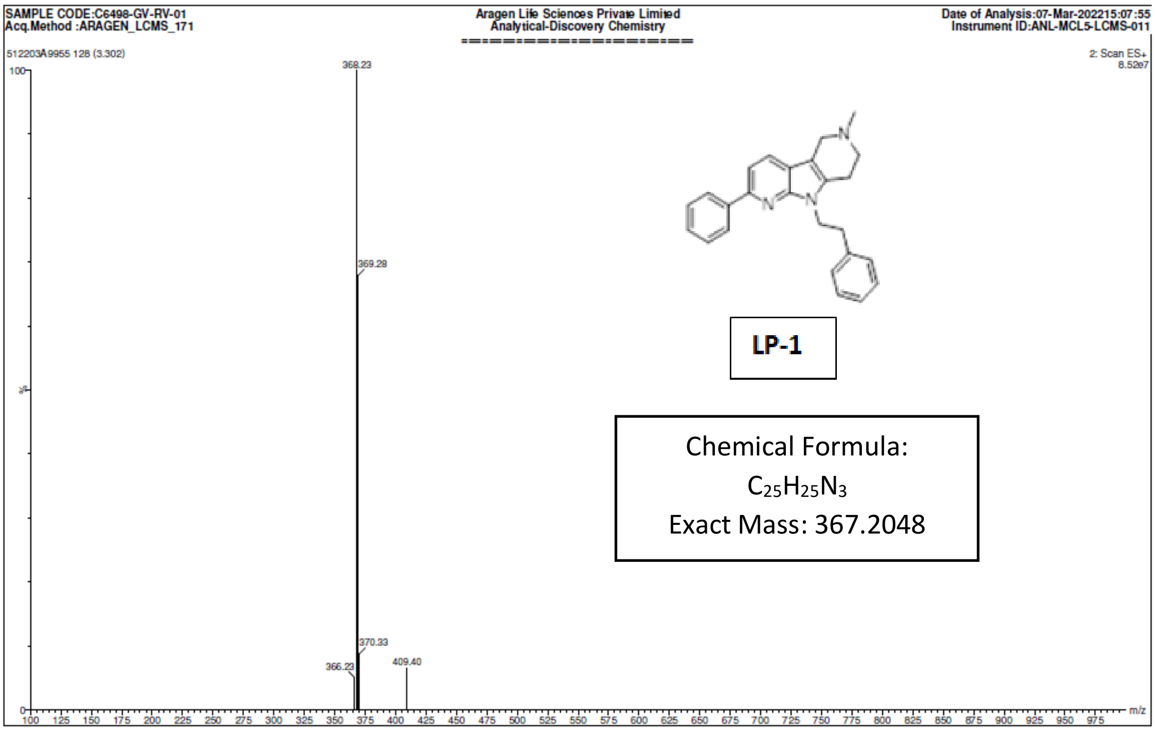

MASS spectrum of 6-Methyl-9-phenethyl-2-phenyl-6,7,8,9-tetrahydro-5H-pyrrolo[2,3-b:4,5-c']dipyridine (LP-1)

Elemental Composition Report **Page 1**

Single Mass Analysis

Tolerance = 820.0 PPM / DBE: min = -1.5, max = 50.0
Element prediction: Off
Number of isotope peaks used for i-FIT = 3

Monoisotopic Mass, Even Electron Ions
2 formula(e) evaluated with 1 results within limits (up to 50 best isotopic matches for each mass)
Elements Used:
C: 0-25 H: 0-26 N: 0-3

C6498-1 Aragene Life sciences Private Limited 06-Sep-2022
Acq.method formic acid_4min Analytical-Discovery Chemistry 21:45:12
INSTRUMENT-ID;ANL-MCL5-LCMS-013
512209B4295 70 (1.793) 1: Scan ES+
2.59e+007

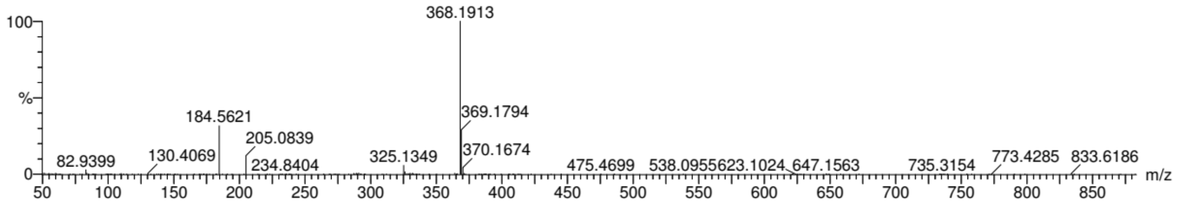

Minimum:				-1.5				
Maximum:		5.0	820.0	50.0				
Mass	Calc. Mass	mDa	PPM	DBE	i-FIT	Norm	Conf(%)	Formula
368.2120	368.2127	-0.7	-1.9	14.5	37.9	n/a	n/a	C25 H26 N3

Elemental composition report of 6-Methyl-9-phenethyl-2-phenyl-6,7,8,9-tetrahydro-5H-pyrrolo[2,3-b:4,5-c']dipyridine (LP-1)

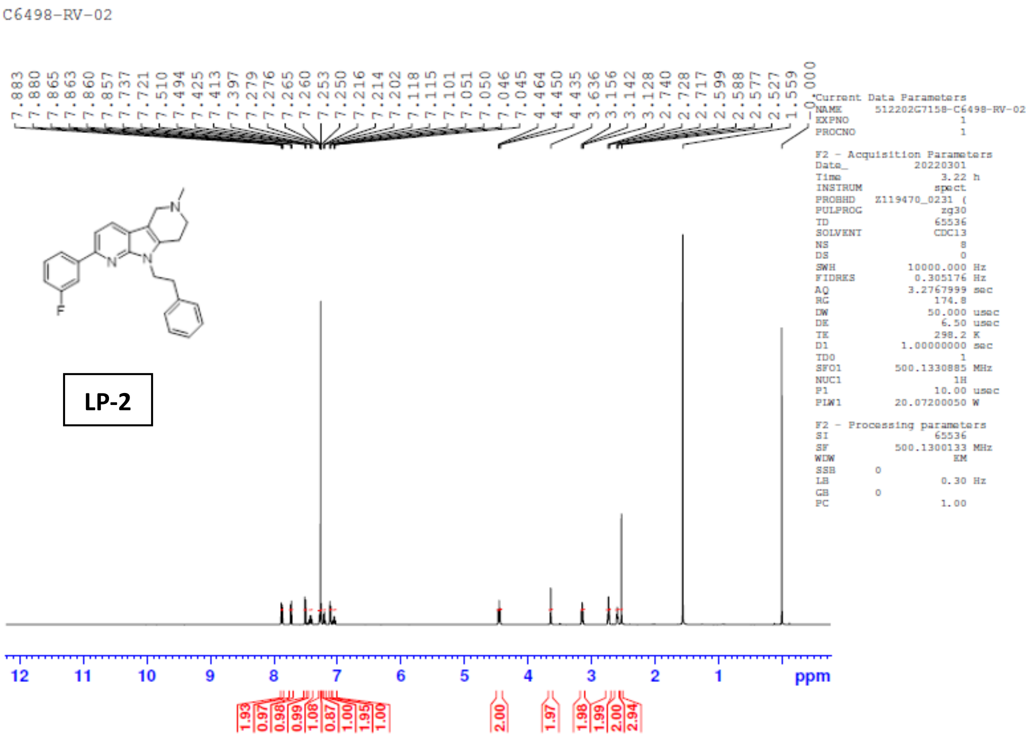

^{1}HNMR spectrum of 2-(3-Fluorophenyl)-6-methyl-9-phenethyl-6,7,8,9-tetrahydro-5H-pyrrolo[2,3-b:4,5-c']dipyridine (LP-2) in $CDCl_3$ (500 MHz)

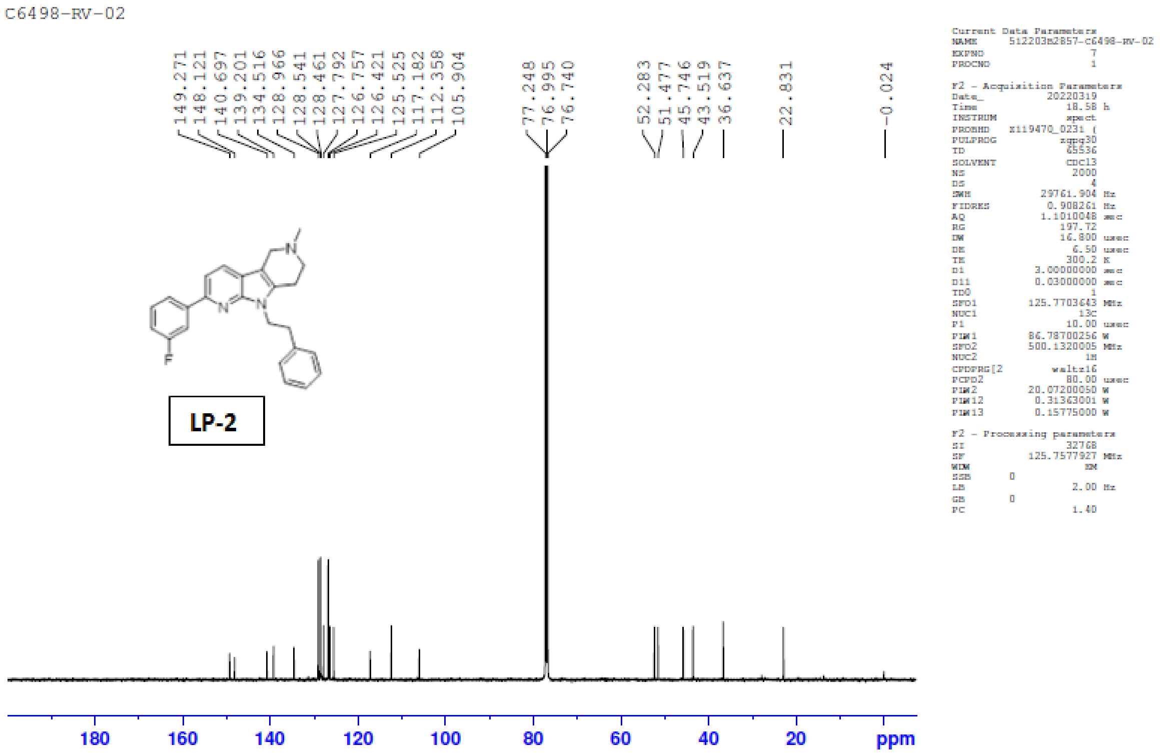

^{13}CNMR spectrum of 2-(3-Fluorophenyl)-6-methyl-9-phenethyl-6,7,8,9-tetrahydro-5H-pyrrolo[2,3-b:4,5-c']dipyridine (LP-2) in $CDCl_3$ (125 MHz)

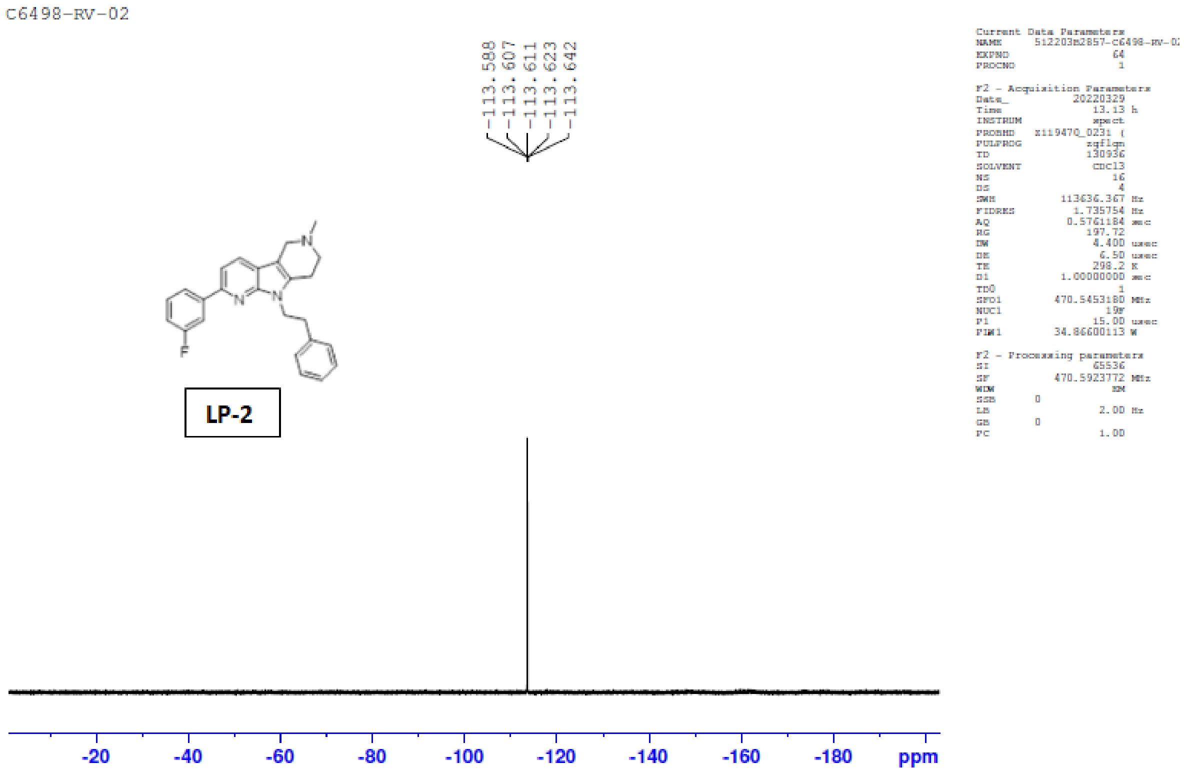

^{19}FNMR spectrum of 2-(3-Fluorophenyl)-6-methyl-9-phenethyl-6,7,8,9-tetrahydro-5H-pyrrolo[2,3-b:4,5-c']dipyridine (LP-2) in $CDCl_3$ (470 MHz)

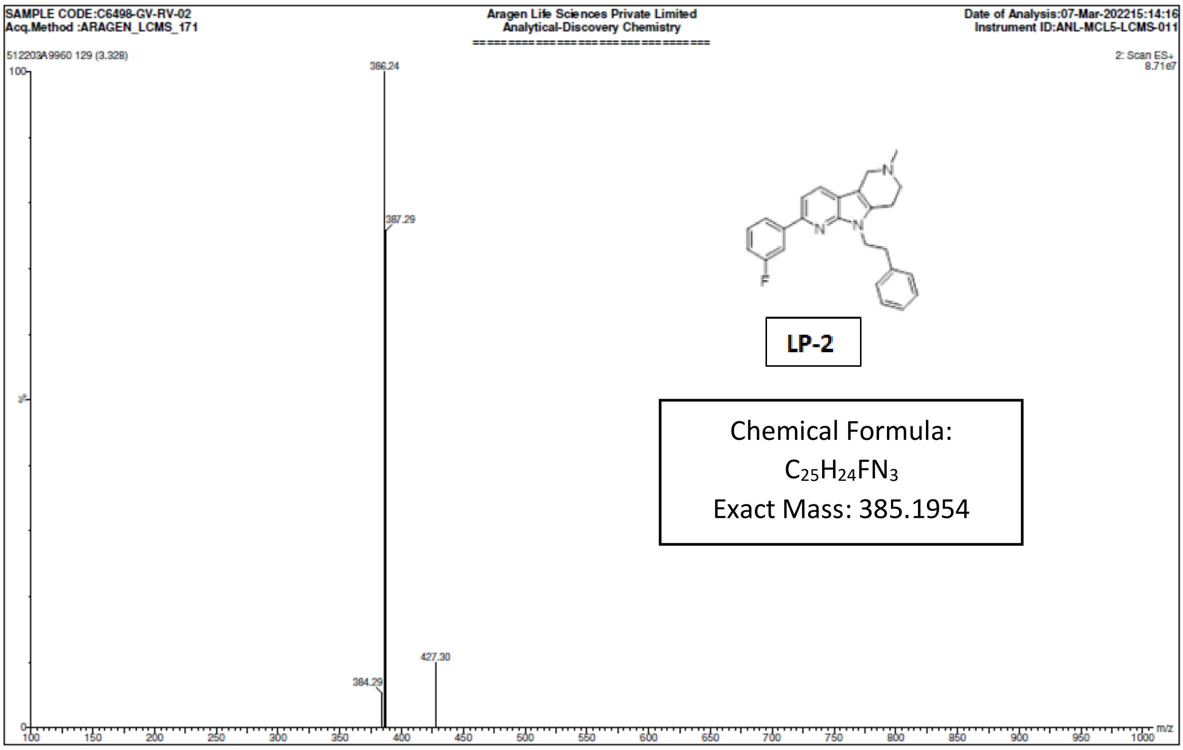

MASS spectrum of 2-(3-Fluorophenyl)-6-methyl-9-phenethyl-6,7,8,9-tetrahydro-5H-pyrrolo[2,3-b:4,5-c']dipyridine (LP-2)

Elemental Composition Report Page 1

Single Mass Analysis
Tolerance = 820.0 PPM / DBE: min = -1.5, max = 50.0
Element prediction: Off
Number of isotope peaks used for i-FIT = 3

Monoisotopic Mass, Even Electron Ions
4 formula(e) evaluated with 1 results within limits (up to 50 best isotopic matches for each mass)
Elements Used:
C: 0-25 H: 0-25 N: 0-3 F: 0-1

C6498-2
Acq.method formic acid_4min
Aragene Life sciences Private Limited
Analytical-Discovery Chemistry
06-Sep-2022
21:50:04
INSTRUMENT-ID;ANL-MCL5-LCMS-013
512209B4296 71 (1.819)
1: Scan ES+
4.05e+007

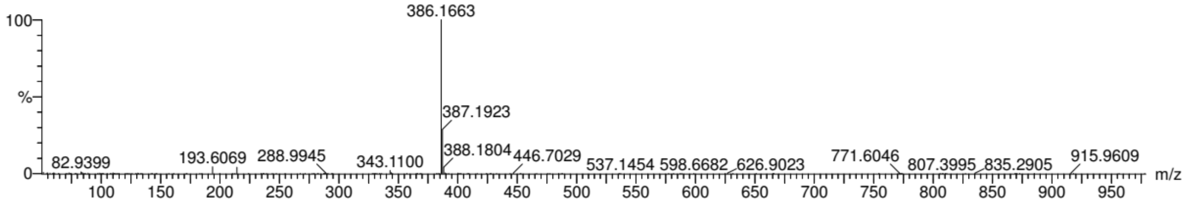

Minimum:				-1.5				
Maximum:		5.0	820.0	50.0				
Mass	Calc. Mass	mDa	PPM	DBE	i-FIT	Norm	Conf(%)	Formula
386.2001	386.2033	-3.2	-8.3	14.5	38.7	n/a	n/a	C25 H25 N3 F

Elemental composition report of 2-(3-Fluorophenyl)-6-methyl-9-phenethyl-6,7,8,9-tetrahydro-5H-pyrrolo[2,3-b:4,5-c']dipyridine (LP-2)

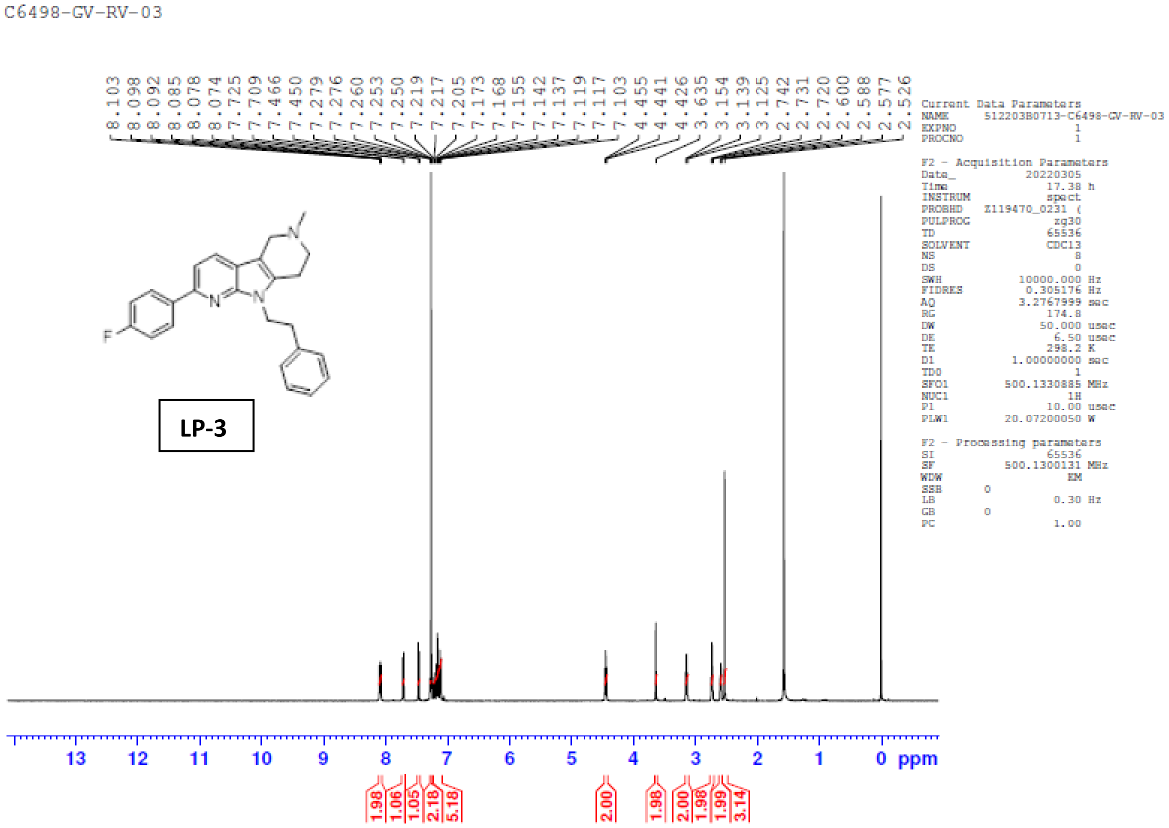

^{1}HNMR spectrum of 2-(4-Fluorophenyl)-6-methyl-9-phenethyl-6,7,8,9-tetrahydro-5H-pyrrolo[2,3-b:4,5-c']dipyridine (LP-3) in $CDCl_3$ (500 MHz)

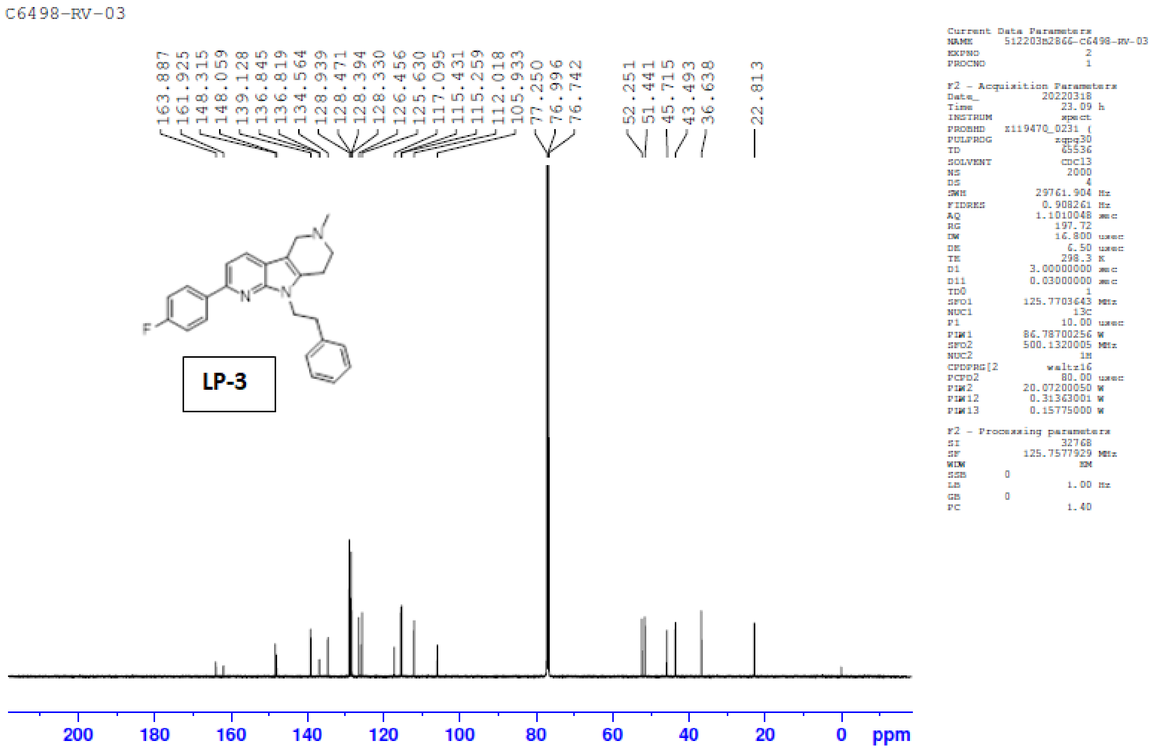

^{13}CNMR spectrum of 2-(4-Fluorophenyl)-6-methyl-9-phenethyl-6,7,8,9-tetrahydro-5H-pyrrolo[2,3-b:4,5-c']dipyridine (LP-3) in $CDCl_3$ (125 MHz)

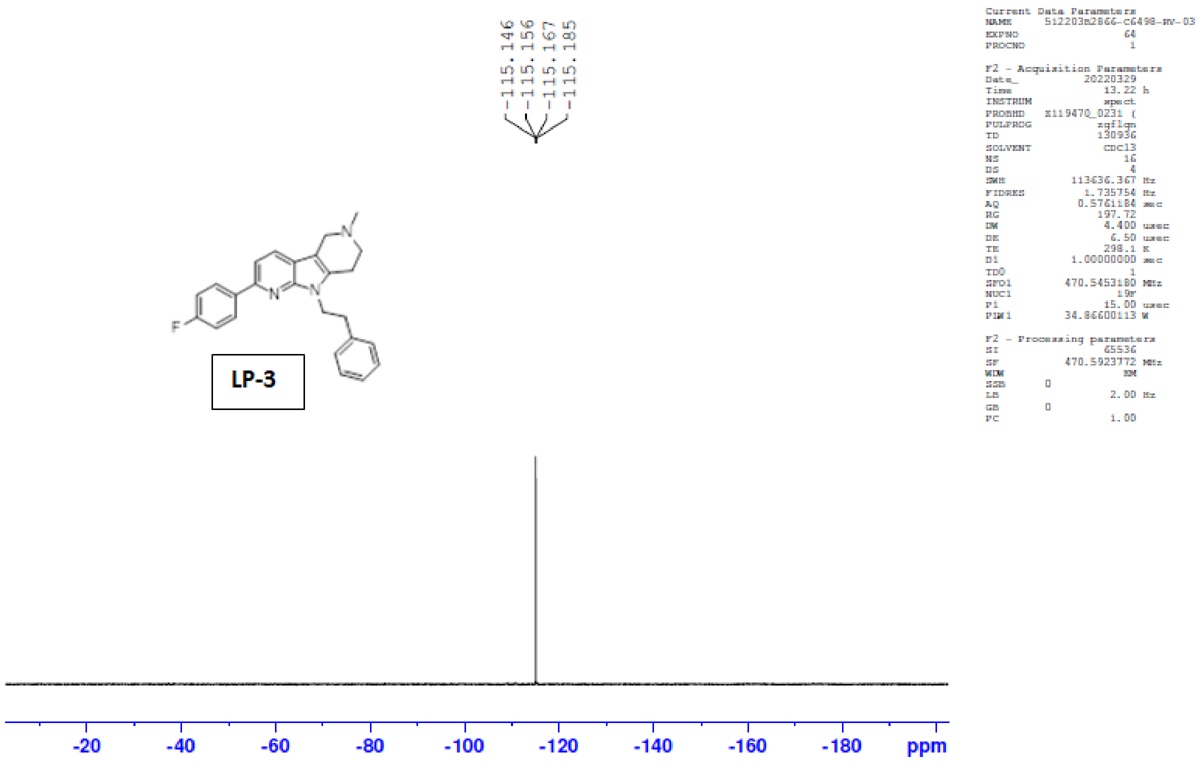

^{19}FNMR spectrum of 2-(4-Fluorophenyl)-6-methyl-9-phenethyl-6,7,8,9-tetrahydro-5H-pyrrolo[2,3-b:4,5-c']dipyridine (LP-3) in $CDCl_3$ (470 MHz)

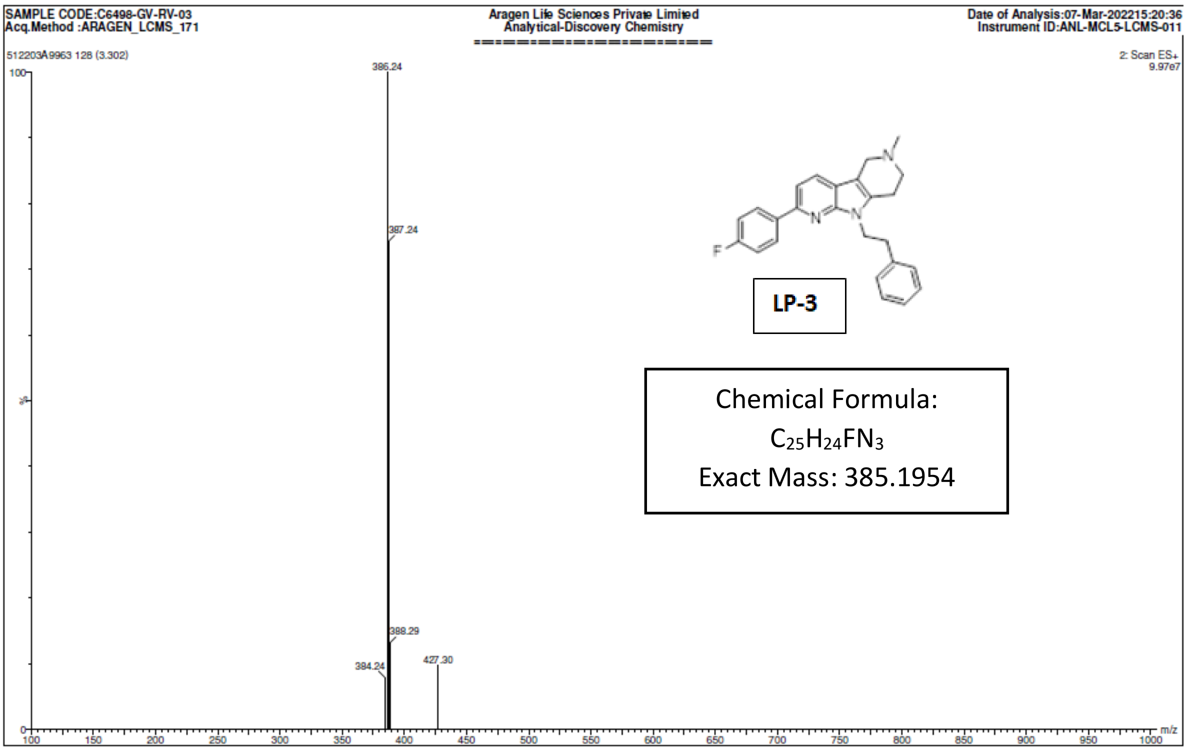

MASS spectrum of 2-(4-Fluorophenyl)-6-methyl-9-phenethyl-6,7,8,9-tetrahydro-5H-pyrrolo[2,3-b:4,5-c']dipyridine (LP-3)

Elemental Composition Report

Page 1

Single Mass Analysis

Tolerance = 500.0 PPM / DBE: min = -1.5, max = 50.0
Element prediction: Off
Number of isotope peaks used for i-FIT = 3

Monoisotopic Mass, Even Electron Ions
4 formula(e) evaluated with 1 results within limits (up to 50 best isotopic matches for each mass)
Elements Used:
C: 0-25 H: 0-25 N: 0-3 F: 0-1

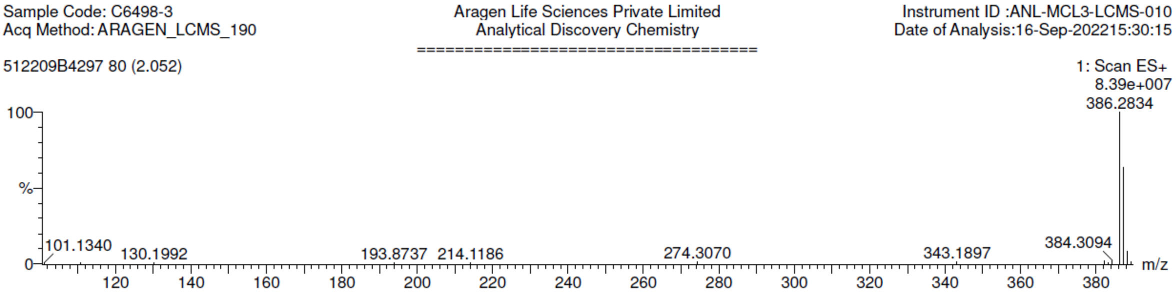

Minimum:				-1.5				
Maximum:		5.0	500.0	50.0				
Mass	Calc. Mass	mDa	PPM	DBE	i-FIT	Norm	Conf(%)	Formula
386.2060	386.2033	2.7	7.0	14.5	61.0	n/a	n/a	C25 H25 N3 F

Elemental analysis report of 2-(4-Fluorophenyl)-6-methyl-9-phenethyl-6,7,8,9-tetrahydro-5H-pyrrolo[2,3-b:4,5-c']dipyridine (LP-3)

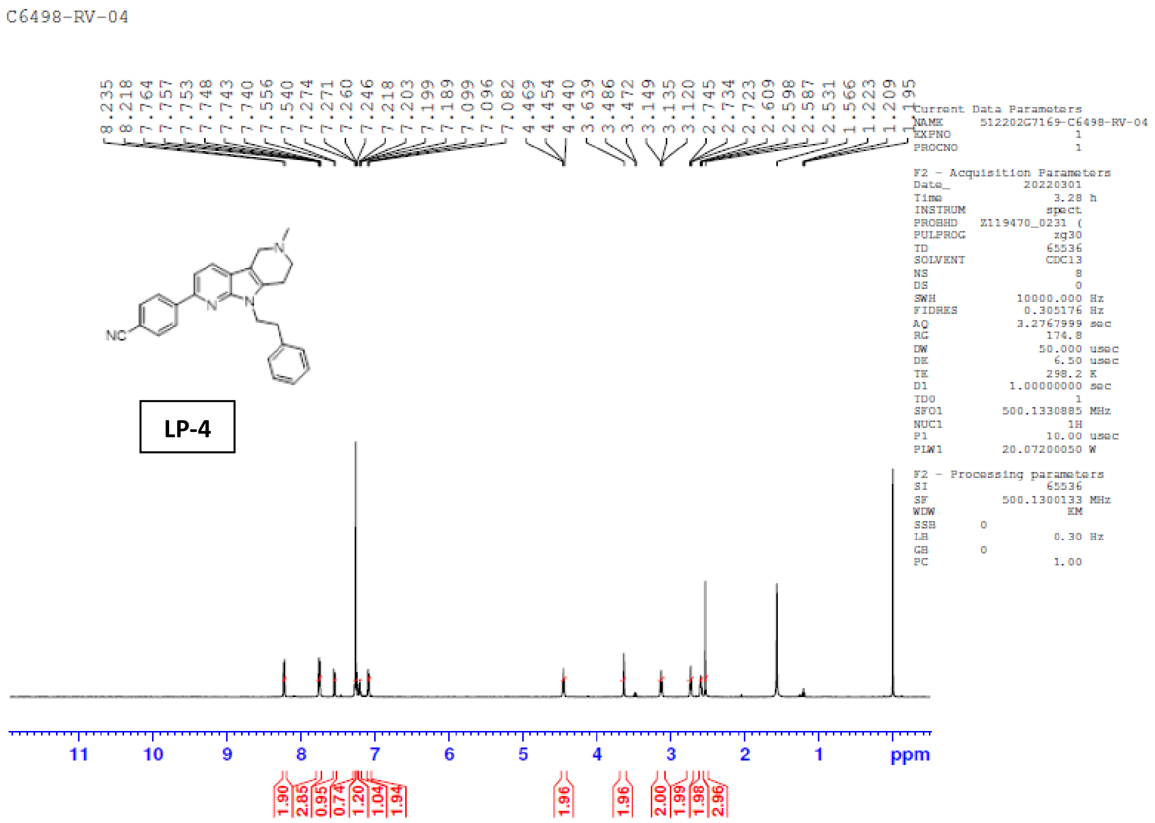

^{1}HNMR spectrum of 4-(6-Methyl-9-phenethyl-6,7,8,9-tetrahydro-5H-pyrrolo[2,3-b:4,5-c']dipyridin-2-yl)benzonitrile (LP-4) in $CDCl_3$ (500 MHz)

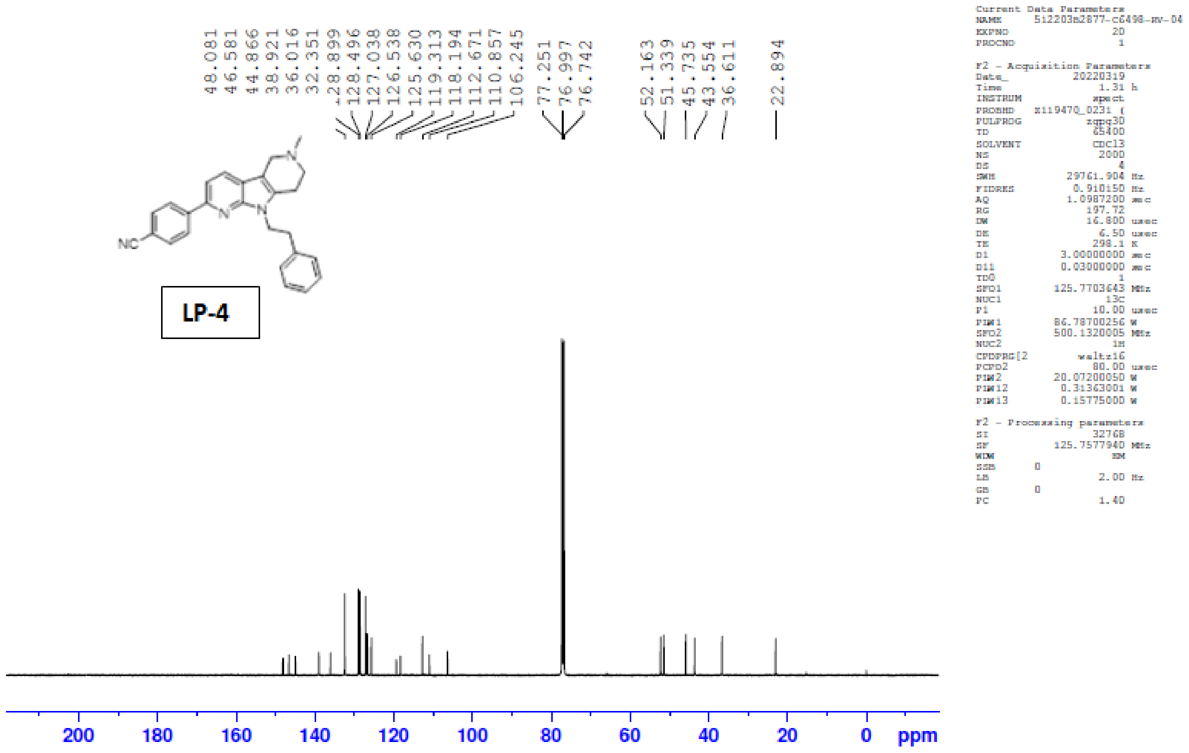

^{13}CNMR spectrum of 4-(6-Methyl-9-phenethyl-6,7,8,9-tetrahydro-5H-pyrrolo[2,3-b:4,5-c']dipyridin-2-yl)benzonitrile (LP-4) in $CDCl_3$ (125 MHz)

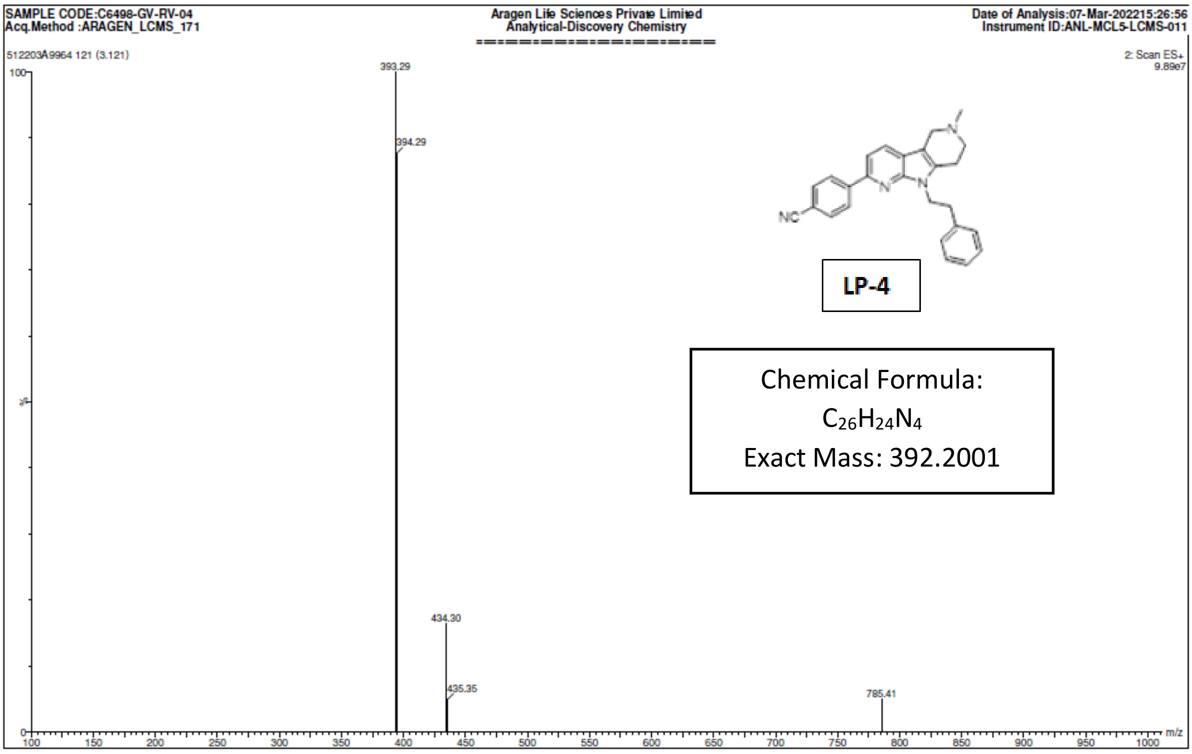

MASS spectrum of 4-(6-Methyl-9-phenethyl-6,7,8,9-tetrahydro-5H-pyrrolo[2,3-b:4,5-c']dipyridin-2-yl)benzonitrile (LP-4)

Elemental Composition Report **Page 1**

Single Mass Analysis
Tolerance = 500.0 PPM / DBE: min = -1.5, max = 50.0
Element prediction: Off
Number of isotope peaks used for i-FIT = 3

Monoisotopic Mass, Even Electron Ions
3 formula(e) evaluated with 1 results within limits (up to 50 best isotopic matches for each mass)
Elements Used:
C: 0-26 H: 0-25 N: 0-4

Sample Code: C6498-4
Acq Method: ARAGEN_LCMS_190

Aragen Life Sciences Private Limited
Analytical Discovery Chemistry

Instrument ID :ANL-MCL3-LCMS-010
Date of Analysis:16-Sep-202215:36:20

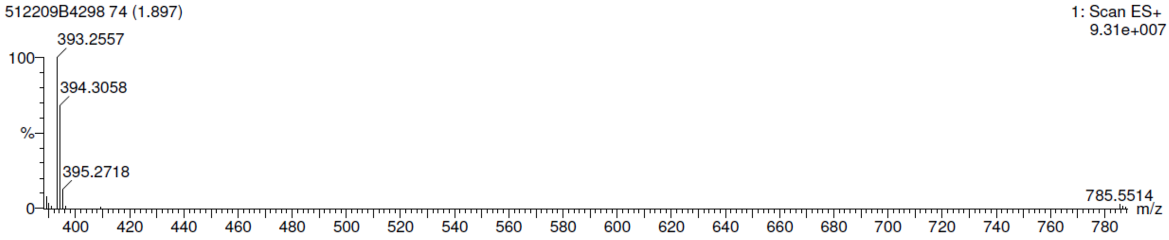

Minimum:				-1.5				
Maximum:		5.0	500.0	50.0				
Mass	Calc. Mass	mDa	PPM	DBE	i-FIT	Norm	Conf(%)	Formula
393.2050	393.2079	-2.9	-7.4	16.5	63.1	n/a	n/a	C26 H25 N4

Elemental analysis report of 4-(6-Methyl-9-phenethyl-6,7,8,9-tetrahydro-5H-pyrrolo[2,3-b:4,5-c']dipyridin-2-yl)benzonitrile (LP-4)

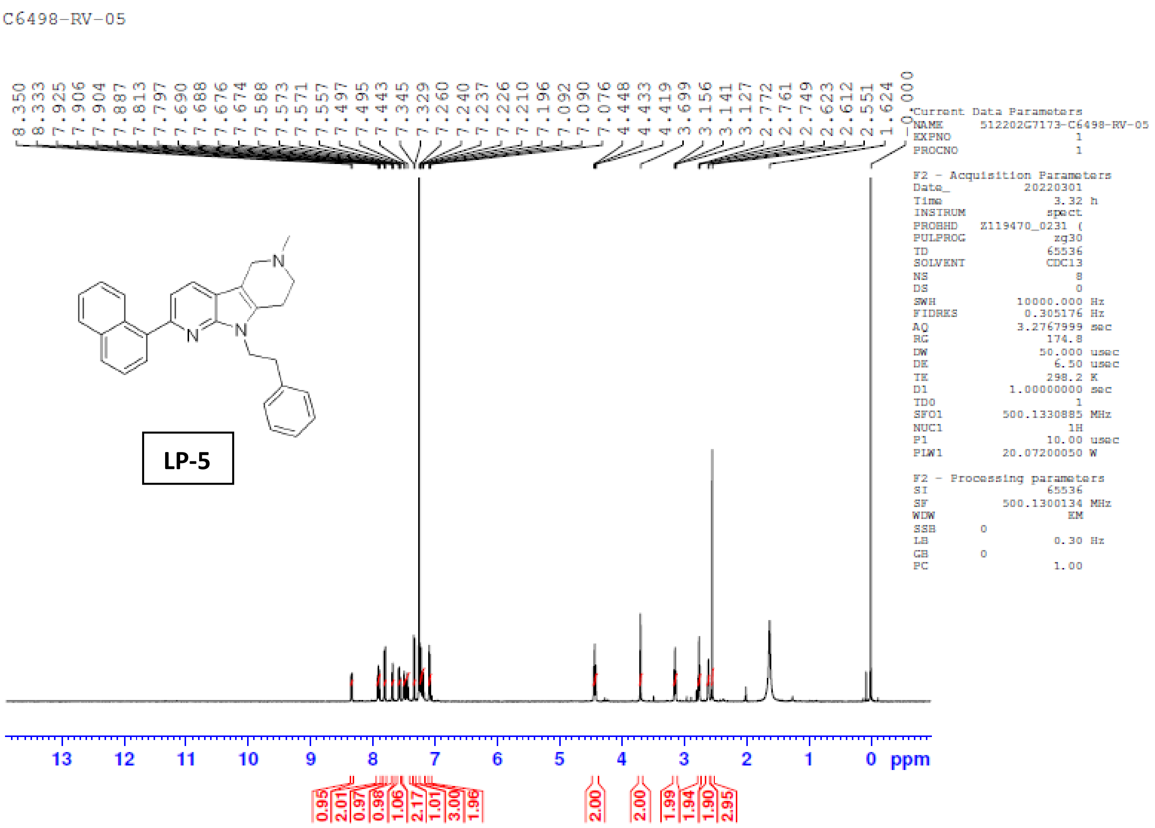

^{1}HNMR spectrum of 6-Methyl-2-(naphthalen-1-yl)-9-phenethyl-6,7,8,9-tetrahydro-5H-pyrrolo[2,3-b:4,5-c']dipyridine (LP-5) in $CDCl_3$ (500 MHz)

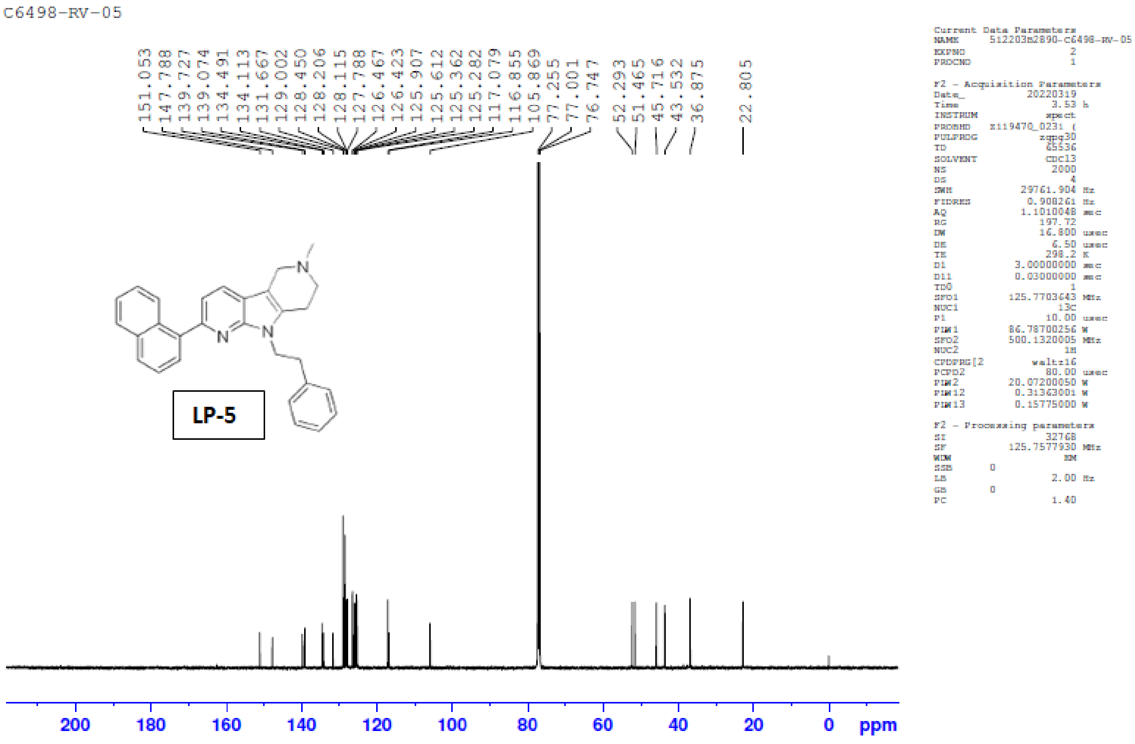

^{13}CNMR spectrum of 6-Methyl-2-(naphthalen-1-yl)-9-phenethyl-6,7,8,9-tetrahydro-5H-pyrrolo[2,3-b:4,5-c']dipyridine (LP-5) in $CDCl_3$ (125 MHz)

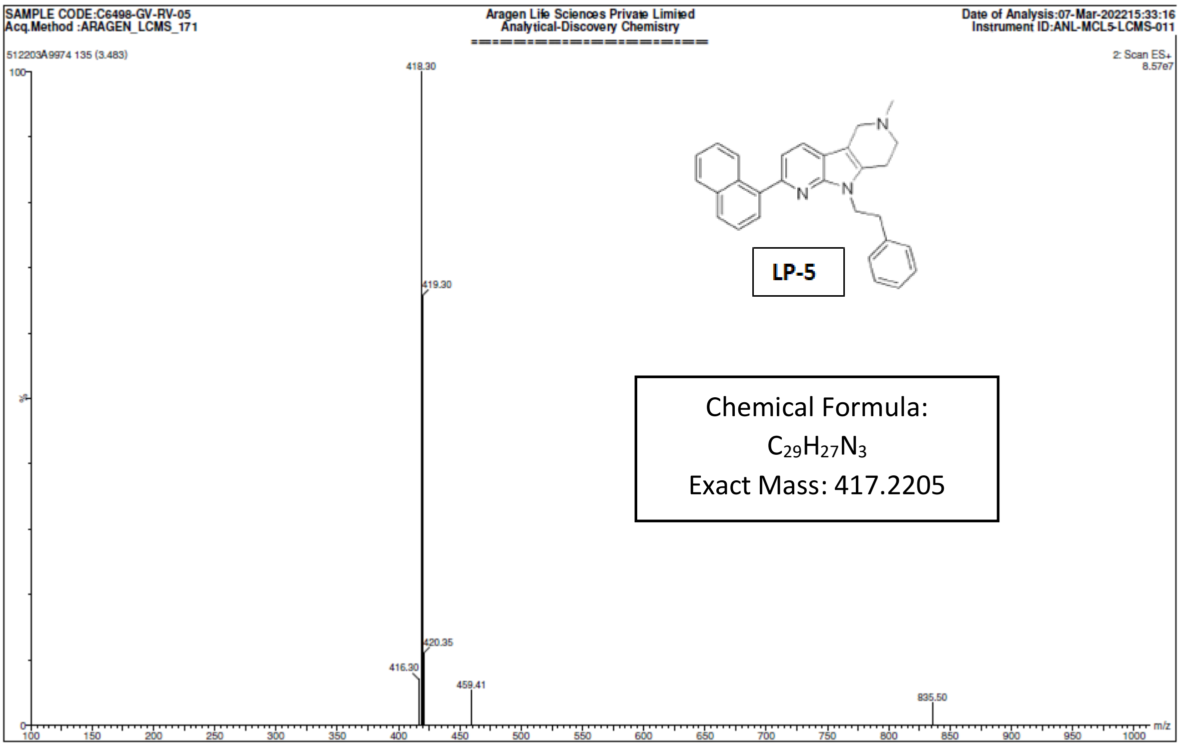

MASS spectrum of 6-Methyl-2-(naphthalen-1-yl)-9-phenethyl-6,7,8,9-tetrahydro-5H-pyrrolo[2,3-b:4,5-c']dipyridine (LP-5)

Elemental Composition Report

Page 1

Single Mass Analysis

Tolerance = 500.0 PPM / DBE: min = -1.5, max = 50.0
Element prediction: Off
Number of isotope peaks used for i-FIT = 3

Monoisotopic Mass, Even Electron Ions
1 formula(e) evaluated with 1 results within limits (up to 50 best isotopic matches for each mass)
Elements Used:
C: 0-29 H: 0-28 N: 0-3

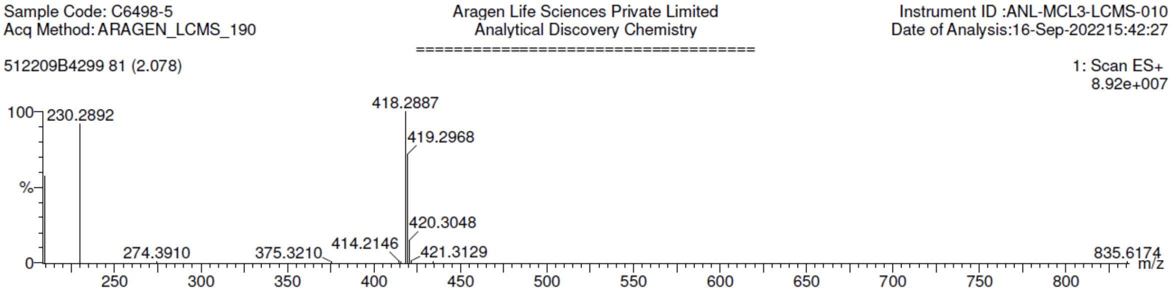

Minimum:				-1.5				
Maximum:		5.0	500.0	50.0				
Mass	Calc. Mass	mDa	PPM	DBE	i-FIT	Norm	Conf(%)	Formula
418.2243	418.2283	-4.0	-9.6	17.5	56.2	n/a	n/a	C29 H28 N3

Elemental composition report of 6-Methyl-2-(naphthalen-1-yl)-9-phenethyl-6,7,8,9-tetrahydro-5H-pyrrolo[2,3-b:4,5-c']dipyridine (LP-5)

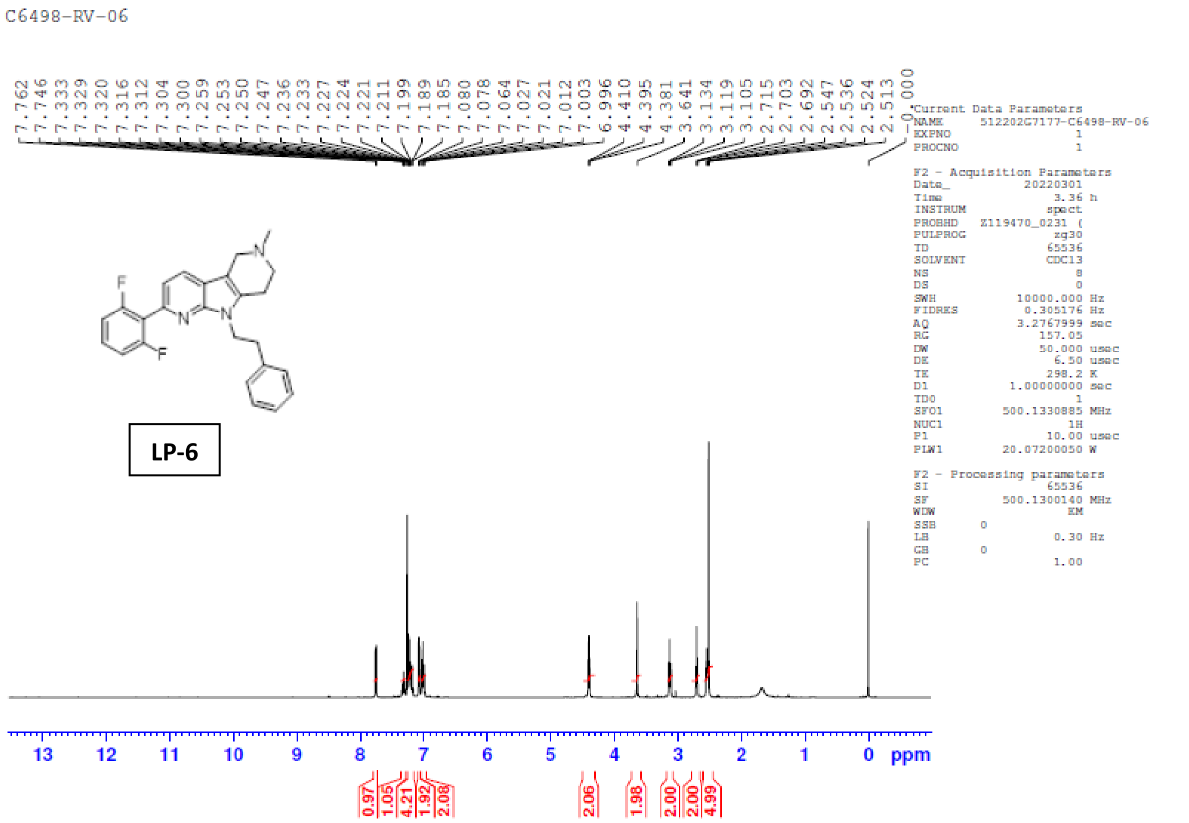

^{1}HNMR spectrum of 2-(2,6-Difluorophenyl)-6-methyl-9-phenethyl-6,7,8,9-tetrahydro-5H-pyrrolo[2,3-b:4,5-c']dipyridine (LP-6) in $CDCl_3$ (500 MHz)

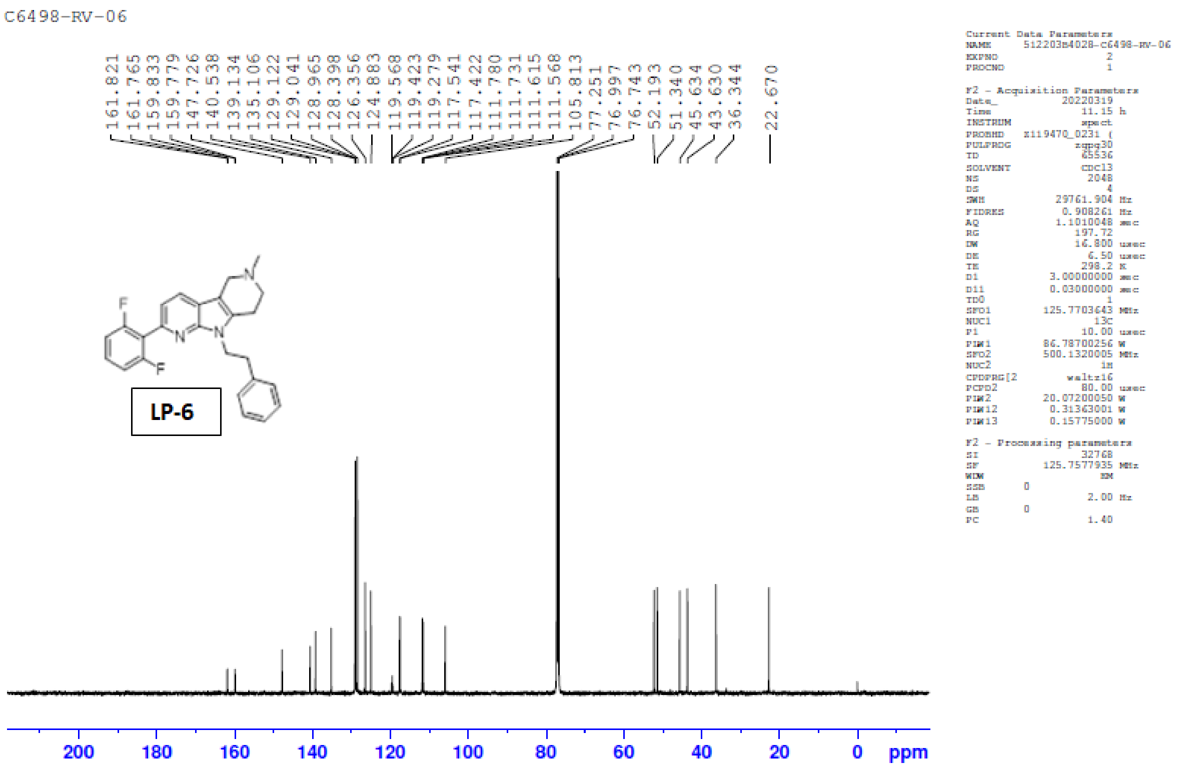

^{13}CNMR spectrum of 2-(2,6-Difluorophenyl)-6-methyl-9-phenethyl-6,7,8,9-tetrahydro-5H-pyrrolo[2,3-b:4,5-c']dipyridine (LP-6) in $CDCl_3$ (125 MHz)

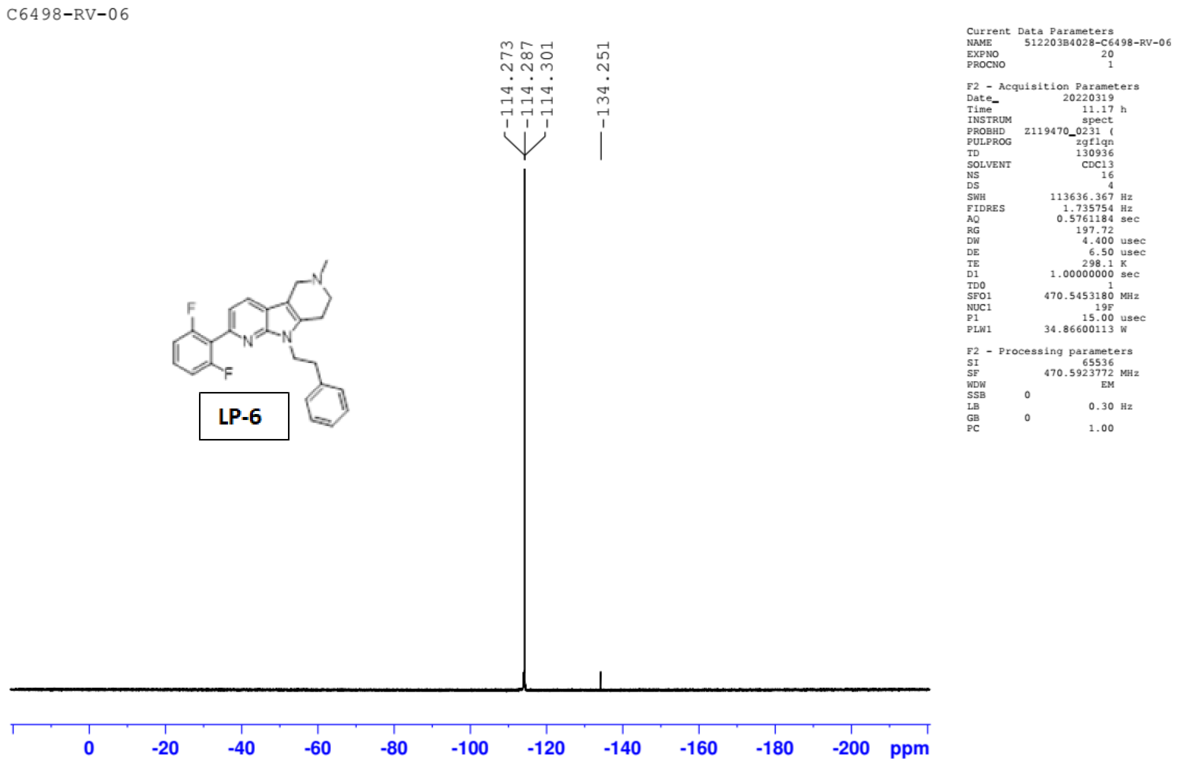

^{19}FNMR spectrum of 2-(2,6-Difluorophenyl)-6-methyl-9-phenethyl-6,7,8,9-tetrahydro-5H-pyrrolo[2,3-b:4,5-c']dipyridine (LP-6) in $CDCl_3$ (470 MHz)

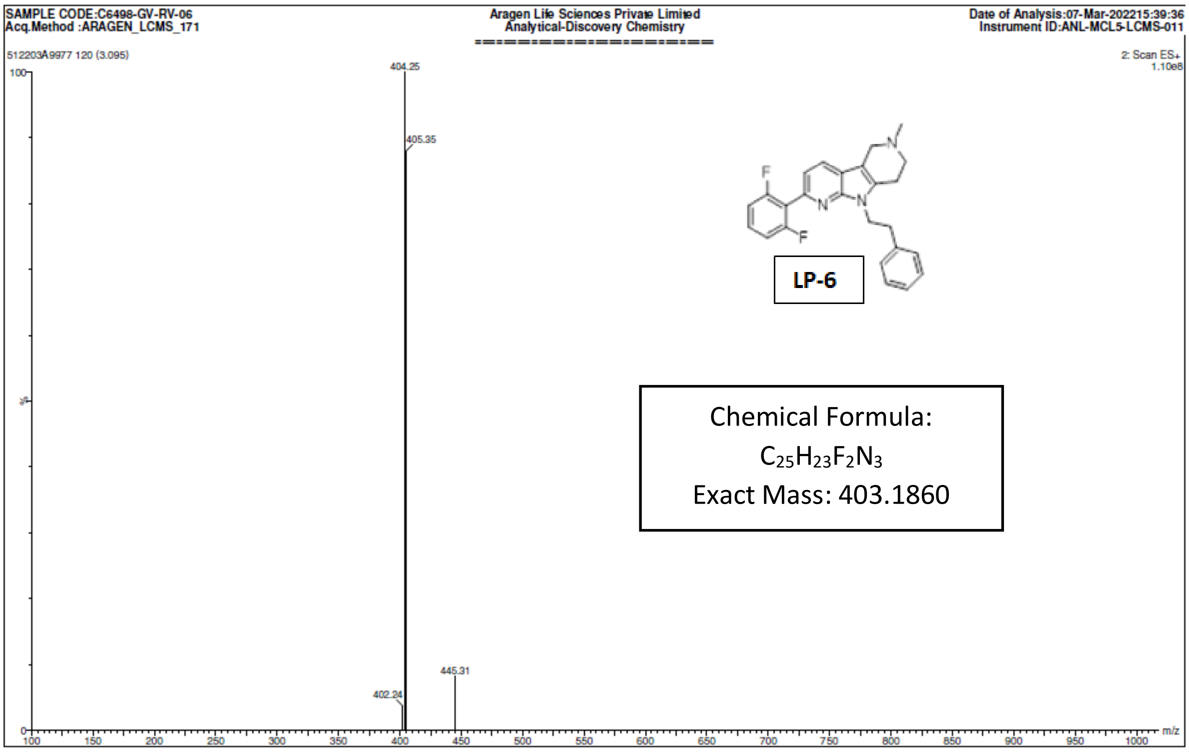

MASS spectrum of 2-(2,6-Difluorophenyl)-6-methyl-9-phenethyl-6,7,8,9-tetrahydro-5H-pyrrolo[2,3-b:4,5-c']dipyridine (LP-6)

Elemental Composition Report

Page 1

Single Mass Analysis
Tolerance = 500.0 PPM / DBE: min = -1.5, max = 50.0
Element prediction: Off
Number of isotope peaks used for i-FIT = 3

Monoisotopic Mass, Even Electron Ions
7 formula(e) evaluated with 1 results within limits (up to 50 best isotopic matches for each mass)
Elements Used:
C: 0-25 H: 0-24 N: 0-3 F: 0-2

Sample Code: C6498-6
Acq Method: ARAGEN_LCMS_190

Aragen Life Sciences Private Limited
Analytical Discovery Chemistry

Instrument ID :ANL-MCL3-LCMS-010
Date of Analysis:16-Sep-202215:48:25

512209B4300 76 (1.949)

1: Scan ES+
1.02e+008

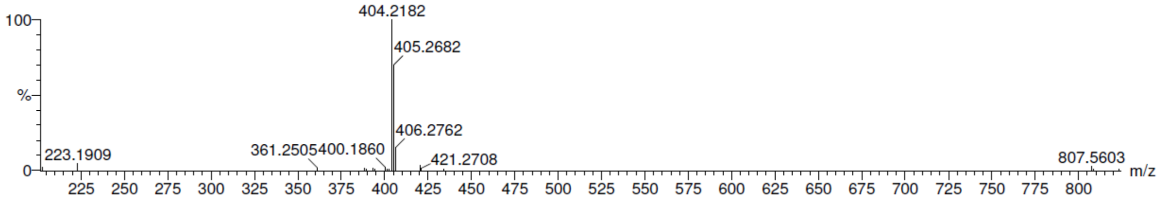

Minimum:				-1.5				
Maximum:		5.0	500.0	50.0				
Mass	Calc. Mass	mDa	PPM	DBE	i-FIT	Norm	Conf(%)	Formula
404.1958	404.1938	2.0	4.9	14.5	64.4	n/a	n/a	C25 H24 N3 F2

Elemental composition report of 2-(2,6-Difluorophenyl)-6-methyl-9-phenethyl-6,7,8,9-tetrahydro-5H-pyrrolo[2,3-b:4,5-c']dipyridine (LP-6)

C6498-RV-07

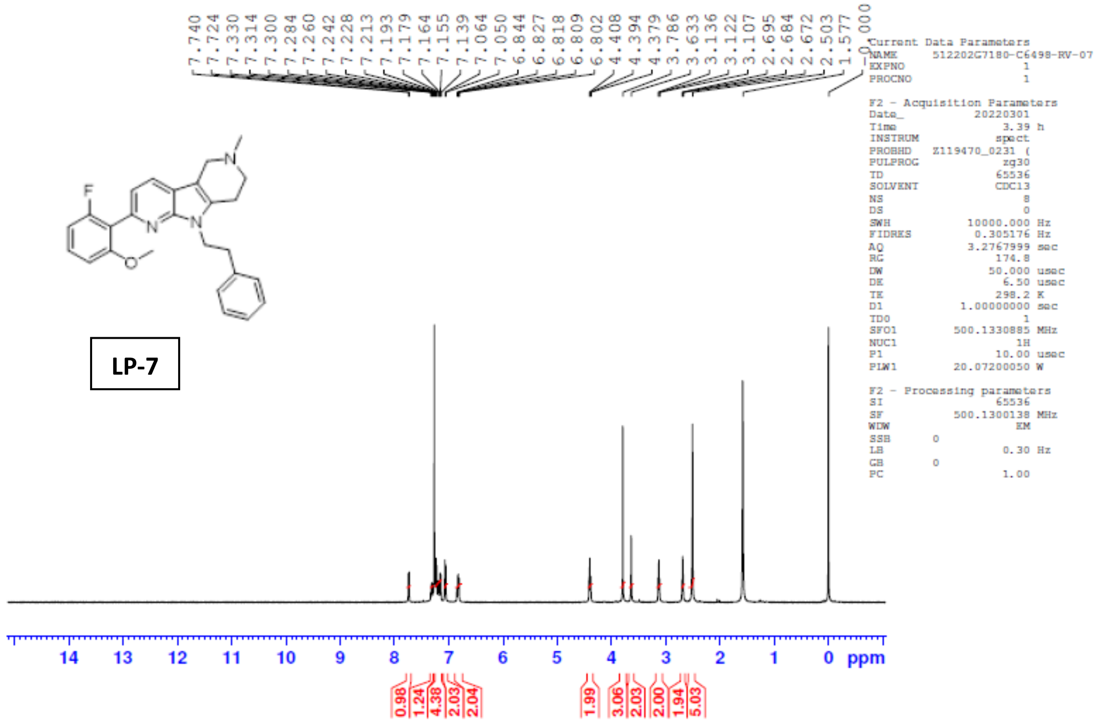

^{1}HNMR spectrum of 2-(2-Fluoro-6-methoxyphenyl)-6-methyl-9-phenethyl-6,7,8,9-tetrahydro-5H-pyrrolo[2,3-b:4,5-c']dipyridine (LP-7) in $CDCl_3$ (500 MHz)

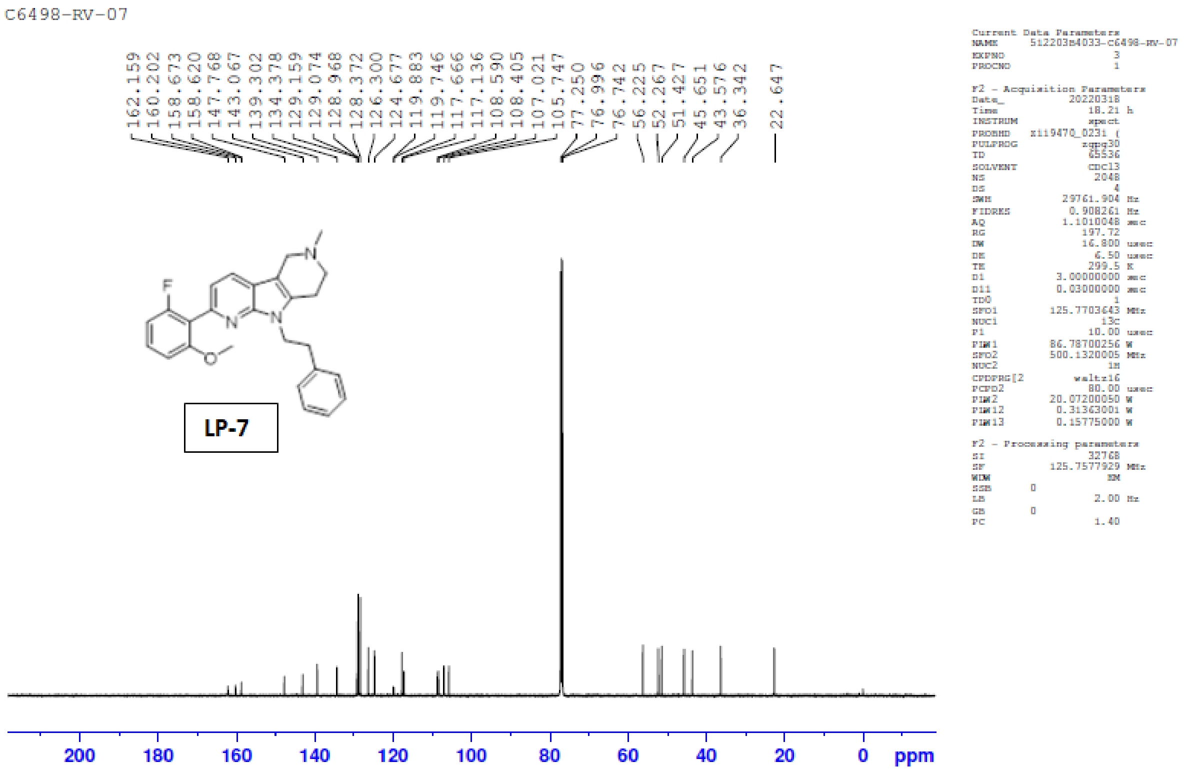

^{13}CNMR spectrum of 2-(2-Fluoro-6-methoxyphenyl)-6-methyl-9-phenethyl-6,7,8,9-tetrahydro-5H-pyrrolo[2,3-b:4,5-c']dipyridine (LP-7) in $CDCl_3$ (125 MHz)

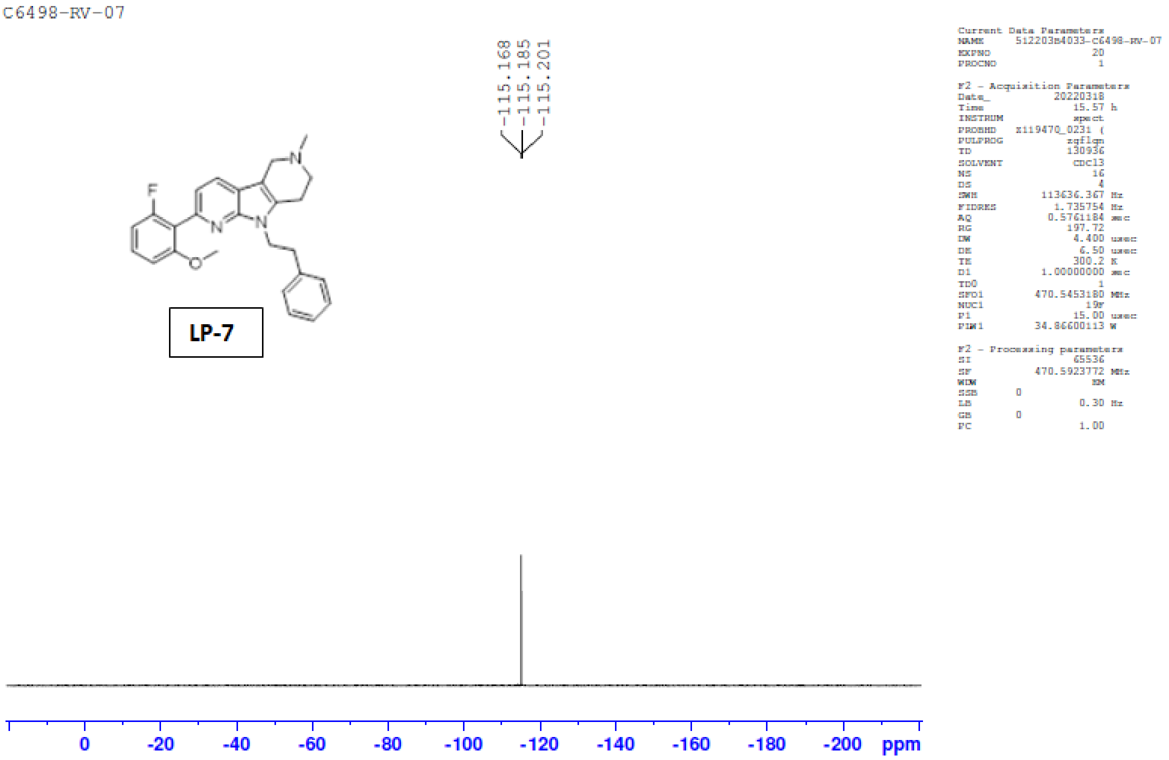

^{19}FNMR spectrum of 2-(2-Fluoro-6-methoxyphenyl)-6-methyl-9-phenethyl-6,7,8,9-tetrahydro-5H-pyrrolo[2,3-b:4,5-c']dipyridine (LP-7) in $CDCl_3$ (470 MHz)

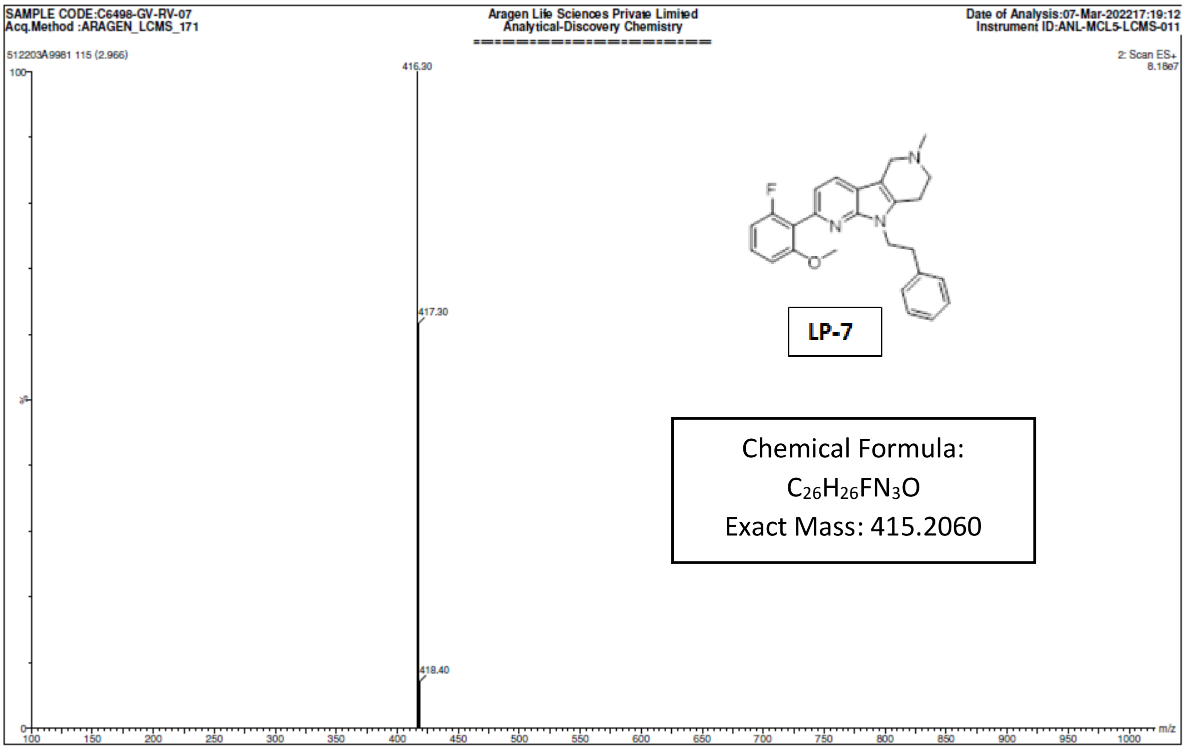

MASS spectrum of 2-(2-Fluoro-6-methoxyphenyl)-6-methyl-9-phenethyl-6,7,8,9-tetrahydro-5H-pyrrolo[2,3-b:4,5-c']dipyridine (LP-7)

Elemental Composition Report

Page 1

Single Mass Analysis

Tolerance = 500.0 PPM / DBE: min = -1.5, max = 50.0
Element prediction: Off
Number of isotope peaks used for i-FIT = 3

Monoisotopic Mass, Even Electron Ions
9 formula(e) evaluated with 1 results within limits (up to 50 best isotopic matches for each mass)
Elements Used:
C: 0-26 H: 0-27 N: 0-3 O: 0-1 F: 0-1

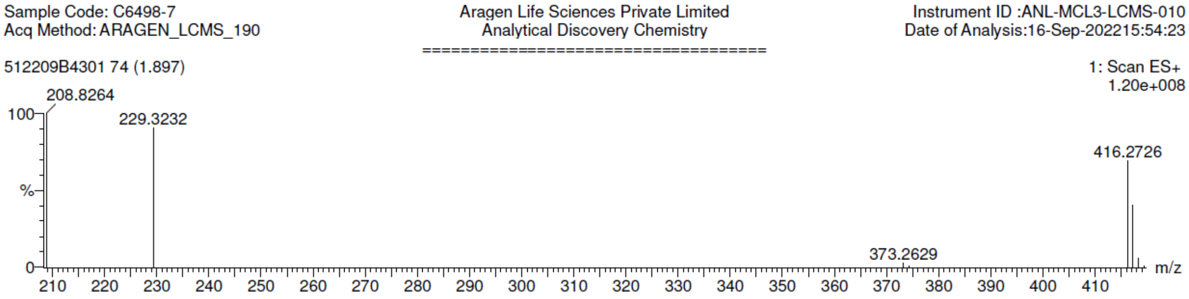

Mass	Calc. Mass	mDa	PPM	DBE	i-FIT	Norm	Conf(%)	Formula
Minimum:				-1.5				
Maximum:		5.0	500.0	50.0				
416.2150	416.2138	1.2	2.9	14.5	54.6	n/a	n/a	C26 H27 N3 O F

Elemental composition report of 2-(2-Fluoro-6-methoxyphenyl)-6-methyl-9-phenethyl-6,7,8,9-tetrahydro-5H-pyrrolo[2,3-b:4,5-c']dipyridine (LP-7)

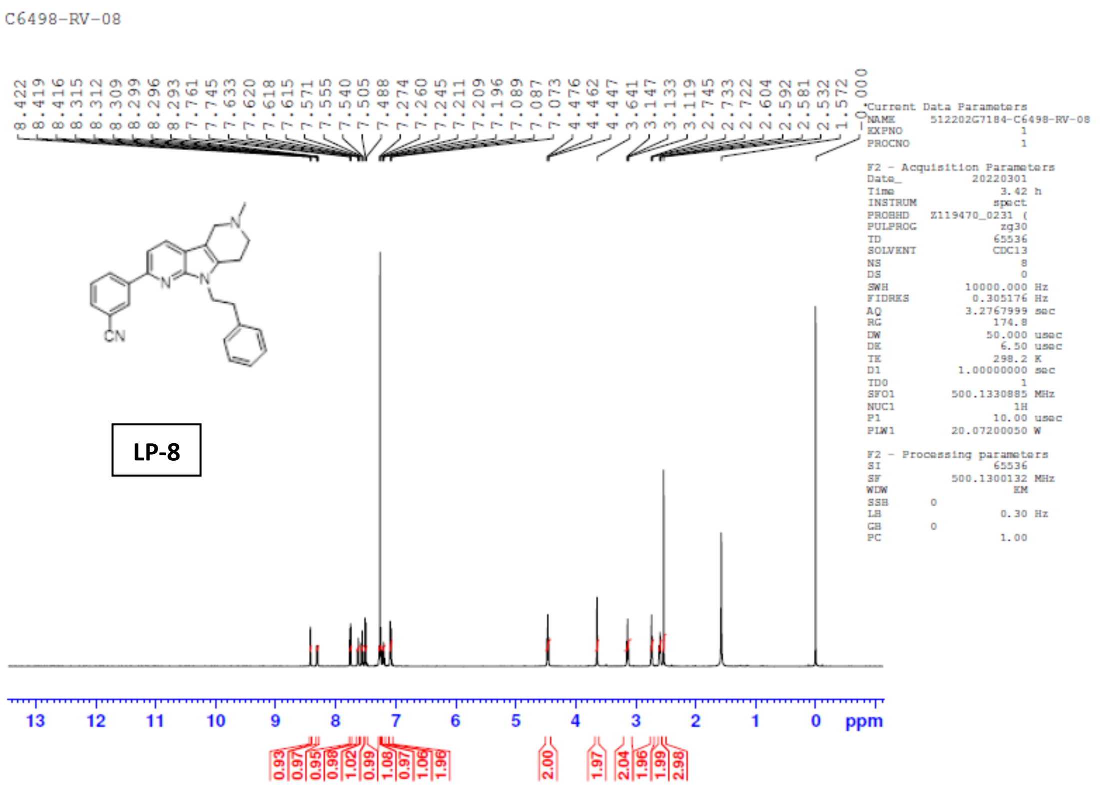

^{1}HNMR spectrum of 3-(6-Methyl-9-phenethyl-6,7,8,9-tetrahydro-5H-pyrrolo[2,3-b:4,5-c']dipyridin-2-yl)benzonitrile (LP-8) in $CDCl_3$ (500 MHz)

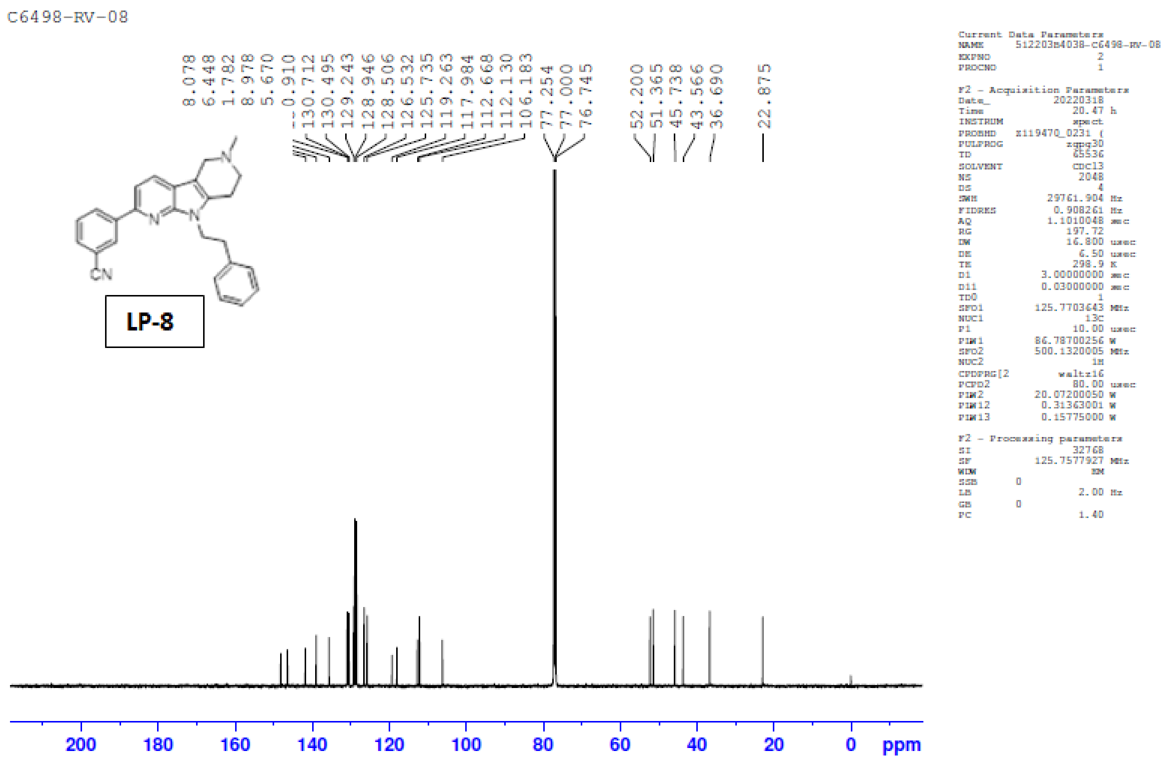

^{13}CNMR spectrum of 3-(6-Methyl-9-phenethyl-6,7,8,9-tetrahydro-5H-pyrrolo[2,3-b:4,5-c']dipyridin-2-yl)benzonitrile (LP-8) in $CDCl_3$ (125 MHz)

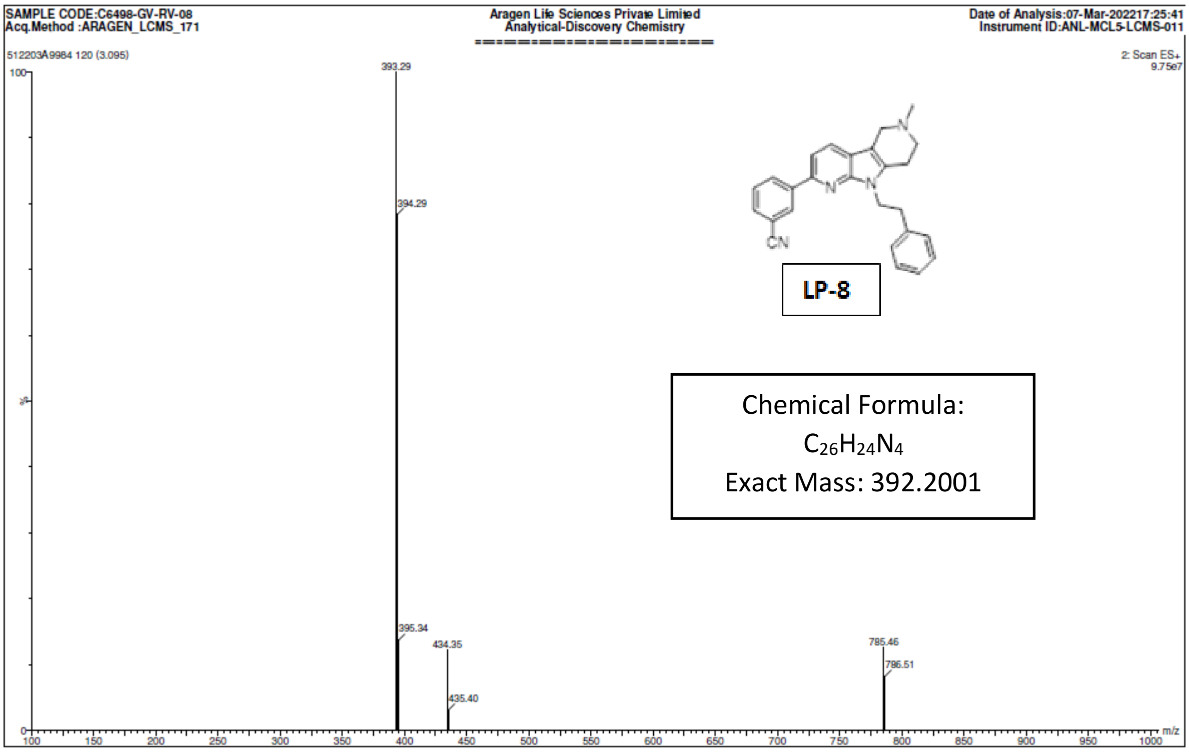

MASS spectrum of 3-(6-Methyl-9-phenethyl-6,7,8,9-tetrahydro-5H-pyrrolo[2,3-b:4,5-c']dipyridin-2-yl)benzonitrile (LP-8)

Elemental Composition Report

Page 1

Single Mass Analysis

Tolerance = 500.0 PPM / DBE: min = -1.5, max = 50.0
Element prediction: Off
Number of isotope peaks used for i-FIT = 3

Monoisotopic Mass, Even Electron Ions
3 formula(e) evaluated with 1 results within limits (up to 50 best isotopic matches for each mass)
Elements Used:
C: 0-26 H: 0-25 N: 0-4

Sample Code: C6498-8
Acq Method: ARAGEN_LCMS_190

Aragen Life Sciences Private Limited
Analytical Discovery Chemistry

Instrument ID :ANL-MCL3-LCMS-010
Date of Analysis:16-Sep-202216:00:30

512209B4302 74 (1.897)

1: Scan ES+
9.77e+007

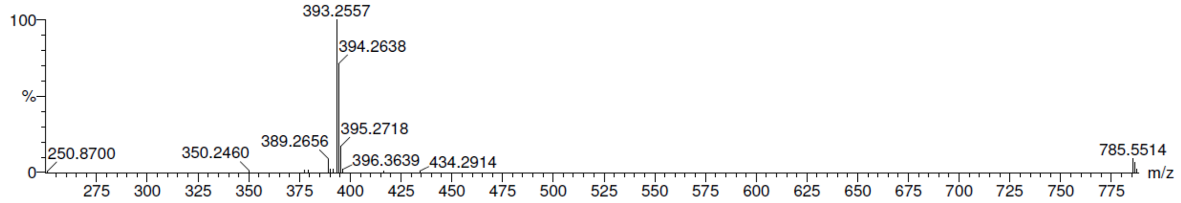

Minimum:				-1.5				
Maximum:		5.0	500.0	50.0				
Mass	Calc. Mass	mDa	PPM	DBE	i-FIT	Norm	Conf(%)	Formula
393.2065	393.2079	-1.4	-3.6	16.5	57.2	n/a	n/a	C26 H25 N4

Elemental composition report of 3-(6-Methyl-9-phenethyl-6,7,8,9-tetrahydro-5H-pyrrolo[2,3-b:4,5-c']dipyridin-2-yl)benzonitrile (LP-8)

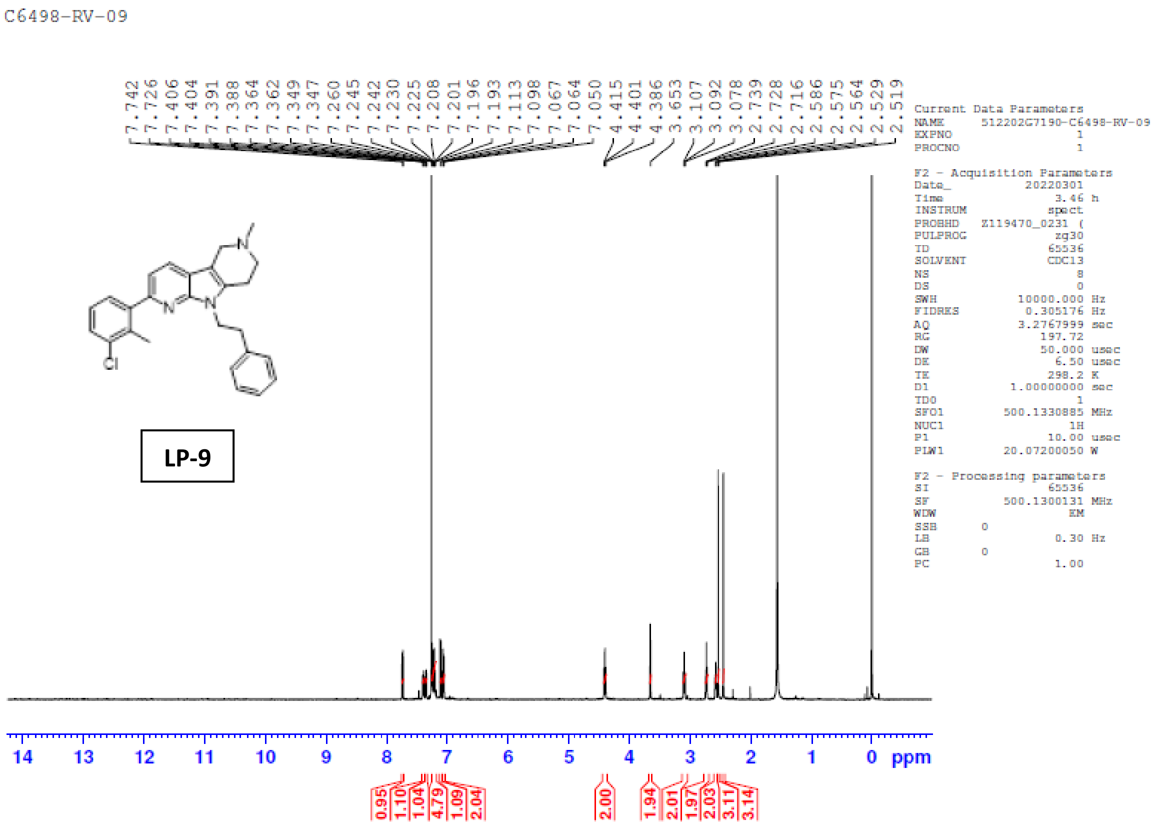

^{1}HNMR spectrum of 2-(3-Chloro-2-methylphenyl)-6-methyl-9-phenethyl-6,7,8,9-tetrahydro-5H-pyrrolo[2,3-b:4,5-c']dipyridine (LP-9) in $CDCl_3$ (500 MHz)

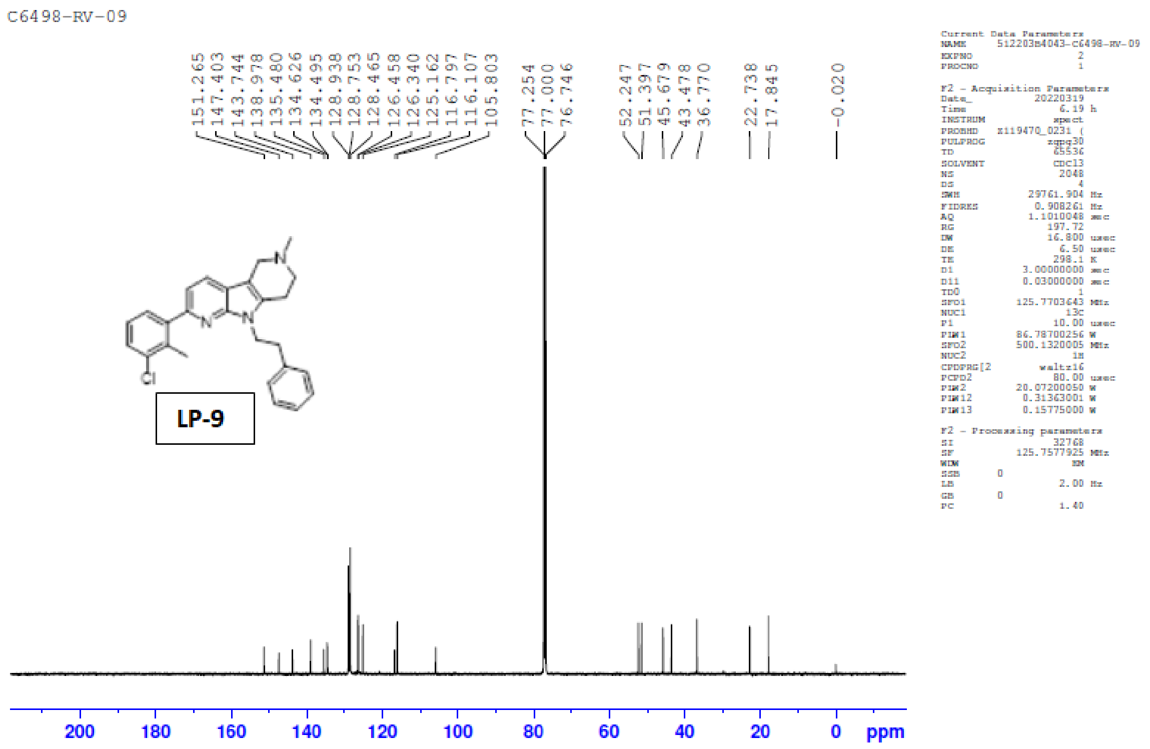

^{13}CNMR spectrum of 2-(3-Chloro-2-methylphenyl)-6-methyl-9-phenethyl-6,7,8,9-tetrahydro-5H-pyrrolo[2,3-b:4,5-c']dipyridine (LP-9) in $CDCl_3$ (125 MHz)

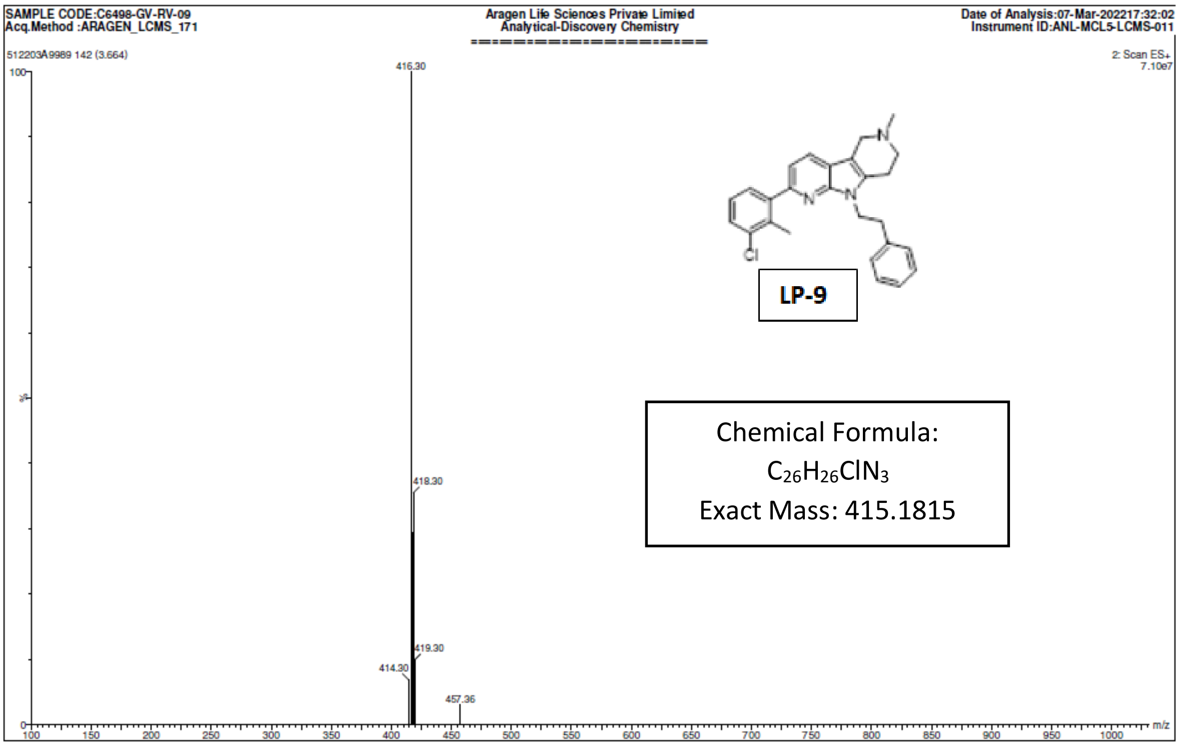

MASS spectrum of 2-(3-Chloro-2-methylphenyl)-6-methyl-9-phenethyl-6,7,8,9-tetrahydro-5H-pyrrolo[2,3-b:4,5-c']dipyridine (LP-9)

Elemental Composition Report **Page 1**

Single Mass Analysis
Tolerance = 500.0 PPM / DBE: min = -1.5, max = 50.0
Element prediction: Off
Number of isotope peaks used for i-FIT = 3

Monoisotopic Mass, Even Electron Ions
5 formula(e) evaluated with 1 results within limits (up to 50 best isotopic matches for each mass)
Elements Used:
C: 0-26 H: 0-27 N: 0-3 Cl: 0-1

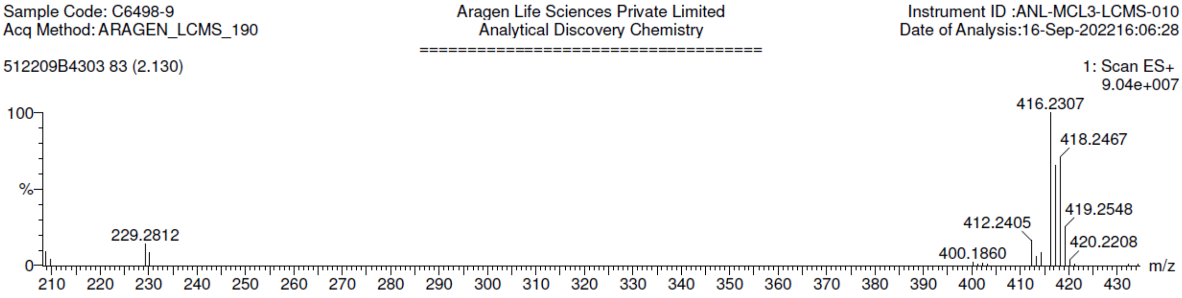

Minimum:				-1.5				
Maximum:		5.0	500.0	50.0				
Mass	Calc. Mass	mDa	PPM	DBE	i-FIT	Norm	Conf(%)	Formula
416.1877	416.1894	-1.7	-4.1	14.5	63.8	n/a	n/a	C26 H27 N3 Cl

Elemental composition report of 2-(3-Chloro-2-methylphenyl)-6-methyl-9-phenethyl-6,7,8,9-tetrahydro-5H-pyrrolo[2,3-b:4,5-c']dipyridine (LP-9)

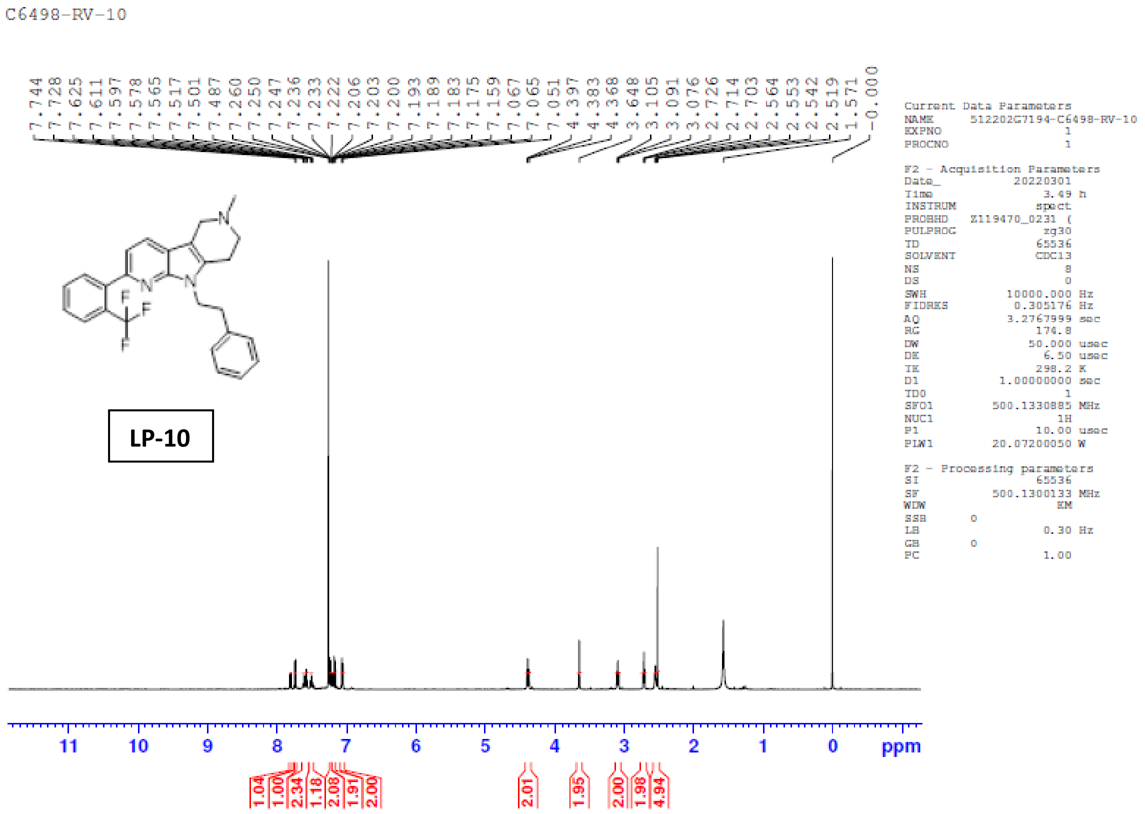

^{1}HNMR spectrum of 6-Methyl-9-phenethyl-2-(2-(trifluoromethyl)phenyl)-6,7,8,9-tetrahydro-5H-pyrrolo[2,3-b:4,5-c']dipyridine (LP-10) in $CDCl_3$ (500 MHz)

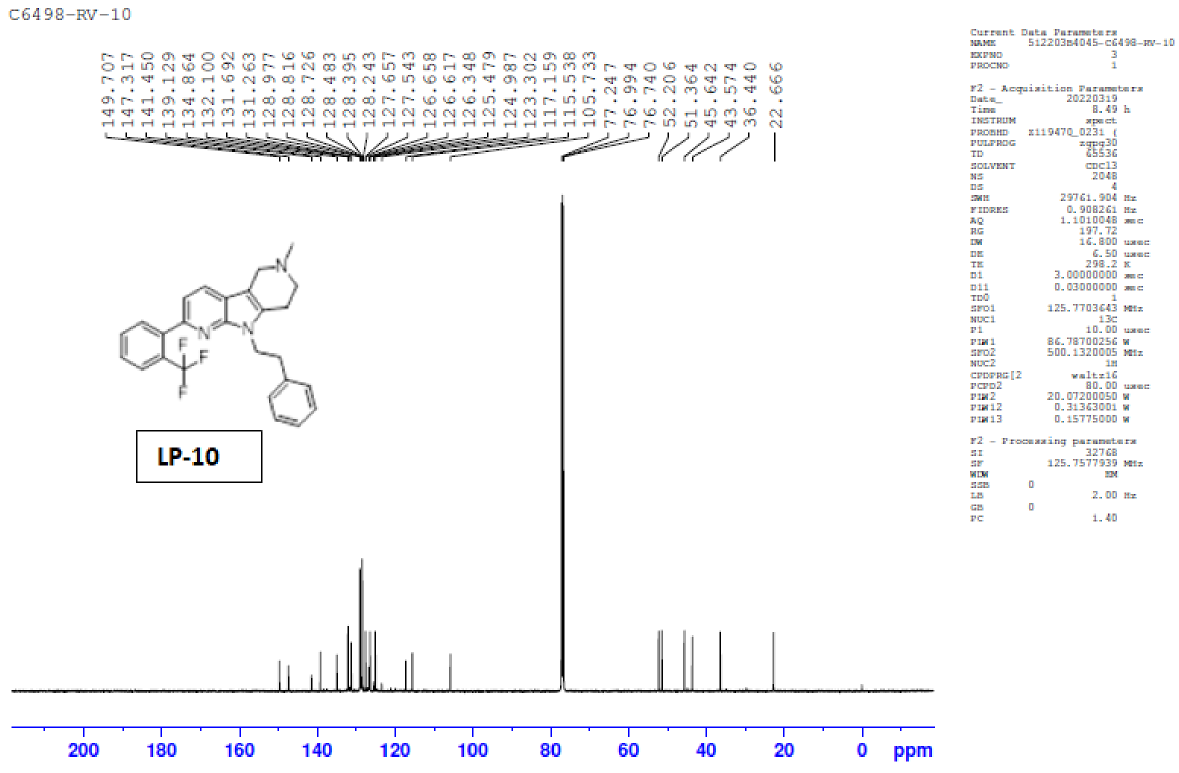

^{13}CNMR spectrum of 6-Methyl-9-phenethyl-2-(2-(trifluoromethyl)phenyl)-6,7,8,9-tetrahydro-5H-pyrrolo[2,3-b:4,5-c']dipyridine (LP-10) in $CDCl_3$ (125 MHz)

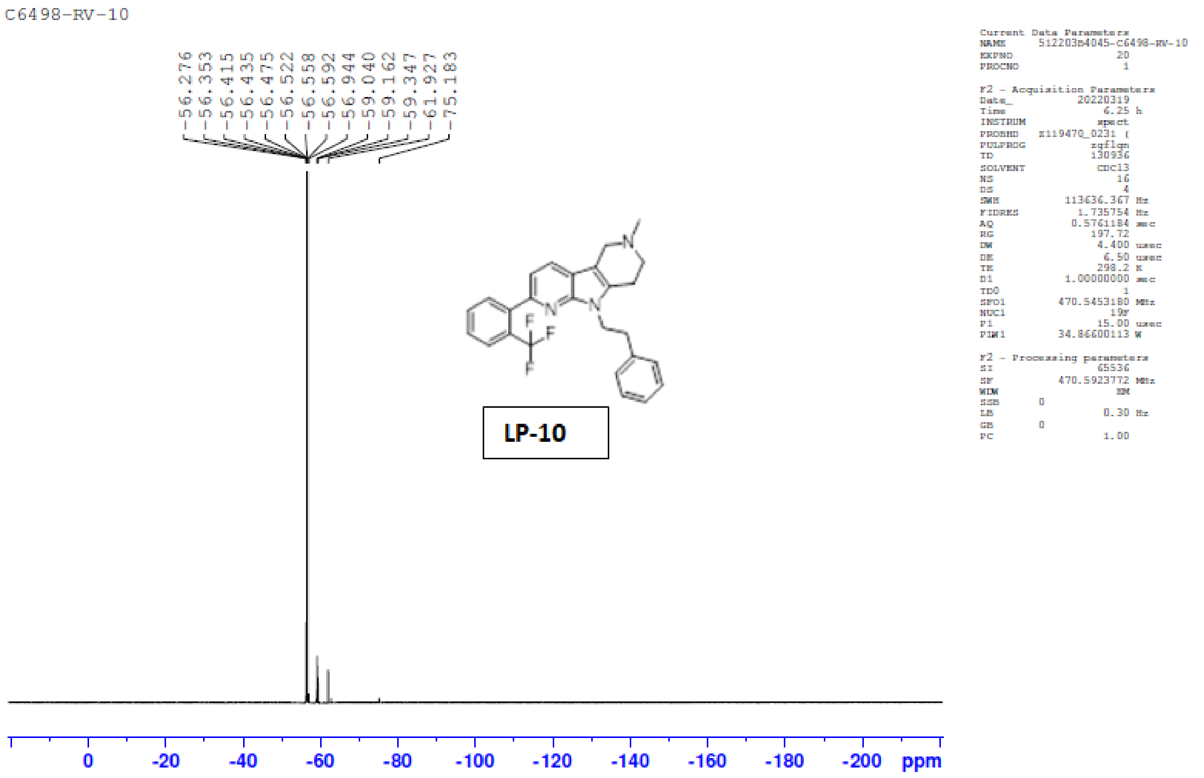

^{19}FNMR spectrum of 6-Methyl-9-phenethyl-2-(2-(trifluoromethyl)phenyl)-6,7,8,9-tetrahydro-5H-pyrrolo[2,3-b:4,5-c']dipyridine (LP-10) in $CDCl_3$ (470 MHz)

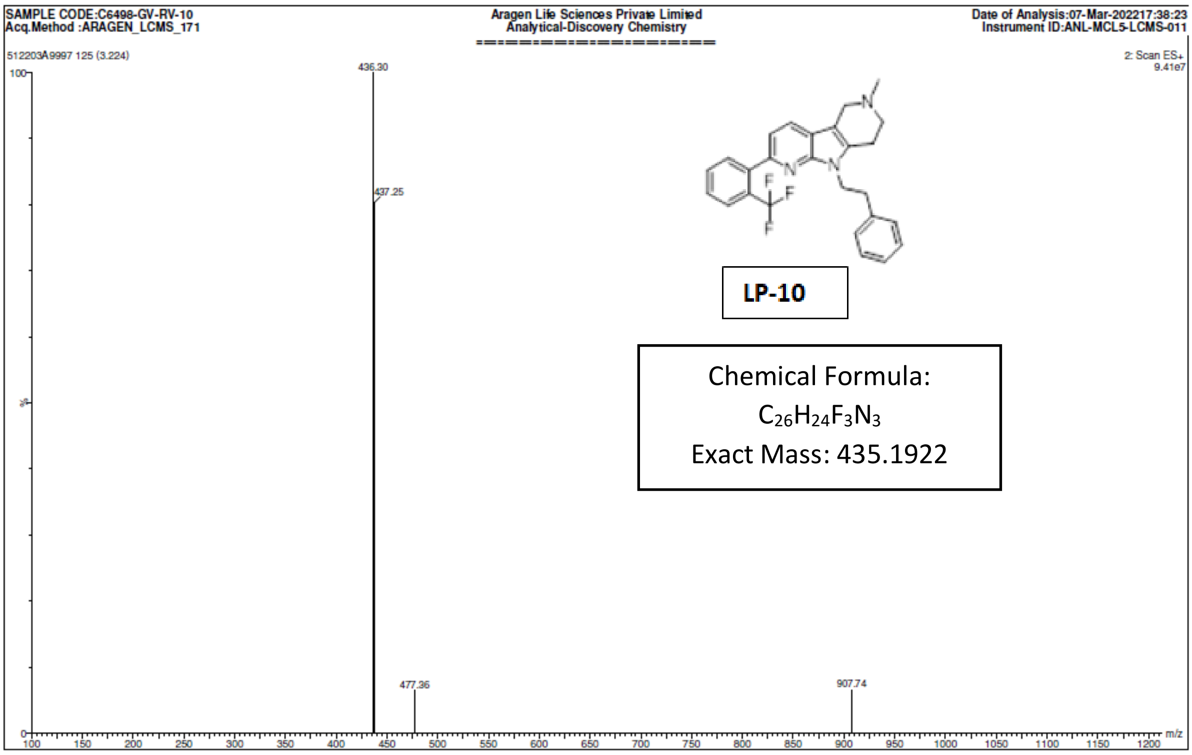

MASS spectrum of 6-Methyl-9-phenethyl-2-(2-(trifluoromethyl)phenyl)-6,7,8,9-tetrahydro-5H-pyrrolo[2,3-b:4,5-c']dipyridine (LP-10)

Elemental Composition Report

Single Mass Analysis

Tolerance = 500.0 PPM / DBE: min = -1.5, max = 50.0
Element prediction: Off
Number of isotope peaks used for i-FIT = 3

Monoisotopic Mass, Even Electron Ions
11 formula(e) evaluated with 1 results within limits (up to 50 best isotopic matches for each mass)
Elements Used:
C: 0-26 H: 0-25 N: 0-3 F: 0-3

Sample Code: C6498-10
Acq Method: ARAGEN_LCMS_190

Aragen Life Sciences Private Limited
Analytical Discovery Chemistry
==================================

Instrument ID :ANL-MCL3-LCMS-010
Date of Analysis:16-Sep-202216:12:26

512209B4304 78 (2.001)

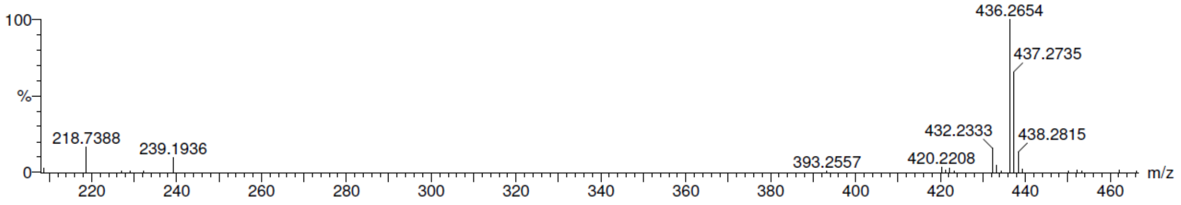

Minimum:				-1.5				
Maximum:		5.0	500.0	50.0				
Mass	Calc. Mass	mDa	PPM	DBE	i-FIT	Norm	Conf(%)	Formula
436.2031	436.2001	3.0	6.9	14.5	56.3	n/a	n/a	C26 H25 N3 F3

Elemental composition report of 6-Methyl-9-phenethyl-2-(2-(trifluoromethyl)phenyl)-6,7,8,9-tetrahydro-5H-pyrrolo[2,3-b:4,5-c']dipyridine (LP-10)

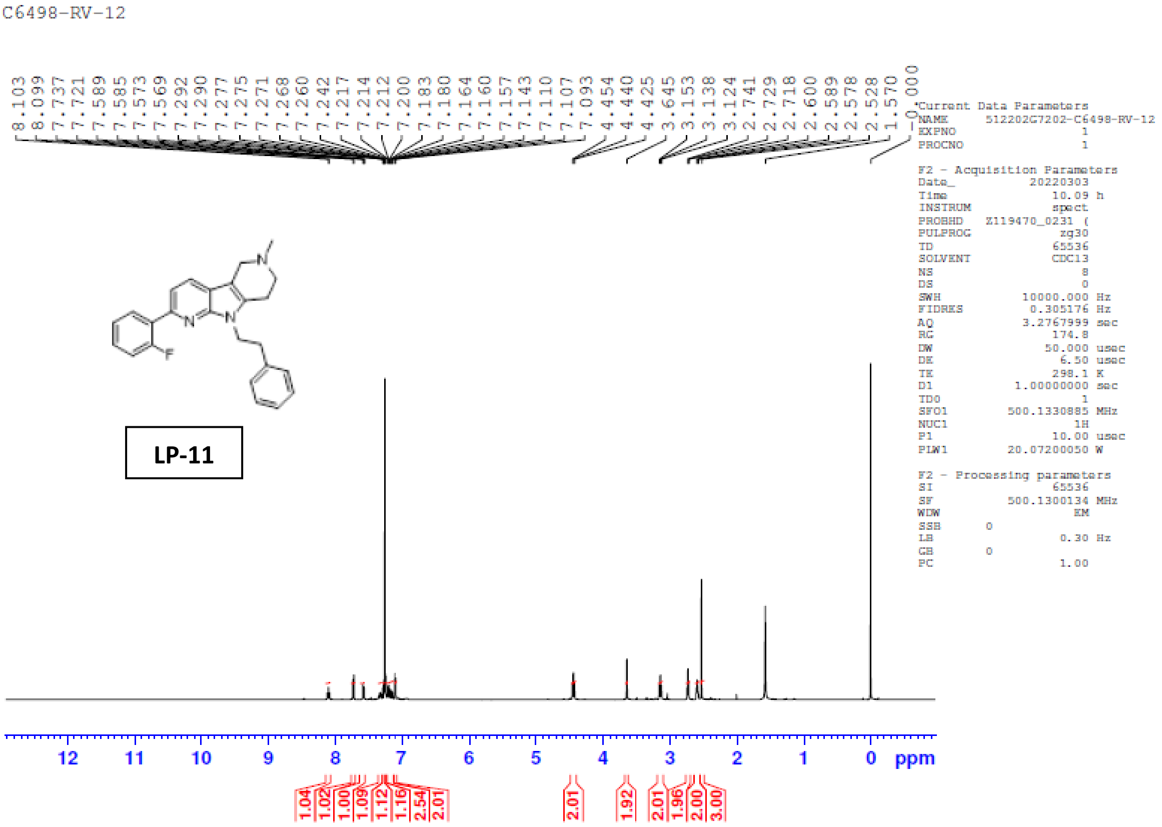

^{1}HNMR spectrum of 2-(2-Fluorophenyl)-6-methyl-9-phenethyl-6,7,8,9-tetrahydro-5H-pyrrolo[2,3-b:4,5-c']dipyridine: (LP-11) in $CDCl_3$ (500 MHz)

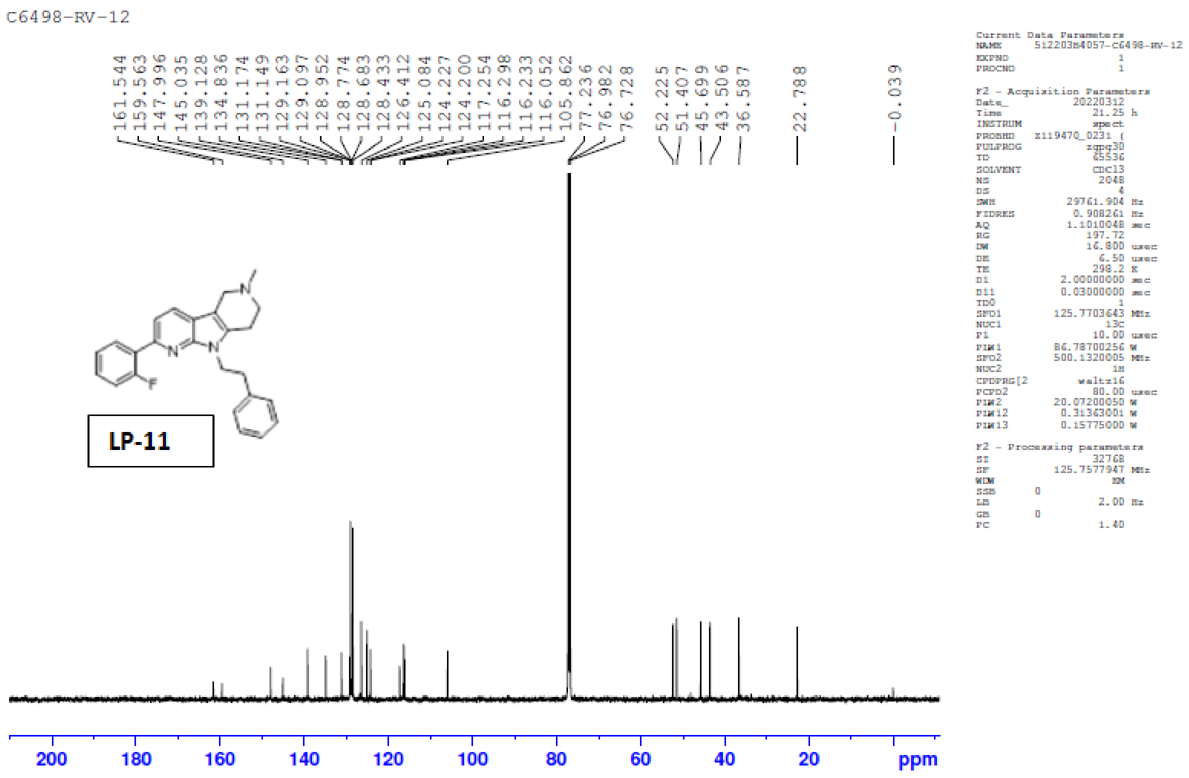

^{13}CNMR spectrum of 2-(2-Fluorophenyl)-6-methyl-9-phenethyl-6,7,8,9-tetrahydro-5H-pyrrolo[2,3-b:4,5-c']dipyridine (LP-11) in $CDCl_3$ (125 MHz)

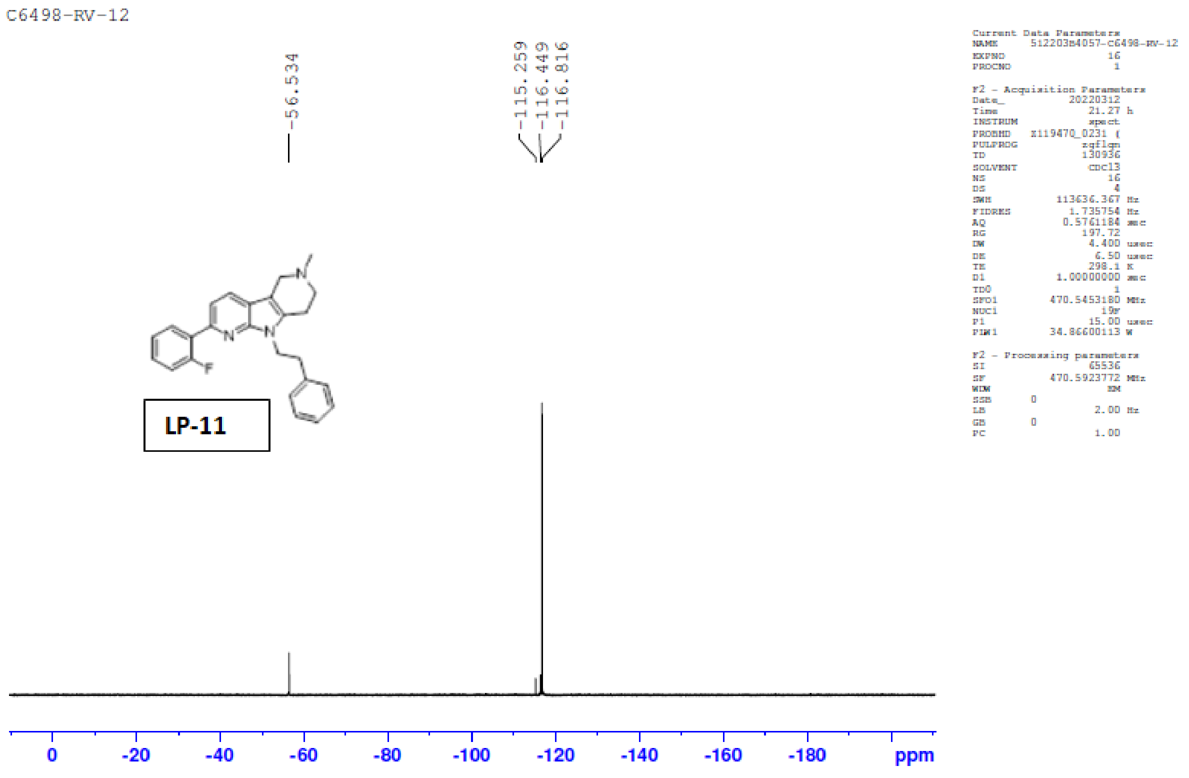

^{19}FNMR spectrum of 2-(2-Fluorophenyl)-6-methyl-9-phenethyl-6,7,8,9-tetrahydro-5H-pyrrolo[2,3-b:4,5-c']dipyridine (LP-11) in $CDCl_3$ (470 MHz)

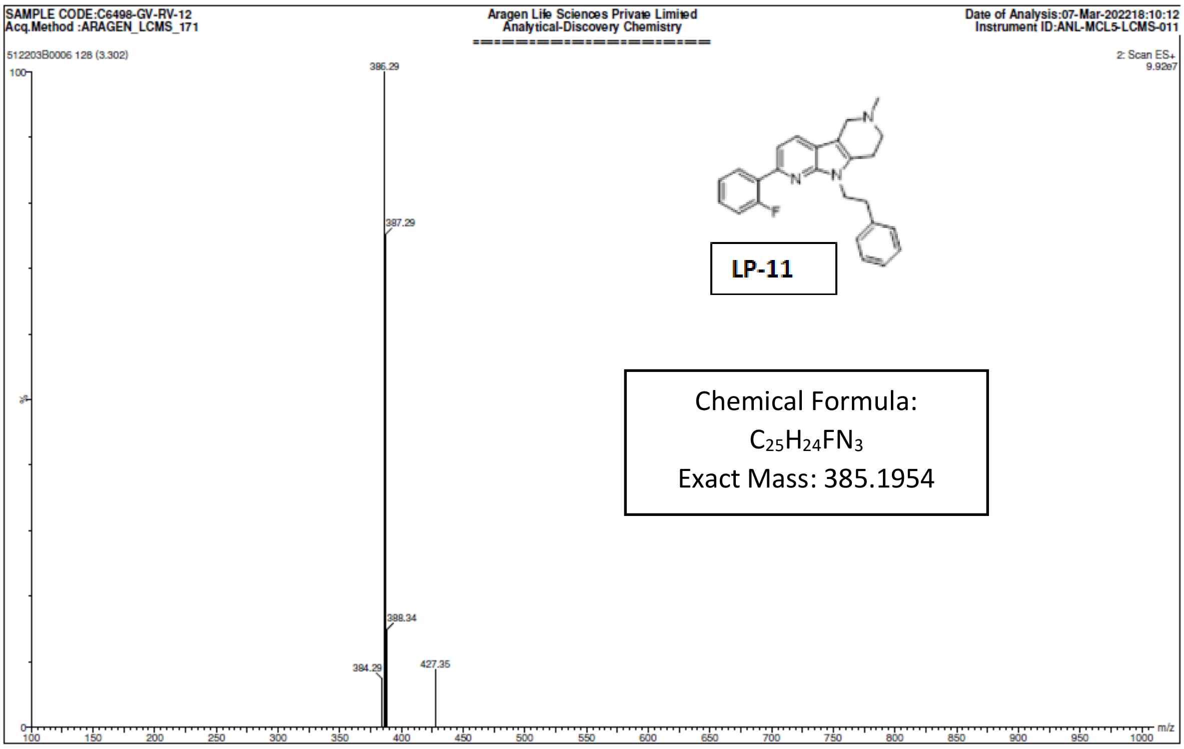

MASS spectrum of 2-(2-Fluorophenyl)-6-methyl-9-phenethyl-6,7,8,9-tetrahydro-5H-pyrrolo[2,3-b:4,5-c']dipyridine (LP-11)

Elemental Composition Report **Page 1**

Single Mass Analysis
Tolerance = 820.0 PPM / DBE: min = -1.5, max = 50.0
Element prediction: Off
Number of isotope peaks used for i-FIT = 3

Monoisotopic Mass, Even Electron Ions
4 formula(e) evaluated with 1 results within limits (up to 50 best isotopic matches for each mass)
Elements Used:
C: 0-25 H: 0-25 N: 0-3 F: 0-1

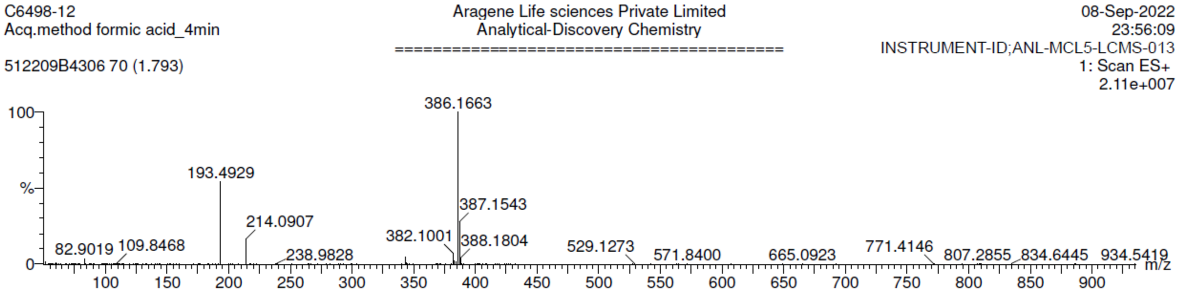

Minimum:				-1.5				
Maximum:		5.0	820.0	50.0				
Mass	Calc. Mass	mDa	PPM	DBE	i-FIT	Norm	Conf(%)	Formula
386.2001	386.2033	-3.2	-8.3	14.5	39.8	n/a	n/a	C25 H25 N3 F

Elemental composition report of 2-(2-Fluorophenyl)-6-methyl-9-phenethyl-6,7,8,9-tetrahydro-5H-pyrrolo[2,3-b:4,5-c']dipyridine (LP-11)

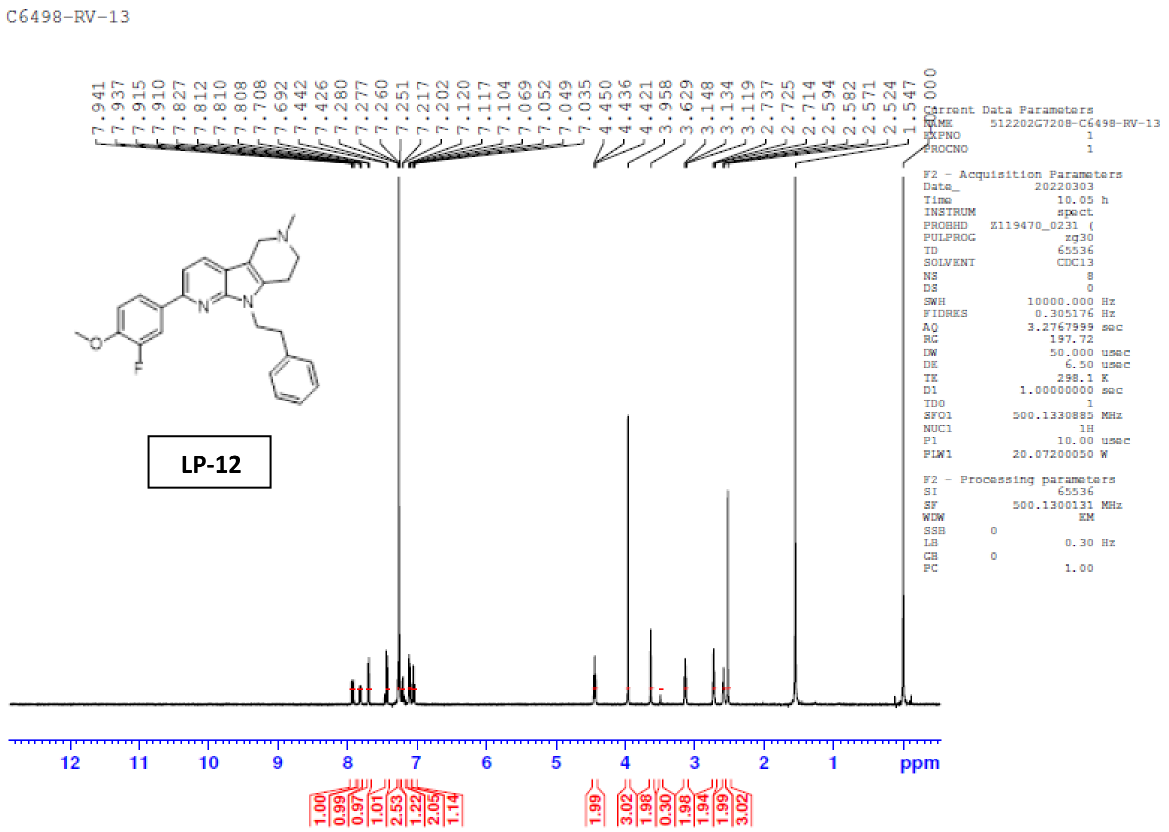

^{1}HNMR spectrum of 2-(3-Fluoro-4-methoxyphenyl)-6-methyl-9-phenethyl-6,7,8,9-tetrahydro-5H-pyrrolo[2,3-b:4,5-c']dipyridine (LP-12) in $CDCl_3$ (500 MHz)

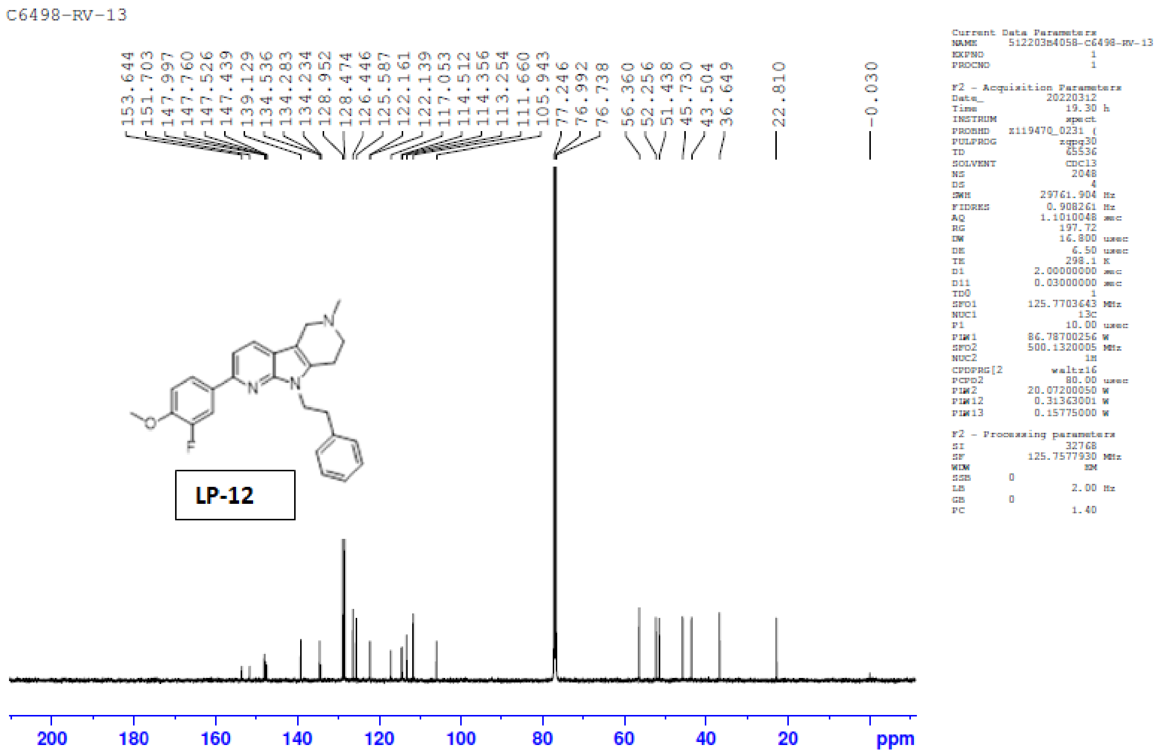

^{13}CNMR spectrum of 2-(3-Fluoro-4-methoxyphenyl)-6-methyl-9-phenethyl-6,7,8,9-tetrahydro-5H-pyrrolo[2,3-b:4,5-c']dipyridine (LP-12) in $CDCl_3$ (125 MHz)

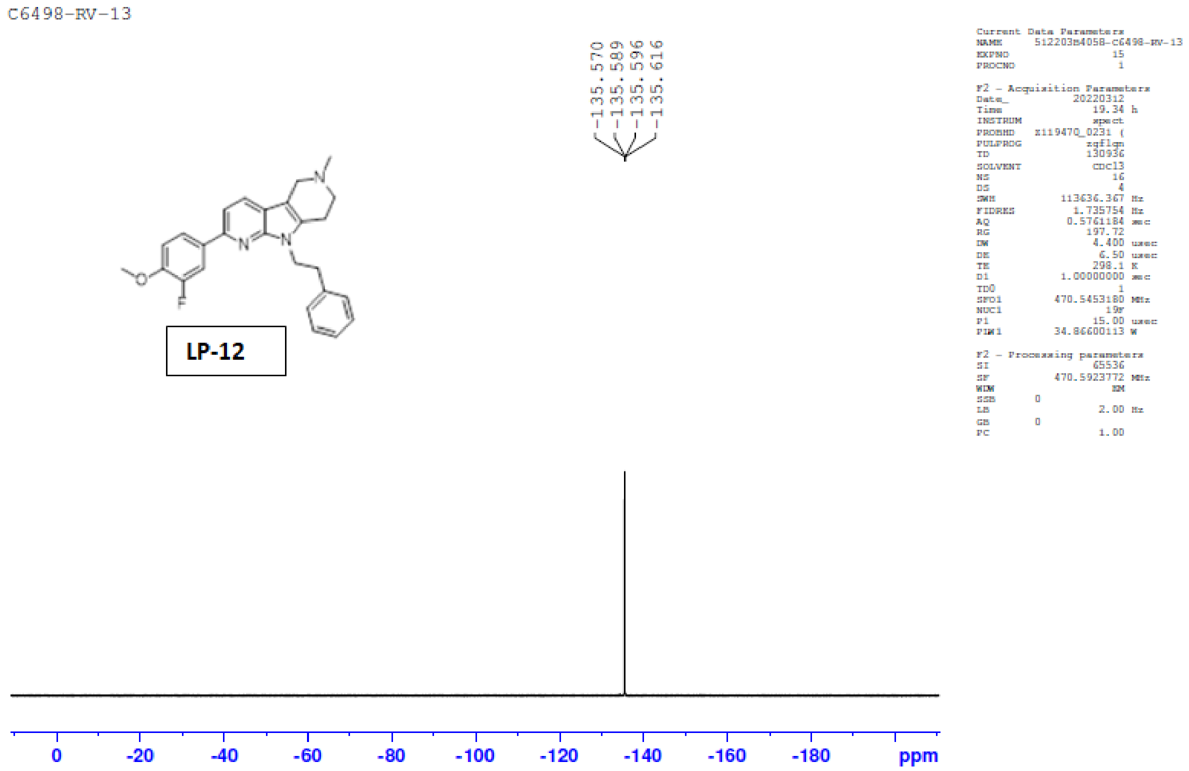

^{19}FNMR spectrum of 2-(3-Fluoro-4-methoxyphenyl)-6-methyl-9-phenethyl-6,7,8,9-tetrahydro-5H-pyrrolo[2,3-b:4,5-c']dipyridine (LP-12) in $CDCl_3$ (470 MHz)

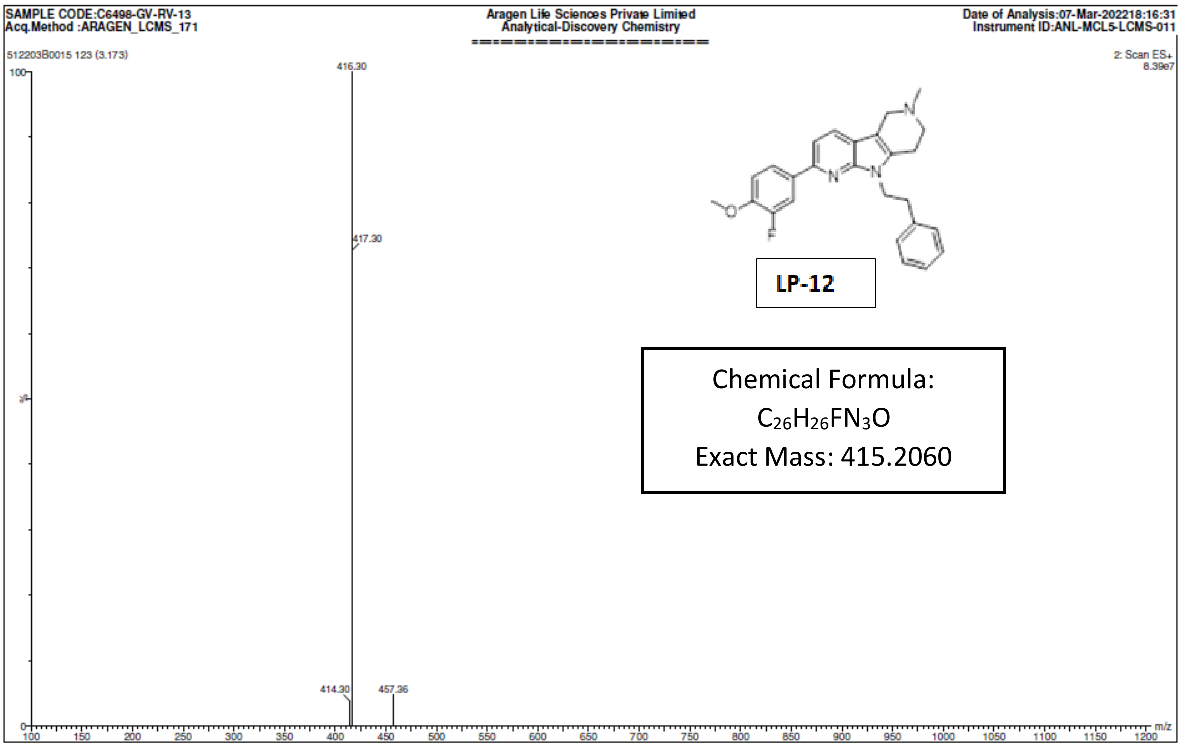

MASS spectrum of 2-(3-Fluoro-4-methoxyphenyl)-6-methyl-9-phenethyl-6,7,8,9-tetrahydro-5H-pyrrolo[2,3-b:4,5-c']dipyridine (LP-12)

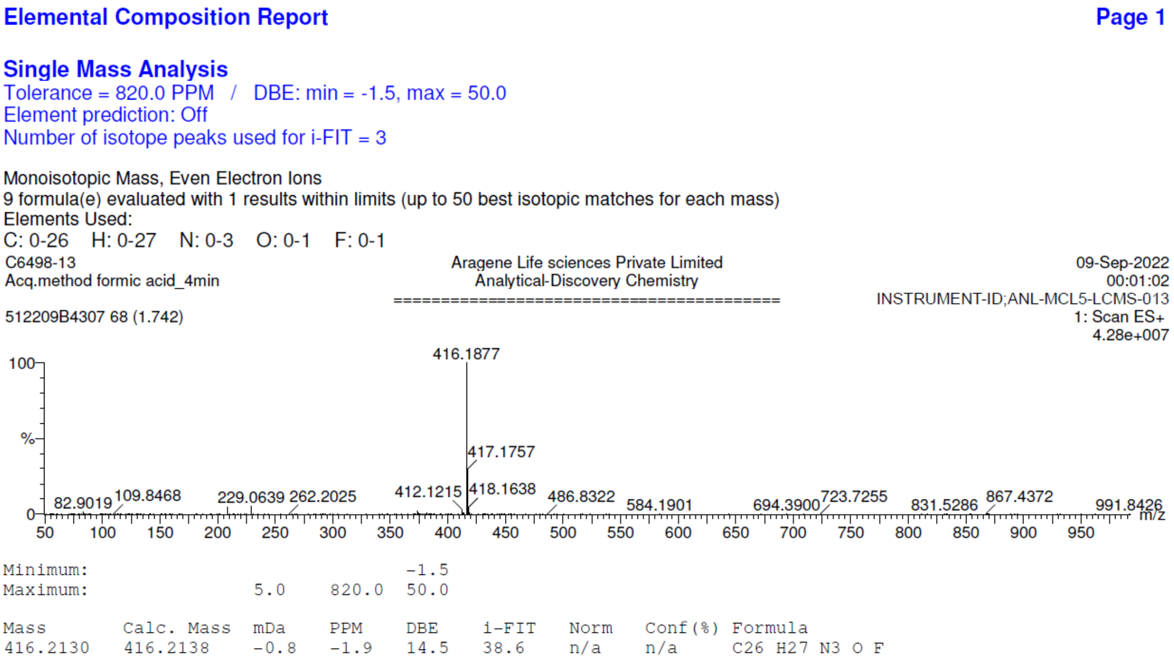

Elemental Composition Report Page 1

Single Mass Analysis
Tolerance = 820.0 PPM / DBE: min = -1.5, max = 50.0
Element prediction: Off
Number of isotope peaks used for i-FIT = 3

Monoisotopic Mass, Even Electron Ions
9 formula(e) evaluated with 1 results within limits (up to 50 best isotopic matches for each mass)
Elements Used:
C: 0-26 H: 0-27 N: 0-3 O: 0-1 F: 0-1
C6498-13
Acq.method formic acid_4min
Aragene Life sciences Private Limited
Analytical-Discovery Chemistry
09-Sep-2022
00:01:02
INSTRUMENT-ID;ANL-MCL5-LCMS-013
512209B4307 68 (1.742)
1: Scan ES+
4.28e+007

Minimum:				-1.5				
Maximum:		5.0	820.0	50.0				
Mass	Calc. Mass	mDa	PPM	DBE	i-FIT	Norm	Conf(%)	Formula
416.2130	416.2138	-0.8	-1.9	14.5	38.6	n/a	n/a	C26 H27 N3 O F

Elemental composition report of 2-(3-Fluoro-4-methoxyphenyl)-6-methyl-9-phenethyl-6,7,8,9-tetrahydro-5H-pyrrolo[2,3-b:4,5-c']dipyridine (LP-12)

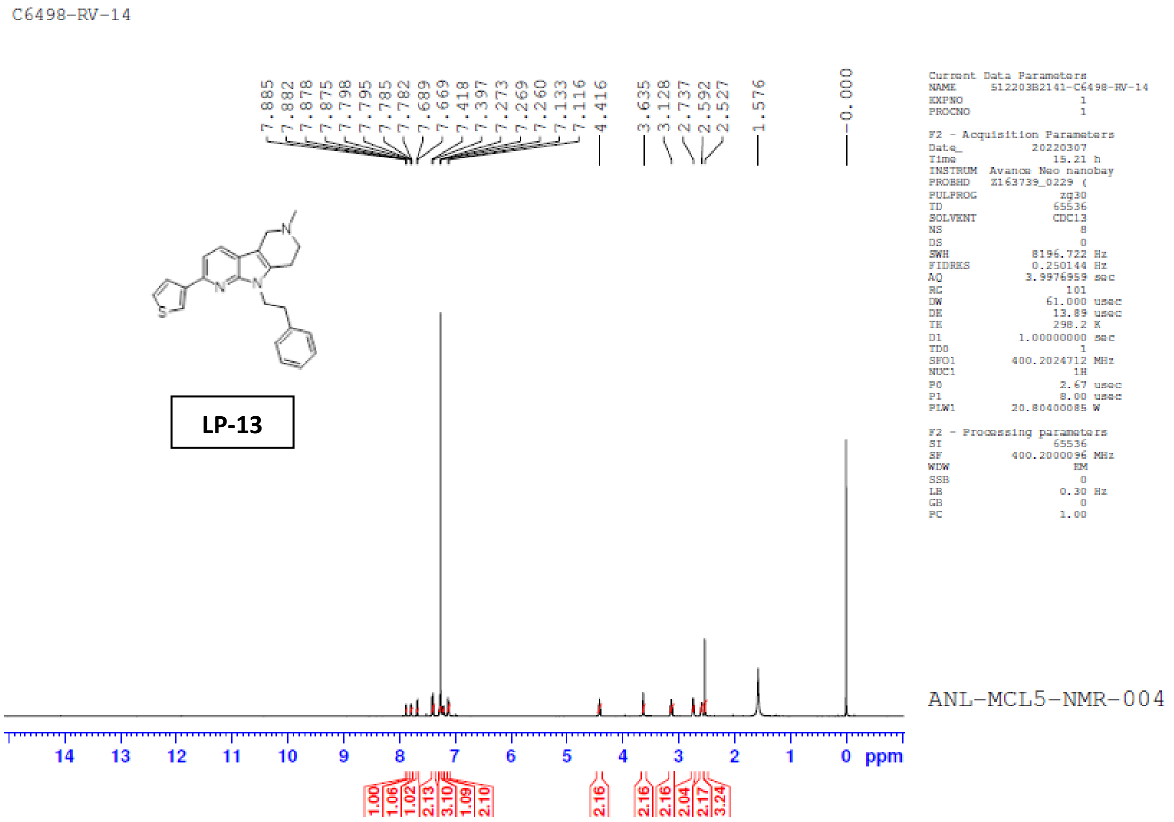

^{1}HNMR spectrum of 6-Methyl-9-phenethyl-2-(thiophen-3-yl)-6,7,8,9-tetrahydro-5H-pyrrolo[2,3-b:4,5-c']dipyridine (LP-13) in $CDCl_3$ (400 MHz)

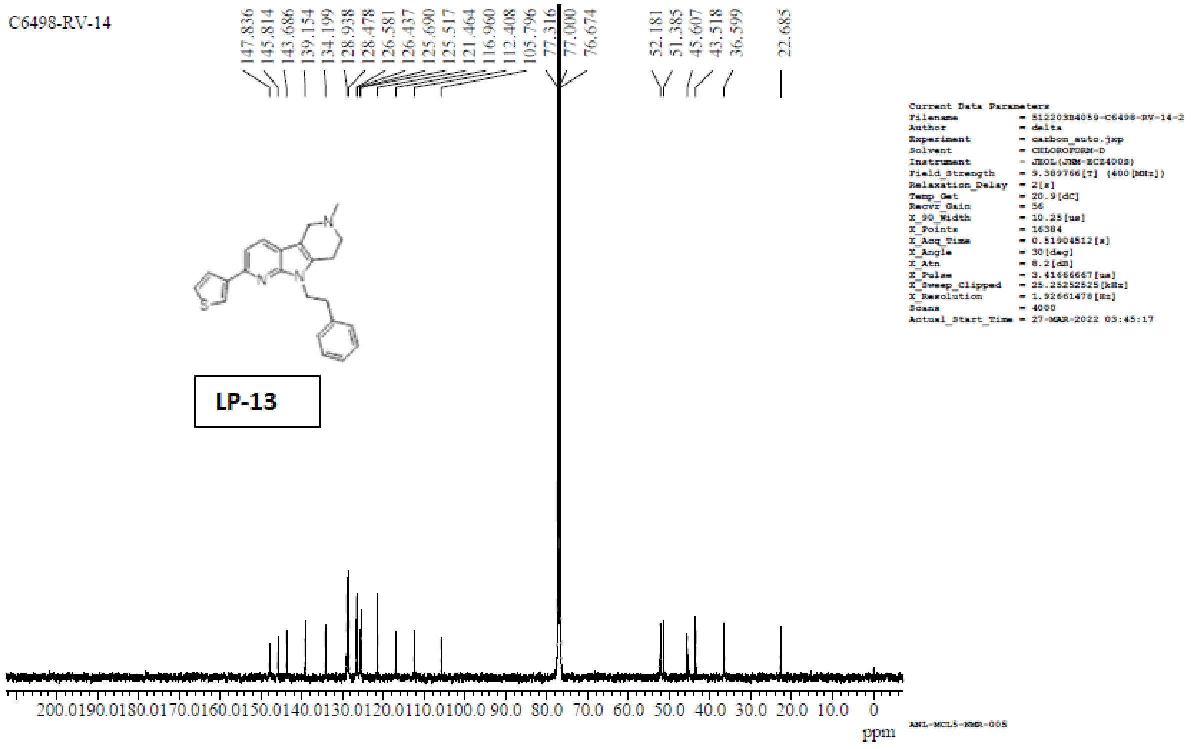

^{13}CNMR spectrum of 6-Methyl-9-phenethyl-2-(thiophen-3-yl)-6,7,8,9-tetrahydro-5H-pyrrolo[2,3-b:4,5-c']dipyridine (LP-13) in $CDCl_3$ (100 MHz)

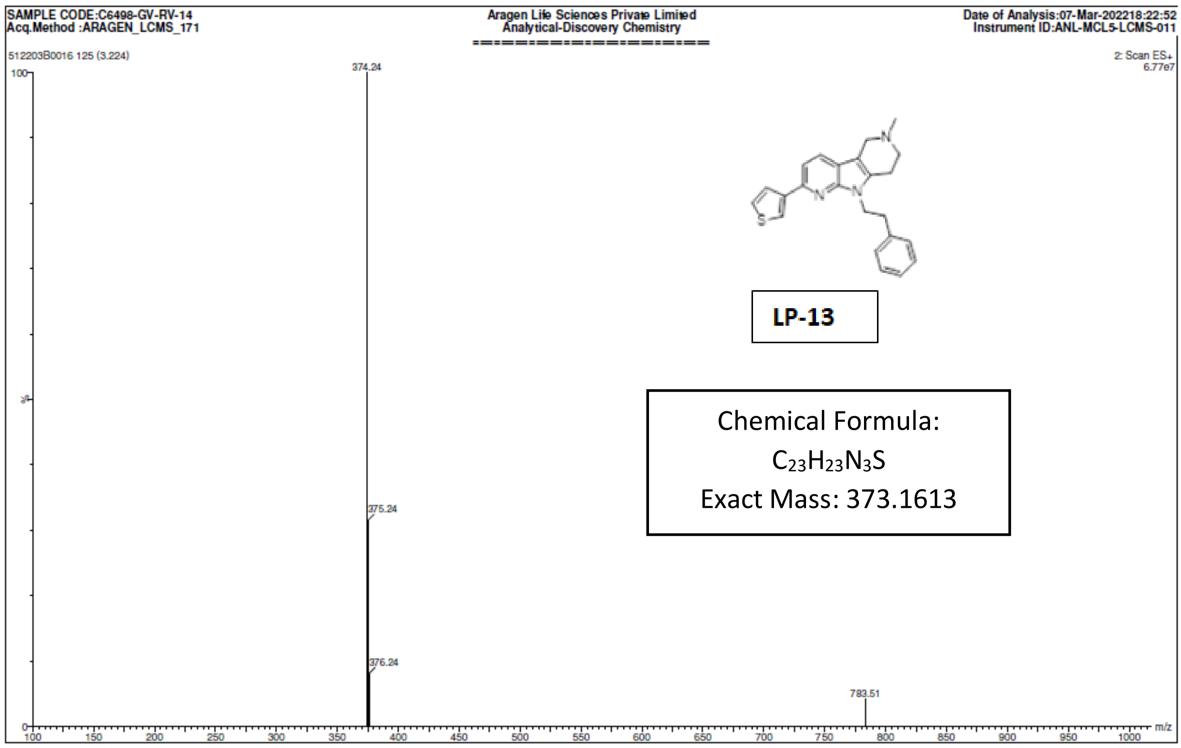

MASS spectrum of 6-Methyl-9-phenethyl-2-(thiophen-3-yl)-6,7,8,9-tetrahydro-5H-pyrrolo[2,3-b:4,5-c']dipyridine (LP-13)

Elemental Composition Report

Page 1

Single Mass Analysis

Tolerance = 820.0 PPM / DBE: min = -1.5, max = 50.0
Element prediction: Off
Number of isotope peaks used for i-FIT = 3

Monoisotopic Mass, Even Electron Ions
5 formula(e) evaluated with 1 results within limits (up to 50 best isotopic matches for each mass)
Elements Used:
C: 0-23 H: 0-24 N: 0-3 S: 0-1

C6498-14 | Aragene Life sciences Private Limited | 09-Sep-2022
Acq.method formic acid_4min | Analytical-Discovery Chemistry | 00:05:55
INSTRUMENT-ID;ANL-MCL5-LCMS-013
512209B4308 70 (1.793) | | 1: Scan ES+ 1.32e+007

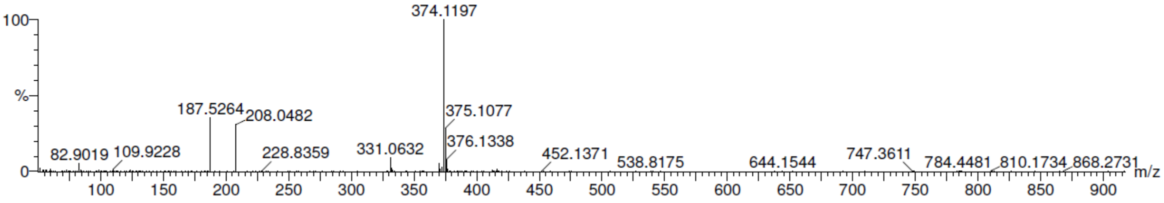

Minimum:				-1.5				
Maximum:		5.0	820.0	50.0				
Mass	Calc. Mass	mDa	PPM	DBE	i-FIT	Norm	Conf(%)	Formula
374.1681	374.1691	-1.0	-2.7	13.5	40.5	n/a	n/a	C23 H24 N3 S

Elemental composition report of 6-Methyl-9-phenethyl-2-(thiophen-3-yl)-6,7,8,9-tetrahydro-5H-pyrrolo[2,3-b:4,5-c']dipyridine (LP-13)

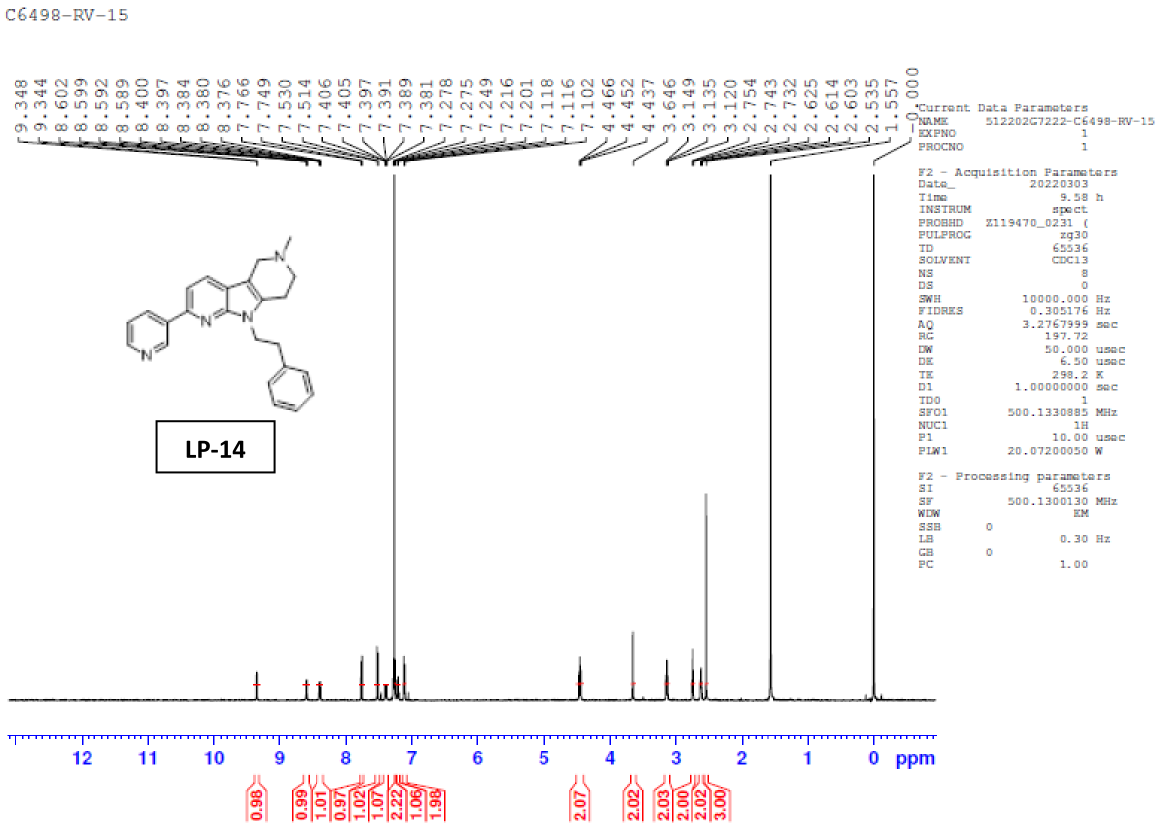

^{1}HNMR spectrum of 6-Methyl-9-phenethyl-2-(pyridin-3-yl)-6,7,8,9-tetrahydro-5H-pyrrolo[2,3-b:4,5-c']dipyridine (LP-14) in $CDCl_3$ (500 MHz)

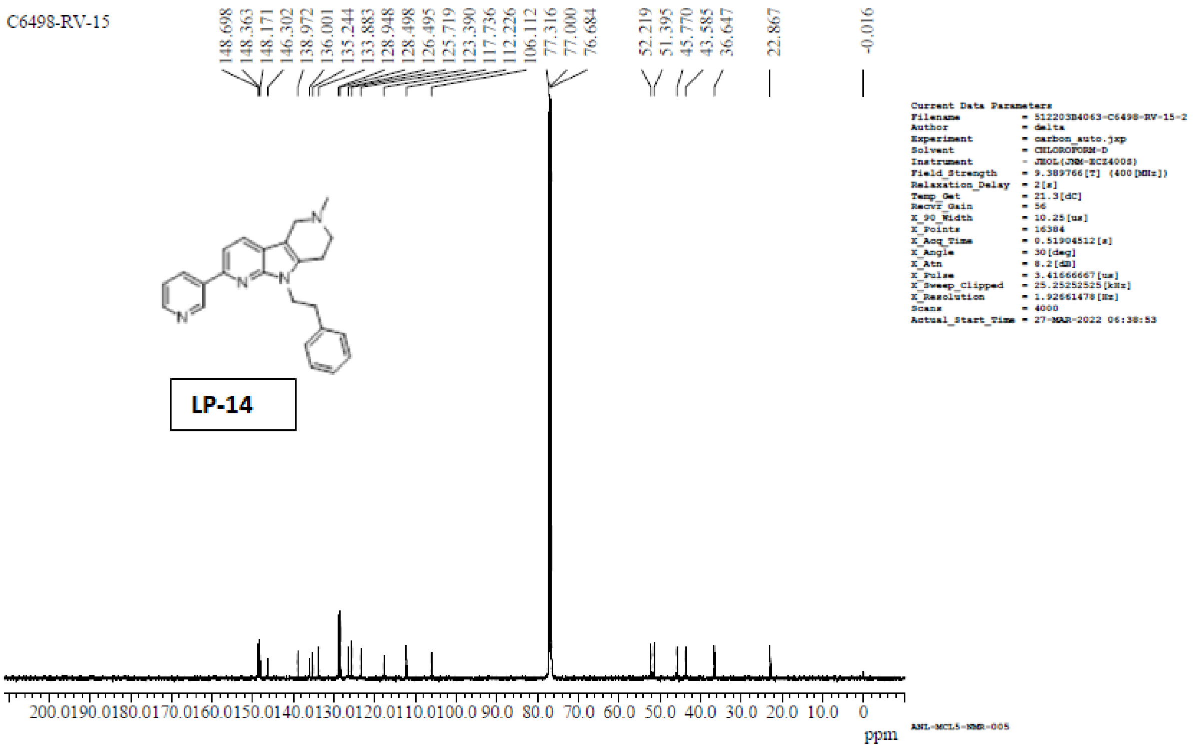

^{13}CNMR spectrum of 6-Methyl-9-phenethyl-2-(pyridin-3-yl)-6,7,8,9-tetrahydro-5H-pyrrolo[2,3-b:4,5-c']dipyridine (LP-14) in $CDCl_3$ (100 MHz)

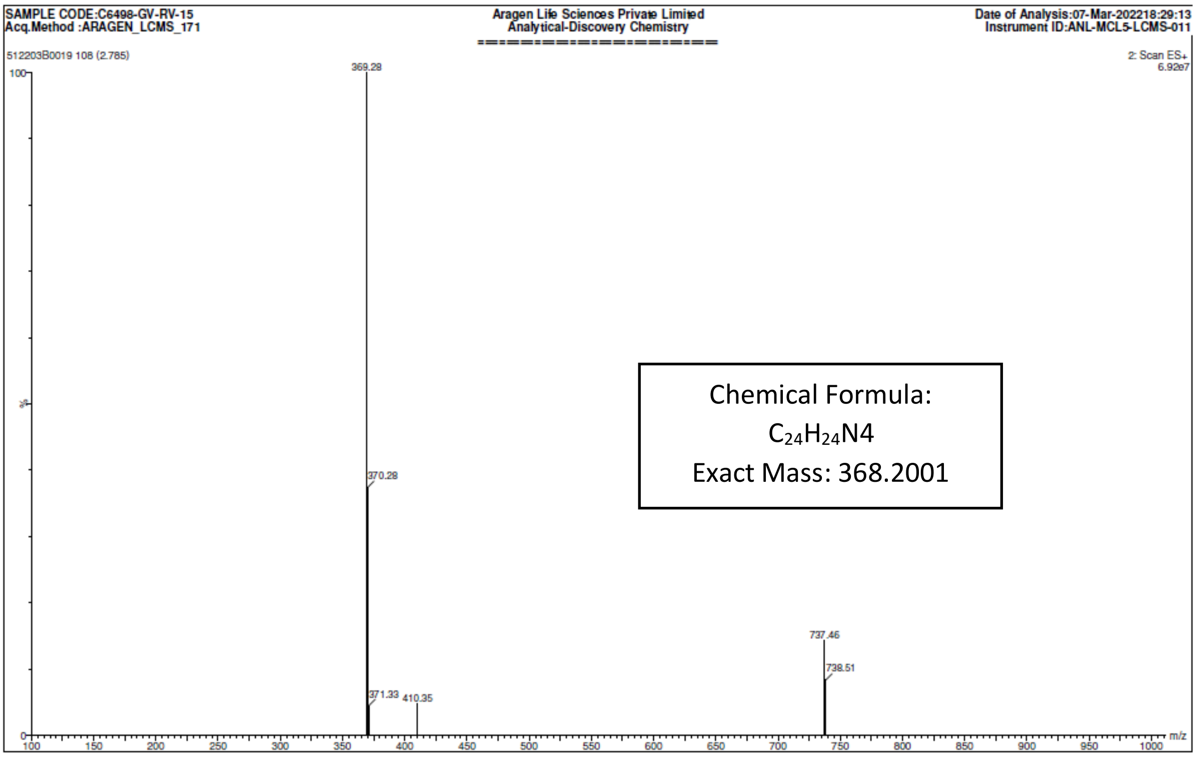

MASS spectrum of 6-Methyl-9-phenethyl-2-(pyridin-3-yl)-6,7,8,9-tetrahydro-5H-pyrrolo[2,3-b:4,5-c']dipyridine (LP-14)

Elemental Composition Report

Page 1

Single Mass Analysis

Tolerance = 820.0 PPM / DBE: min = -1.5, max = 50.0
Element prediction: Off
Number of isotope peaks used for i-FIT = 3

Monoisotopic Mass, Even Electron Ions
3 formula(e) evaluated with 1 results within limits (up to 50 best isotopic matches for each mass)
Elements Used:
C: 0-24 H: 0-25 N: 0-4

C6498-15
Acq.method formic acid_4min

Aragene Life sciences Private Limited
Analytical-Discovery Chemistry

09-Sep-2022
00:10:49
INSTRUMENT-ID;ANL-MCL5-LCMS-013

512209B4311 48 (1.224)

1: Scan ES+
2.24e+007

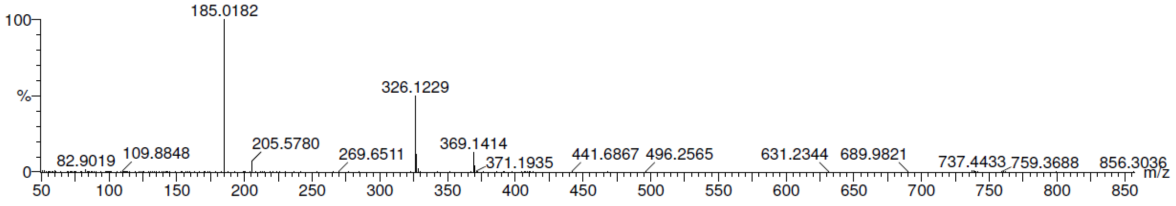

Minimum:				-1.5				
Maximum:		5.0	820.0	50.0				
Mass	Calc. Mass	mDa	PPM	DBE	i-FIT	Norm	Conf(%)	Formula
369.2069	369.2079	-1.0	-2.7	14.5	40.6	n/a	n/a	C24 H25 N4

Elemental composition report of 6-Methyl-9-phenethyl-2-(pyridin-3-yl)-6,7,8,9-tetrahydro-5H-pyrrolo[2,3-b:4,5-c']dipyridine (LP-14)

C6498-GV-RV-16

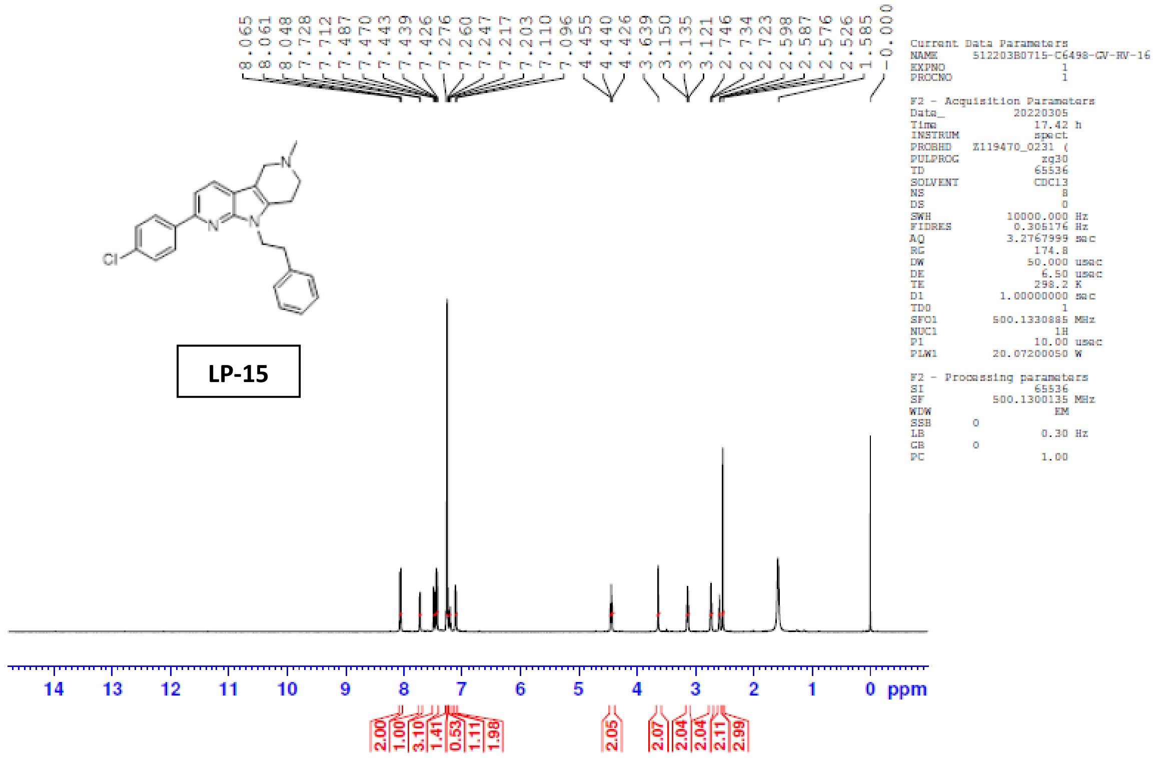

^{1}HNMR spectrum of 2-(4-Chlorophenyl)-6-methyl-9-phenethyl-6,7,8,9-tetrahydro-5H-pyrrolo[2,3-b:4,5-c']dipyridine (LP-15) in $CDCl_3$ (500 MHz)

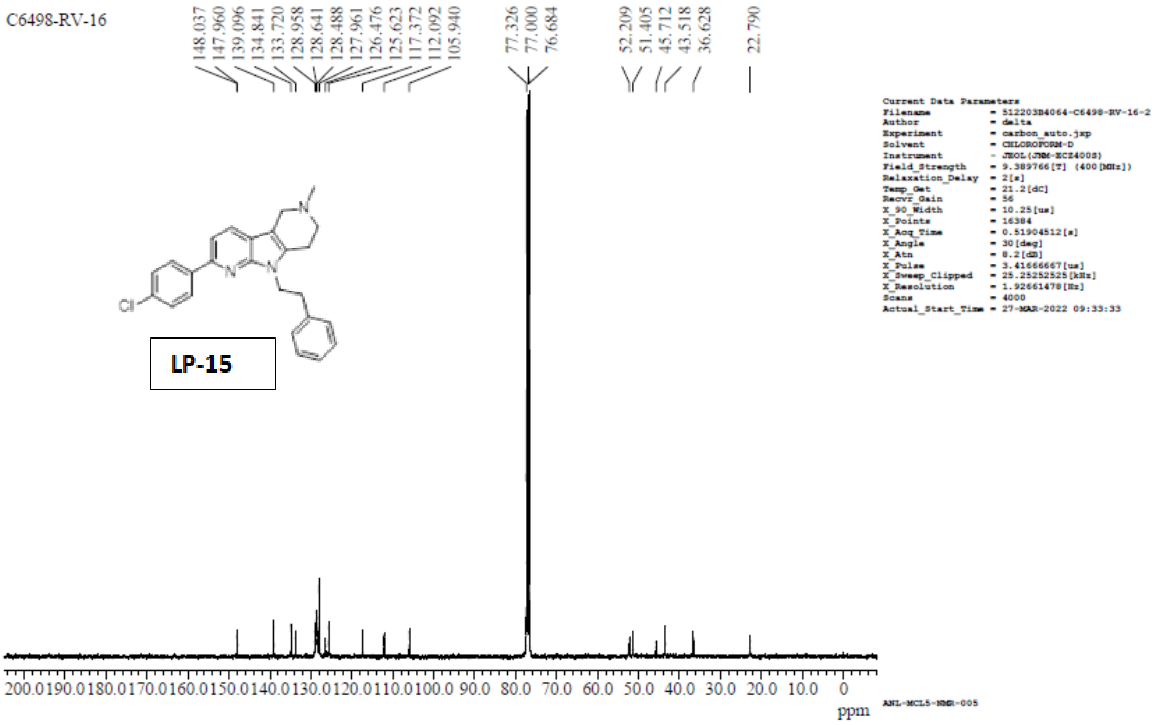

^{13}CNMR spectrum of 2-(4-Chlorophenyl)-6-methyl-9-phenethyl-6,7,8,9-tetrahydro-5H-pyrrolo[2,3-b:4,5-c']dipyridine (LP-15) in $CDCl_3$ (100 MHz)

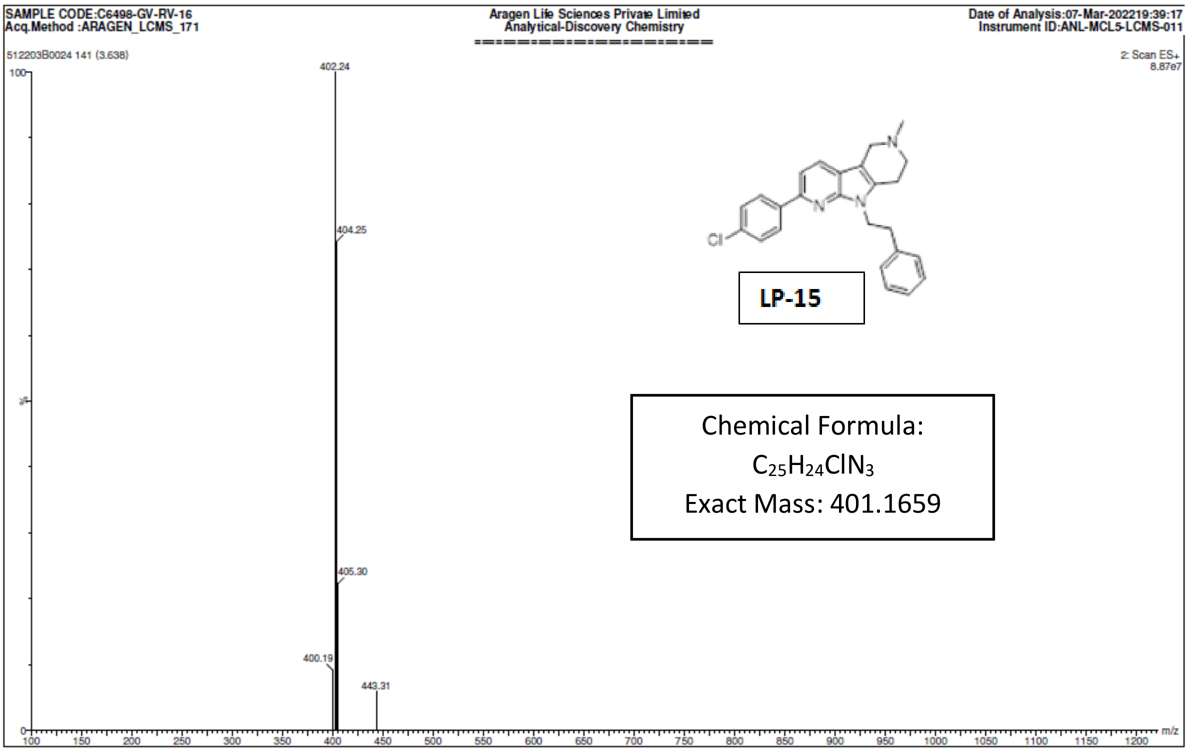

MASS spectrum of 2-(4-Chlorophenyl)-6-methyl-9-phenethyl-6,7,8,9-tetrahydro-5H-pyrrolo[2,3-b:4,5-c']dipyridine (LP-15)

Elemental Composition Report **Page 1**

Single Mass Analysis
Tolerance = 820.0 PPM / DBE: min = -1.5, max = 50.0
Element prediction: Off
Number of isotope peaks used for i-FIT = 3

Monoisotopic Mass, Even Electron Ions
5 formula(e) evaluated with 1 results within limits (up to 50 best isotopic matches for each mass)
Elements Used:
C: 0-25 H: 0-25 N: 0-3 Cl: 0-1

C6498-16
Acq.method formic acid_4min

Aragene Life sciences Private Limited
Analytical-Discovery Chemistry

09-Sep-2022
00:15:44
INSTRUMENT-ID;ANL-MCL5-LCMS-013

512209B4312 75 (1.923)

1: Scan ES+
2.68e+007

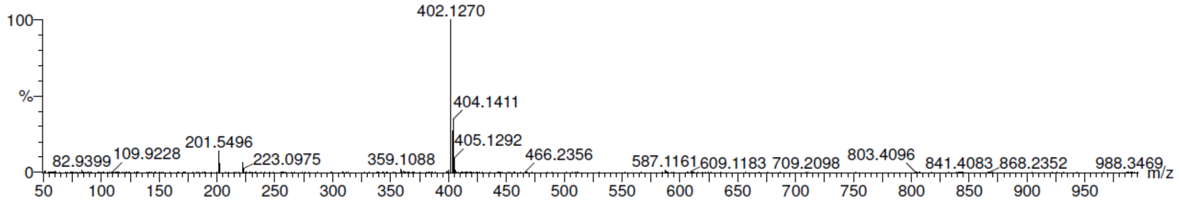

Minimum:				-1.5				
Maximum:		5.0	820.0	50.0				
Mass	Calc. Mass	mDa	PPM	DBE	i-FIT	Norm	Conf(%)	Formula
402.1730	402.1737	-0.7	-1.7	14.5	44.3	n/a	n/a	C25 H25 N3 Cl

Elemental composition report of 2-(4-Chlorophenyl)-6-methyl-9-phenethyl-6,7,8,9-tetrahydro-5H-pyrrolo[2,3-b:4,5-c']dipyridine (LP-15)

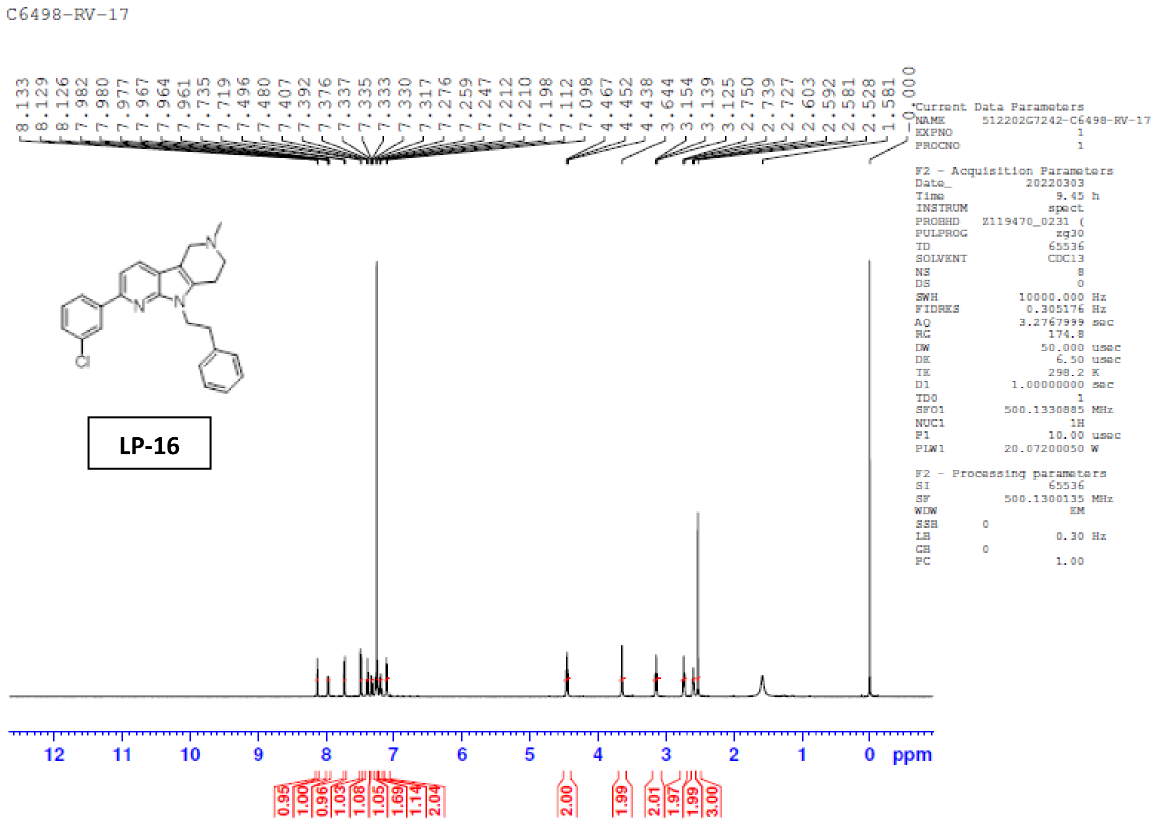

^{1}HNMR spectrum of 2-(3-Chlorophenyl)-6-methyl-9-phenethyl-6,7,8,9-tetrahydro-5H-pyrrolo[2,3-b:4,5-c']dipyridine (LP-16) in $CDCl_3$ (500 MHz)

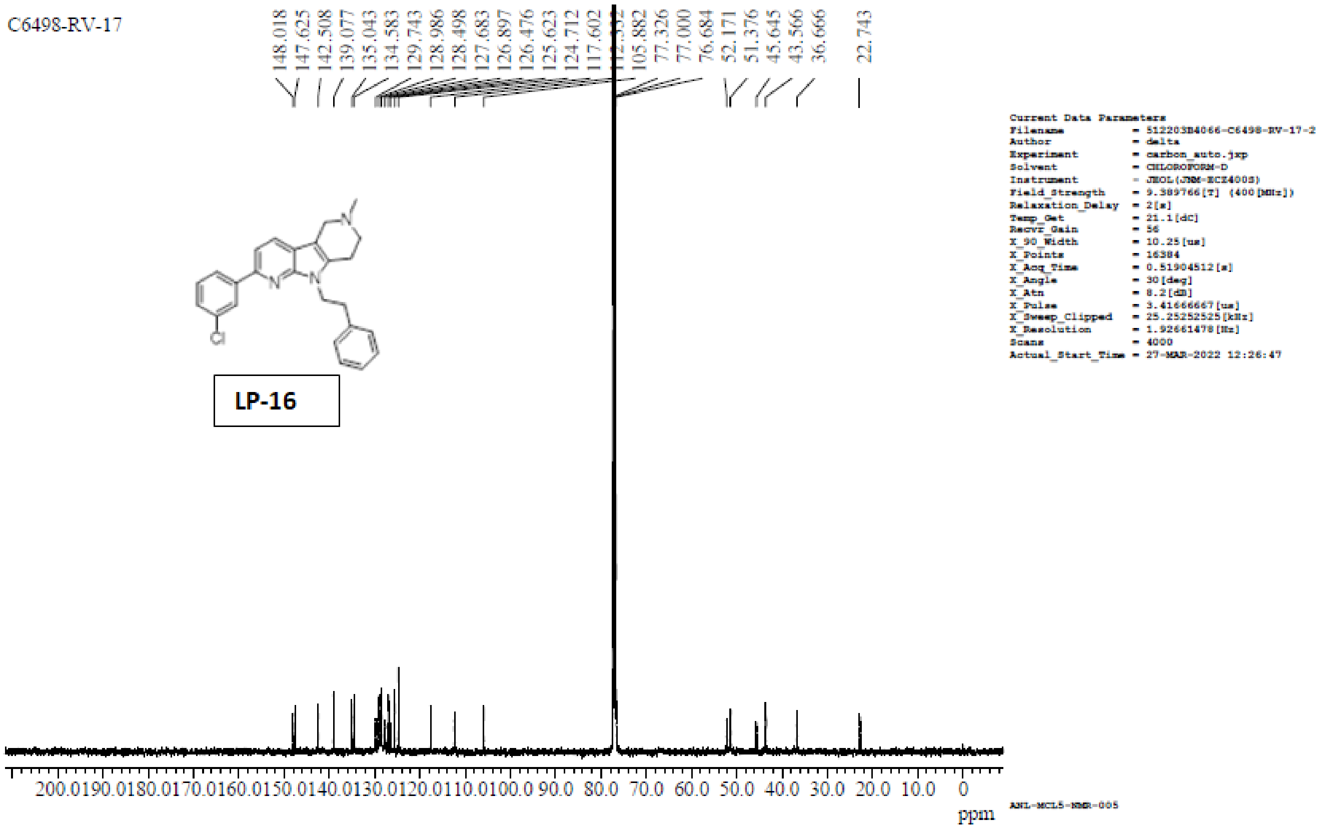

^{13}CNMR spectrum of 2-(3-Chlorophenyl)-6-methyl-9-phenethyl-6,7,8,9-tetrahydro-5H-pyrrolo[2,3-b:4,5-c']dipyridine (LP-16) in $CDCl_3$ (100 MHz)

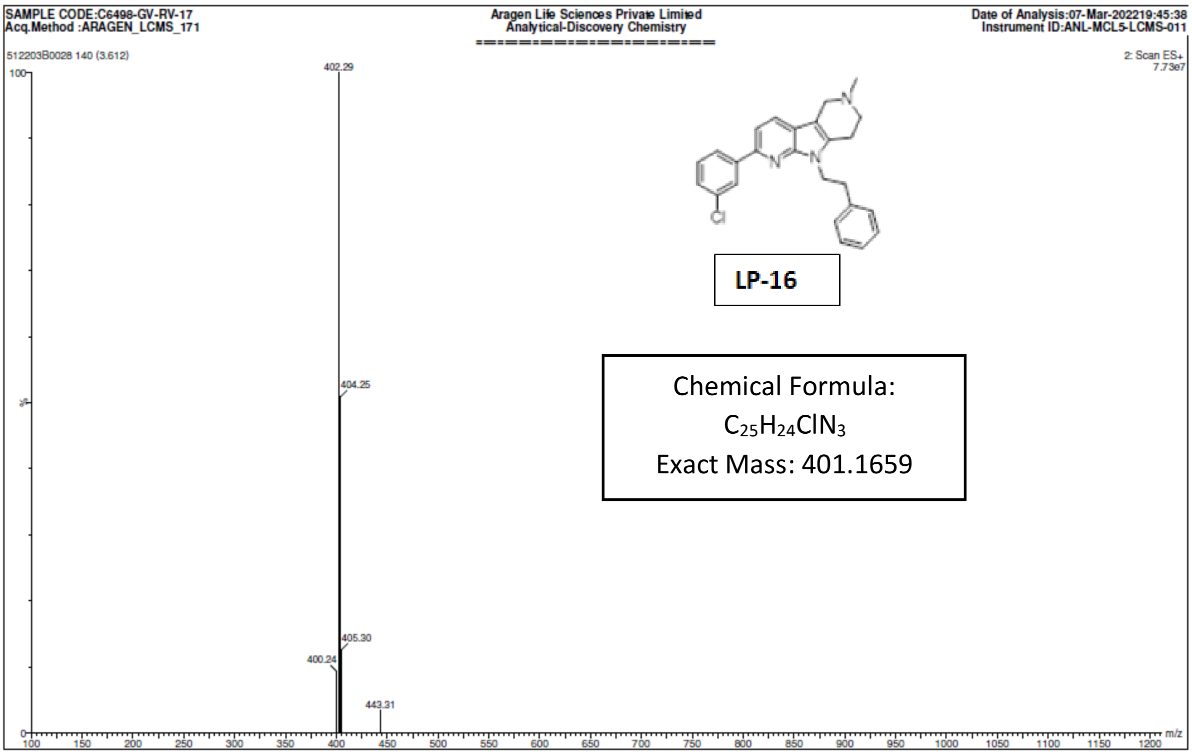

MASS spectrum of 2-(3-Chlorophenyl)-6-methyl-9-phenethyl-6,7,8,9-tetrahydro-5H-pyrrolo[2,3-b:4,5-c']dipyridine (LP-16)

Elemental Composition Report **Page 1**

Single Mass Analysis
Tolerance = 820.0 PPM / DBE: min = -1.5, max = 50.0
Element prediction: Off
Number of isotope peaks used for i-FIT = 3

Monoisotopic Mass, Even Electron Ions
5 formula(e) evaluated with 1 results within limits (up to 50 best isotopic matches for each mass)
Elements Used:
C: 0-25 H: 0-25 N: 0-3 Cl: 0-1

C6498-17
Acq.method formic acid_4min

Aragene Life sciences Private Limited
Analytical-Discovery Chemistry
==

09-Sep-2022
00:20:40
INSTRUMENT-ID;ANL-MCL5-LCMS-013
1: Scan ES+
2.77e+007

512209B4313 75 (1.923)

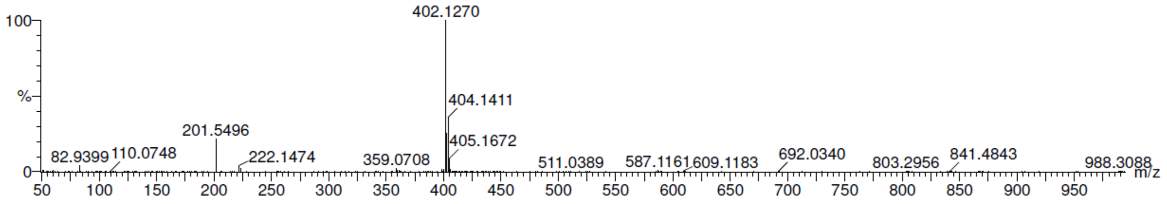

Minimum:				-1.5				
Maximum:		5.0	820.0	50.0				
Mass	Calc. Mass	mDa	PPM	DBE	i-FIT	Norm	Conf(%)	Formula
402.1730	402.1737	-0.7	-1.7	14.5	47.4	n/a	n/a	C25 H25 N3 Cl

Elemental composition report of 2-(3-Chlorophenyl)-6-methyl-9-phenethyl-6,7,8,9-tetrahydro-5H-pyrrolo[2,3-b:4,5-c']dipyridine (LP-16)
